# Supplementary material for: Seroprevalence and risk factors of hepatitis B virus infection among healthcare workers in Africa: A systematic review and meta-analysis
Source: PLoS One. 2025 Mar 25;20(3):e0319986. doi: 10.1371/journal.pone.0319986 (PMC11936272; doi:10.1371/journal.pone.0319986)
Supplement: S2 Table — (DOCX) [file pone.0319986.s002.docx]

**JBI Quality Assessment Criteria for cross sectional study**

| Included articles | Criteria | | | | | | | | | |
| --- | --- | --- | --- | --- | --- | --- | --- | --- | --- | --- |
|  | Clear eligibility criteria | Description of study subject and study setting | Valid & reliable method to measure the exposure | Standard criteria used for measurement of the condition | Identification of confounding factors | Development of strategies to deal with confounding factors | Valid and reliable method to measure outcomes | Appropriate statistical analysis | Total score out of (n=8) | Quality Score (100%) |
| Ajayl et al, 2007 | NO | YES | NO | YES | NO | NO | NO | YES | 3 | 37.5 |
| Akalu et al, 2016 | YES | YES | YES | NO | NO | YES | YES | YES | 7 | 75 |
| Akazong et al, 2020 | NO | YES | NO | YES | NO | NO | YES | YES | 4 | 50 |
| Alese et al, 2016) | YES | YES | YES | YES | NO | NO | YES | NO | 5 | 62.5 |
| Alitubeera et al, 2021 | NO | YES | NO | NO | NO | NO | YES | YES | 3 | 37.5 |
| Amsalu et al, 2016 | YES | YES | NO | YES | NO | NO | YES | YES | 5 | 62.5 |
| Anthony et al, 2023 | YES | NO | NO | YES | NO | NO | NO | YES | 3 | 37.5 |
| Ayele et al, 2023 | YES | YES | NO | YES | NO | YES | YES | YES | 6 | 75 |
| Belo et al, 2000 | YES | YES | NO | YES | NO | NO | YES | YES | 5 | 62.5 |
| Braka et al, 2006 | NO | YES | NO | YES | NO | NO | YES | YES | 4 | 50 |
| Djeriri et al, 2008 | NO | YES | NO | YES | NO | NO | YES | NO | 3 | 37.5 |
| Efua et al, 2023 | YES | YES | NO | YES | NO | YES | YES | YES | 6 | 75 |
| Elduma et al, 2011 | NO | YES | NO | YES | NO | NO | NO | YES | 3 | 37.5 |
| Elikwu et al, 2016 | YES | YES | NO | YES | NO | NO | NO | YES | 4 | 50 |
| Elmaghloub et al, 2017 | YES | NO | NO | YES | NO | NO | NO | YES | 3 | 37.5 |
| Elmukashfi et al, 2012 | NO | YES | NO | YES | NO | NO | NO | YES | 3 | 37.5 |
| Elzouki et al, 2014 | NO | NO | NO | YES | NO | NO | YES | YES | 3 | 37.5 |
| Yilma et al, 2019 | NO | YES | NO | YES | NO | YES | YES | YES | 5 | 62.5 |
| Kateera et al, 2015 | YES | YES | NO | YES | NO | NO | YES | NO | 4 | 50 |
| Kefeni et al, 1989 | NO | NO | NO | NO | YES | NO | YES | YES | 3 | 37.5 |
| Kisangau et al, 2018 | YES | YES | NO | YES | NO | NO | YES | YES | 5 | 62.5 |
| Lied et al, 2021 | NO | YES | NO | NO | NO | NO | NO | YES | 2 | 25 |
| Lungosi et al, 2019 | NO | NO | NO | YES | NO | YES | YES | YES | 4 | 50 |
| Mabunda et al, 2022 | YES | YES | NO | YES | NO | NO | YES | YES | 5 | 62.5 |
| Machange et al, 2017 | NO | YES | NO | YES | NO | NO | YES | YES | 4 | 50 |
| Massaquoi et al, 2022 | NO | YES | NO | YES | NO | NO | YES | YES | 4 | 50 |
| Mbaawuaga et al, 2019 | YES | YES | NO | YES | NO | NO | YES | YES | 5 | 62.5 |
| Mboya et al, 2023 | NO | YES | NO | YES | NO | YES | YES | YES | 5 | 62.5 |
| Mekonen et al, 2015 | NO | YES | NO | YES | NO | YES | YES | YES | 5 | 62.5 |
| Abate et al, 2022 | YES | NO | NO | YES | NO | NO | NO | YES | 3 | 37.5 |
| Mengiste et al, 2020 | NO | YES | NO | YES | NO | YES | YES | YES | 5 | 62.5 |
| Mueller et al, 2015 | NO | YES | NO | YES | NO | NO | NO | YES | 3 | 37.5 |
| Nail et al, 2008 | NO | YES | NO | YES | NO | NO | YES | YES | 4 | 50 |
| Ndako et al, 2014 | YES | NO | NO | NO | NO | NO | YES | YES | 3 | 37.5 |
| Qin et al, 2018 | NO | YES | NO | YES | NO | NO | YES | YES | 4 | 50 |
| Sani et al, 2018 | NO | YES | NO | YES | NO | NO | YES | YES | 4 | 50 |
| Shao et al, 2018 | YES | YES | NO | YES | NO | NO | YES | YES | 5 | 62.5 |
| Ssekamatte et al, 2020 | NO | YES | YES | YES | NO | NO | NO | NO | 3 | 37.5 |
| Yizengaw et al, 2018 | YES | YES | NO | YES | NO | YES | YES | YES | 6 | 75 |
| Ziraba et al, 2010 | YES | YES | NO | YES | NO | YES | YES | YES | 6 | 75 |
