# Supplementary material for: Seroprevalence and risk factors of hepatitis B virus infection among healthcare workers in Africa: A systematic review and meta-analysis
Source: PLoS One. 2025 Mar 25;20(3):e0319986. doi: 10.1371/journal.pone.0319986 (PMC11936272; doi:10.1371/journal.pone.0319986)
Supplement: S3 Table — (DOCX) [file pone.0319986.s003.docx]

**List of studied identified from various data bases**

| Sr. No | Title of the article/study/document | Inclusion status | Reason for exclusion (if excluded) |
| --- | --- | --- | --- |
|  | Prevention of hepatitis B and C in the EU/EEA April 2024 | Not included | It is not study |
|  | Sero-Prevalence of HBV and its Associated Factors Among Healthcare Providers in Public Health Facilities in Eastern Ethiopia | Not included |  |
|  | An Assessment of the Knowledge, Attitude, and Practice Toward Standard Precautions Among Health Workers From a Hospital in Northern Cyprus | Not included | Unrelated outcome |
|  | Burden of Hepatitis-B Infections and Risk Factors among Healthcare Workers in Resource Limited Setting, Addis Ababa, Ethiopia | Included |  |
|  | Knowledge, attitude and prevalence of hepatitis B virus among healthcare workers: a cross- sectional, Hospital based study in Bamenda Health District, NWR, Cameroon | Included |  |
|  | Seroprevalence of Hepatitis B Surface Antigen and Occupational Risk Factors Among Health Care Workers in Ekiti State, Nigeria | Not included |  |
|  | Epidemiology of viral hepatitis and HIV co-infection | Not included |  |
|  | The exposure rate to hepatitis B and C viruses among medical waste handlers in three government hospitals, southern Ethiopia | Included |  |
|  | Assessment of Knowledge, Attitude and Vaccination Status of Hepatitis B among Nursing Training Students in Ho, Ghana | Not included | Unrelated outcome |
|  | Precautions for health care workers to avoid hepatitis B and C virus infection | Not included | Unrelated outcome |
|  | Serological Evidence and Associated Factors of Hepatitis B Virus and Hepatitis C Virus Among Waste Handlers: A Cross-Sectional Study from Northeastern Ethiopia | Included |  |
|  | Systematic review and meta-analysis of HIV, HBV and HCV infection prevalence in Sudan | Not included | Study is review |
|  | Hepatitis B Virus Infection and Associated Factors Among Adults in Southwest Ethiopia: Community-Based Cross-Sectional Study | Not included | Study done on adult population |
|  | Prevalence of hepatitis B virus markers in surgeons in Lagos, Nigeria | Included |  |
|  | Risk and management of blood-borne infections in health care worker | Not included | Unrelated outcome |
|  | Hepatitis B vaccination status and needle-stick and sharps-related Injuries among medical school students in Nepal: a cross-sectional study | Not included | Unrelated outcome |
|  | Seroprevalence of hepatitis B virus infection in Cameroon: a systematic review and meta-analysis | Not included | Study is review |
|  | A historical perspective on the discovery and elucidation of the hepatitis B virus | Not included | Unrelated outcome |
|  | Hepatitis B infection among health workers in Uganda: evidence of the need for health worker protection | Included |  |
|  | Hepatitis B Virus Blood Screening: Need for Reappraisal of Blood Safety Measures? | Not included | Unrelated outcome |
|  | Coverage of hepatitis B vaccination in Swedish healthcare workers | Not included | Unrelated outcome |
|  | Prevalence of hepatitis B surface antigen (HbsAg) among health professionals in public Hospitals in Addis Ababa, Ethiopia | Not included | Do not fulfill the quality approval |
|  | Hepatitis B and human immunodeficiency virus co-infection among pregnant women in resource-limited high endemic setting, Addis Ababa, Ethiopia: implications for prevention and control measures | Not included | Unrelated outcome |
|  | Knowledge, Attitude, and Practice towards Hepatitis B Infection Prevention and Screening among Indonesians | Not included | Unrelated outcome |
|  | Epidemiological patterns of hepatitis B virus (HBV) in highly endemic areas | Not included | Unrelated outcome |
|  | Seroprevalence of Hepatitis B virus infection and associated factors among health care workers in Southern Ghana | Included |  |
|  | Economic burden of hepatitis C-associated diseases: Europe, Asia Pacific, and the Americas | Not included | Unrelated outcome |
|  | Seroprevalence of hepatitis B surface antigenaemia among healthcare worker in a private Nigerian tertiary health institution | Included |  |
|  | Hepatitis B Virus Infection in Pregnant Women, in Al Fashir Town, North Darfur State, Sudan | Not included | Study done on other population |
|  | Epidemiologic and socioeconomic factors impacting hepatitis B virus and related hepatocellular carcinoma | Not included | Unrelated outcome |
|  | Prevalence and correlates of hepatitis B and C seropositivity among health care workers in a semi urban setting in North Central Nigeria | Not included | Quality appraisal |
|  | Epidemiology of viral hepatitis in Somalia: Systematic review and meta-analysis study | Not included | Study is not primary study |
|  | The global burden of disease attributable to contaminated injections given in health care settings | Not included | Study not specific to HBV |
|  | Hepatitis B and C Viral Infection: Prevalence, Knowledge, Attitude, Practice, and Occupational Exposure among Healthcare Workers of Jimma University Medical Center, Southwest Ethiopia | Not included |  |
|  | Prevalence and predictors of hepatitis B virus (HBV) infection in east Africa: evidence from a systematic review and meta-analysis of epidemiological studies published from 2005 to 2020 | Not included | It is not primary study |
|  | Hepatitis B and C seroprevalence among health care workers in a tertiary hospital in Rwanda | Included |  |
|  | Prevalence of hepatitis B virus infection and uptake of hepatitis B vaccine among healthcare workers, Makueni County, Kenya 2017 | Included |  |
|  | High prevalence of hepatitis B infections in Burkina Faso (1996-2017): a systematic review with meta-analysis of epidemiological studies | Not included | It is not primary study |
|  | Impact of the national hepatitis B immunization program in China: a modeling study | Not included | Unrelated outcome |
|  | Changing prevalence of chronic hepatitis B virus infection in China between 1973 and 2021: a systematic literature review and meta-analysis of 3740 studies and 231 million people | Not included | It is not primary study |
|  | Assessing the prevalence of hepatitis B virus infection among health care workers in a referral hospital in Kisantu, Congo DR: a pilot study | Included |  |
|  | Seroprevalence of Hepatitis B Among Healthcare Workers in Asia and Africa and Its Association With Their Knowledge and Awareness: A Systematic Review and Meta-Analysis | Not included | It is not primary study |
|  | Prevalence of hepatitis B virus and immunity status among healthcare workers in Beira City, Mozambique | Included |  |
|  | Seroprevalence and Knowledge of Hepatitis B Virus Infection Among Laboratory Workers at Kilimanjaro Christian Medical Centre in Moshi, Tanzania | Included |  |
|  | Hepatitis B virus in Lao dentists: A cross-sectional serological study | Not included |  |
|  | Cross sectional study of chronic hepatitis B prevalence among healthcare workers in an urban setting, Sierra Leone | Included |  |
|  | Distribution of hepatitis B virus genotypes among patients with chronic infection in Japan shifting toward an increase of genotype A | Not included | Study done on other population |
|  | Hepatitis B Virus and Human Immunodeficiency Virus Infections among Health Care Workers in Some Health Care Centers in Benue State, Niger | Included |  |
|  | Hepatitis B virus infection status and associated factors among health care workers in selected hospitals in Kisumu County, Kenya: A cross-sectional study | Included |  |
|  | Prevalence of HBV, HCV and Associated Risk Factors Among Cleaners at Selected Public Health Centers in Addis Ababa, Ethiopia | Included |  |
|  | Hepatitis B virus infection and its associated factors among medical waste collectors at public health facilities in eastern Ethiopia: a facility-based cross-sectional study | Included |  |
|  | Prevalence of hepatitis B virus and associated risk factors among adults patients at Dessie referral and Kemise general hospitals in northeastern Ethiopia | Not included | Study done on other population |
|  | Seroprevalence of hepatitis B surface antigen and anti HCV antibody and its associated risk factors among pregnant women attending maternity ward of Felege Hiwot Referral Hospital, northwest Ethiopia: a cross-sectional study | Not included | Study done on other population |
|  | Prevalence of hepatitis B virus infection among health care workers in a tertiary hospital in Tanzania | Not included |  |
|  | Prevalence of hepatitis B virus infection in Nigeria, 2000-2013: a systematic review and meta-analysis | Not included | It is not primary study |
|  | Seroprevalence of Hepatitis B and C among health care workers in Omdurman, Sudan | Included |  |
|  | Hepatitis B Prevalence, Knowledge and Occupational Factors among Health Care Workers in Fako Division, South West Region Cameroon | Not included |  |
|  | An enormous hepatitis B virus-related liver disease burden projected in Vietnam by 2025 | Not included | Study done in other setting |
|  | Hepatitis B in Healthcare Personnel: An Update on the Global Landscape | Not included | It is not primary study |
|  | Hepatitis B infection is highly prevalent among patients presenting with jaundice in Kenya | Not included | Study done on other population |
|  | Hepatitis B in Ghana: a systematic review & meta-analysis of prevalence studies (1995-2015) | Not included | It is not primary study |
|  | Hepatitis B Fact sheet | Not included | It is not study |
|  | The prevalence of serological markers for hepatitis B virus infection in Australian Naval personnel | Not included | Study done in other setting |
|  | The prevalence of serological markers for hepatitis B virus infection in Australian Naval personnel | Not included | It is duplicates |
|  | Is universal HBV vaccination of healthcare workers a relevant strategy in developing endemic countries? The case of a university hospital in Niger | Not included | Unrelated outcome |
|  | Prevalence and associated knowledge of hepatitis B infection among healthcare workers in Freetown, Sierra Leone | Included |  |
|  | Enzyme-linked immunosorbent assay for the quantitative/qualitative analysis of plant secondary metabolites | Not included | Study not done on human |
|  | Occupational injury history and universal precautions awareness: a survey in Kabul hospital staff | Not included | Unrelated outcome |
|  | Seroprevalence of Hepatitis B and C among Healthcare Workers in Dutse Metropolis Jigawa State, Nigeria | Included |  |
|  | Estimations of worldwide prevalence of chronic hepatitis B virus infection: a systematic review of data published between 1965 and 2013 | Not included | It is not primary study |
|  | Seroprevalence of hepatitis B virus infection and associated factors among healthcare workers in northern Tanzania | Included |  |
|  | Impact of Training about Knowledge, Attitude and Practice of Hepatitis B infection and its Vaccination among Healthcare Professionals in a Tertiary Care Hospital of Northern Gujarat | Not included | Unrelated outcome |
|  | A new approach to prevent, diagnose, and treat hepatitis B in Africa | Not included | Unrelated outcome |
|  | Effectiveness of Hepatitis B Vaccination Campaign in Italy: Towards the Control of HBV Infection for the First Time in a European Country | Not included | Unrelated outcome |
|  | Prevalence of central obesity and associated factors in Ethiopia: A systematic review and meta-analysis | Not included | Unrelated study |
|  | Global, regional, and national incidence, prevalence, and years lived with disability for 301 acute and chronic diseases and injuries in 188 countries, 1990-2013: a systematic analysis for the Global Burden of Disease Study 2013 | Not included | It is not primary study |
|  | Hepatitis | Not included | It is not study |
|  | Health care worker safety | Not included | It is not study |
|  | Global health sector strategy on Viral hepatitis 2016–2021 | Not included | It is not study |
|  | Global Hepatitis Report | Not included | It is not study |
|  | Overcoming fragility in Somalia to build a strong primary health care system | Not included | Unrelated study |
|  | Occupational health: health workers | Not included | It is not study |
|  | Hepatitis B | Not included | It is not study |
|  | Global hepatitis report: Action for access in low- and middle-income countries | Not included | It is not primary study |
|  | Sero-Prevalence and Associated Factors of Hepatitis B Virus Infection among Health Professionals in Adama Town, Oromia, Central Ethiopia | Included |  |
|  | Sero-prevalence of hepatitis B virus infection and associated factors among health care workers and medical waste handlers in primary hospitals of North-west Ethiopia | Included |  |
|  | Seroprevalence and risk factors for hepatitis B infection in an adult population in Northeast China | Not included | Study done in other setting |
|  | Let's Talk About B: Barriers to Hepatitis B Screening and Vaccination Among Asian and South Asian Immigrants in British Columbia | Not included | Unrelated outcome |
|  | Sero-prevalence and risk factors for hepatitis B virus infection among health care workers in a tertiary hospital in Uganda | Included |  |
|  | Gamma-glutamyl transpeptidase-to-platelet ratio and the fibrosis-4 index in predicting hepatitis B virus-related hepatocellular carcinoma development in elderly chronic hepatitis B patients in China: A single-center retrospective study | Not included | Unrelated study |
|  | Epidemiology of hepatitis B virus infection: results from a community-based study of 0.15 million residents in South China | Not included | Study done in other setting |
|  | Sero-prevalence and risk factors of hepatitis B virus and human immunodeficiency virus infection among pregnant women in Bahir Dar city, Northwest Ethiopia: a cross sectional study | Not included | Study done in other population |
|  | Out of hospital cardiac arrest: when to resuscitat | Not included | Unrelated study |
|  | Sharps injuries among health care workers in Cairo University Hospitals | Not included | Outcome not measured clearly |
|  | Impact of Occupational Hazards on Healthcare Professionals' Mental (Psychological) Health: Evidence from Government-Owned Hospitals in Khulna, Bangladesh | Not included | Outcome not measured clearly |
|  | Health Profiles of Newly Arrived Refugee Children in the United States, 2006-2012 | Not included | Study done in other population and setting |
|  | Cardiac health in patients with hepatitis B virus-related cirrhosis | Not included | Study done in other population |
|  | Prevalence and Associated Factors of Human Papillomavirus Infection among Iraqi Women | Not included | Unrelated study |
|  | Hepatitis C Virus Cascades of Care in the era of Direct-Acting Antiviral Therapy | Not included | Unrelated study |
|  | Maternal health in China - challenges of the next decade | Not included | Unrelated study |
|  | A study on prevention of bleeding complications using lusutrombopag for safe RFA in patients with hepatocellular carcinoma with low platelet counts: prospective observational study | Not included | Unrelated study |
|  | Validation of the Korean Stroop Test in Diagnosis of Minimal Hepatic Encephalopathy | Not included | Unrelated study |
|  | Seroprevalence and predictors of hepatitis B virus infection among pregnant women attending routine antenatal care in Arba Minch Hospital, South Ethiopia | Not included | Study done in other population |
|  | Conservation and variability of hepatitis B core at different chronic hepatitis stages | Not included | Unrelated outcome |
|  | Prevalence of Hepatitis B virus infection and its determinants among pregnant women in East Africa: Systematic review and Meta-analysis | Not included | It is not a primary study |
|  | HCC risk reduction with oral nucleos(t)ide analogues in patients with chronic hepatitis B: Not perfect, not good enough | Not included | Unrelated outcome |
|  | Occupational exposures to blood and body fluids (BBFS) among health care workers and medical students in University of Gondar Hospital, Northwest of Ethiopia | Not included | Outcome not measured clearly |
|  | Somatosensory Amplification, Anxiety, and Depression in Patients With Hepatitis B: Impact on Functionality | Not included | Unrelated outcome |
|  | Health behaviors of Korean adults with hepatitis B: Findings of the 2016 Korean National Health and Nutrition Examination Survey | Not included | Unrelated outcome |
|  | Prevalence of and risk factors for hepatitis C virus antibody among people who inject drugs in Cambodia: a national biological and behavioral survey | Not included | Study done in other population |
|  | An updated systematic review and meta-analysis of the prevalence of hepatitis B virus in Ethiopia | Not included | It is not a primary study |
|  | Management of Takotsubo cardiomyopathy in non-academic hospitals in France: The Observational French SyndromEs of TakoTsubo (OFSETT) study | Not included | Unrelated study |
|  | Clinical characteristics, prognosis, and surgical outcomes of patients with non-HBV and non-HCV related hepatocellular carcinoma: three-decade observational study | Not included | Unrelated outcome |
|  | Characteristics of female sexual dysfunctions and obstetric complications related to female genital mutilation in Omdurman maternity hospital, Sudan | Not included | Unrelated study |
|  | Prognostic value of immunoscore to identify mortality outcomes in adults with HBV-related primary hepatocellular carcinoma | Not included | Unrelated outcome |
|  | The association of adverse outcomes in the mother with disease progression in offspring in families with clusters of hepatitis B virus infection and unfavorable prognoses in Northwest China | Not included | Study done in other setting |
|  | Hospital acquired infections and infection prevention practices in teaching hospitals in the Amhara regional state, Ethiopia | Not included | Outcome not measured clearly |
|  | Sero-prevalence of hepatitis B virus infection and its risk factors among pregnant women attending antenatal clinic at Aminu Kano Teaching Hospital, Kano, Nigeria | Not included | Study done in other population |
|  | Relationship of Treg/Th17 balance with HBeAg change in HBeAg-positive chronic hepatitis B patients receiving telbivudine antiviral treatment: A longitudinal observational study | Not included | Unrelated outcome |
|  | Occupational exposures to blood and body fluids (BBFS) among health care workers and medical students in University of Gondar Hospital, Northwest of Ethiopia | Not included | Outcome not measured clearly |
|  | Telbivudine and adefovir dipivoxil combination therapy improves renal function in patients with chronic hepatitis B: A STROBE-compliant article | Not included | Outcome not measured clearly |
|  | Outcomes of Glucocorticoid Treatment in HBV Associated Acute-on-Chronic Liver Failure Patients: A Retrospective Observational Study | Not included | Study done in other population |
|  | Multimodal Ultrasound Model Based on the Left Gastric Vein in B-Viral Cirrhosis: Noninvasive Prediction of Esophageal Varices | Not included | Unrelated outcome |
|  | Multiple Primary Malignancies in Patients With Hepatocellular Carcinoma: A Largest Series With 26-Year Follow-Up | Not included | Unrelated outcome |
|  | Expression profiles of transcription factors for special CD4+ T-cell subsets in peripheral blood mononuclear cells from patients with hepatitis B virus infection | Not included | Unrelated outcome |
|  | Hepatitis B virus-associated hepatocellular carcinoma | Not included | Outcome not measured clearly |
|  | HBV pgRNA profiles in Chinese HIV/HBV coinfected patients under pre- and posttreatment: a multicentre observational cohort study | Not included | Study done in other setting |
|  | Population-Based Multicentric Survey of Hepatitis B Infection and Risk Factors in the North, South, and Southeast Regions of Brazil, 10-20 Years After the Beginning of Vaccination | Not included | Study done in general population |
|  | Analysis of serum hepatitis B virus RNA levels among HBsAg and HBsAb copositive patients and its correlation with HBV DNA | Not included | Study done in general population |
|  | Global Burden and Trends of Primary Liver Cancer Attributable to Comorbid Type 2 Diabetes Mellitus Among People Living with Hepatitis B: An Observational Trend Study from 1990 to 2019 | Not included | Study is not a primary study |
|  | Systemic immune-inflammation index predicts postoperative acute kidney injury in hepatocellular carcinoma patients after hepatectomy | Not included | Unrelated outcome |
|  | The imported infections among foreign travelers in China: an observational study | Not included | Study done in other setting |
|  | An updated systematic review and meta-analysis of the prevalence of hepatitis B virus in Ethiopia | Not included | Outcome not measured clearly |
|  | Hospital acquired infections and infection prevention practices in teaching hospitals in the Amhara regional state, Ethiopia | Not included | Outcome not measured clearly |
|  | 2022 International Consensus on Cardiopulmonary Resuscitation and Emergency Cardiovascular Care Science With Treatment Recommendations: Summary From the Basic Life Support; Advanced Life Support; Pediatric Life Support; Neonatal Life Support; Education, Implementation, and Teams; and First Aid Task Forces | Not included | Unrelated Study |
|  | Mercury Exposure and Poor Nutritional Status Reduce Response to Six Expanded Program on Immunization Vaccines in Children: An Observational Cohort Study of Communities Affected by Gold Mining in the Peruvian Amazon | Not included | Unrelated Study |
|  | Maternal exposure to carbon monoxide and fine particulate matter during pregnancy in an urban Tanzanian cohort | Not included | Unrelated Study |
|  | Sero-prevalence of hepatitis B virus infection and its risk factors among pregnant women attending antenatal clinic at Aminu Kano Teaching Hospital, Kano, Nigeria | Not included | Outcome not measured clearly |
|  | Hepatitis B virus-associated hepatocellular carcinoma | Not included | Outcome not measured clearly |
|  | Add-on pegylated interferon augments hepatitis B surface antigen clearance vs continuous nucleos(t)ide analog monotherapy in Chinese patients with chronic hepatitis B and hepatitis B surface antigen ≤ 1500 IU/mL: An observational study | Not included | Study done in other population |
|  | Pneumococcal and influenza vaccination coverage among at-risk adults: A 5-year French national observational study | Not included | Unrelated Study |
|  | A modified MELD model for Chinese pre-ACLF and ACLF patients and it reveals poor prognosis in pre-ACLF patients | Not included | Unrelated study |
|  | Analysis of serum hepatitis B virus RNA levels among HBsAg and HBsAb copositive patients and its correlation with HBV DNA | Not included | Outcome not measured clearly |
|  | Statins improve outcomes of nonsurgical curative treatments in hepatocellular carcinoma patients | Not included | Unrelated study |
|  | Genome-wide study of salivary microRNAs as potential noninvasive biomarkers for detection of nasopharyngeal carcinoma | Not included | Unrelated study |
|  | Comparison of the efficacy and safety of entecavir and tenofovir in nucleos(t)ide analogue-naive chronic hepatitis B patients with high viraemia: a retrospective cohort study | Not included | Outcome not measured clearly |
|  | Multicenter study of skin rashes and hepatotoxicity in antiretroviral-naïve HIV-positive patients receiving non-nucleoside reverse-transcriptase inhibitor plus nucleoside reverse-transcriptase inhibitors in Taiwan | Not included | Unrelated study |
|  | Methylation status of the stimulator of interferon genes promoter in patients with chronic hepatitis B | Not included | Unrelated outcome |
|  | Postnatal infection surveillance by telephone in Dar es Salaam, Tanzania: An observational cohort study | Not included | Outcome not measured clearly |
|  | MRI findings in people with epilepsy and nodding syndrome in an area endemic for onchocerciasis: an observational study | Not included | Unrelated study |
|  | Bacterial pathogenesis: a molecular approach | Not included | It is not study |
|  | Clinical features of treatment-naive patients with hepatitis B virus infection: A community-based survey from high- and intermediate-hepatitis B endemicity regions in Southeast China | Not included | Study done in other setting |
|  | Rituximab plus chemotherapy as first-line treatment in Chinese patients with diffuse large B-cell lymphoma in routine practice: a prospective, multicentre, non-interventional study | Not included | Unrelated study |
|  | Albumin-bilirubin and platelet-albumin-bilirubin grades for hepatitis B-associated hepatocellular carcinoma in Child-Pugh A patients treated with radical surgery: A retrospective observational study | Not included | Unrelated outcome |
|  | Hepatocellular carcinoma amongst aboriginal and torres strait islander peoples of Australia | Not included | Study done in other setting |
|  | Hepatitis B vaccination status and associated factors among undergraduate students of Makerere University College of Health Sciences | Not included | Study done in other population |
|  | Risk factors for underlying comorbidities and complications in patients with hepatitis B virus-related acute-on-chronic liver failure | Not included | Unrelated outcome |
|  | Psychological profiles of excluded living liver donor candidates: An observational study | Not included | Unrelated outcome |
|  | Prevalence of Hepatitis E Virus and Its Associated Outcomes among Pregnant Women in China | Not included | Study done in other population |
|  | Clinical cure induced by pegylated interferon α-2b in the advantaged population of chronic hepatitis B virus infection: a retrospective cohort study | Not included | Unrelated outcome |
|  | Prevalence and associated risk factors of Hepatitis B and Hepatitis C virus among volunteer blood donors in Arba Minch Blood Bank SNNPR, Ethiopia | Not included | Study done in other population |
|  | Genetic variation in FCER1A predicts peginterferon alfa-2a-induced hepatitis B surface antigen clearance in East Asian patients with chronic hepatitis B | Not included | Unrelated outcome |
|  | Cirrhosis and liver transplantation in patients co-infected with HIV and hepatitis B or C: an observational cohort study | Not included | Unrelated outcome |
|  | Mutations in pre-core and basic core promoter regions of hepatitis B virus in chronic hepatitis B patients | Not included | Unrelated outcome |
|  | Immune response pattern varies with the natural history of chronic hepatitis B | Not included | Unrelated outcome |
|  | Role of interleukin-21 and interleukin-21 receptor polymorphisms in the treatment of HBeAg-positive chronic hepatitis B patients with peginterferon | Not included | Unrelated outcome |
|  | The prevalence of hepatitis B virus infection in the United States in the era of vaccination | Not included | Study done in other setting |
|  | A Pilot Study of MicroRNAs Expression Profile in Serum and HBsAg Particles: Predictors of Therapeutic Vaccine Efficacy in Chronic Hepatitis B Patients | Not included | Study done in other population |
|  | An epidemiological survey of HBV infection and low-level HBsAg in military camps in eastern China | Not included | Study done in other setting |
|  | Epidemiology and etiology of diffuse large B-cell lymphoma | Not included | Unrelated study |
|  | Hepatitis B virus infection and related factors in hemodialysis patients in China–systematic review and meta-analysis | Not included | Study done in other setting and is not primary study |
|  | Prevalence of chronic obstructive pulmonary disease and associated risk factors in Uganda (FRESH AIR Uganda): a prospective cross-sectional observational study | Not included | Unrelated study |
|  | Stopping nucleos(t)ide analogue treatment in Caucasian hepatitis B patients after HBeAg seroconversion is associated with high relapse rates and fatal outcomes | Not included | Unrelated outcome |
|  | Caucasian Ethnicity, but Not Treatment Cessation is Associated with HBsAg Loss Following Nucleos(t)ide Analogue-Induced HBeAg Seroconversion | Not included | Unrelated outcome |
|  | Prevalence an d factors associated with hepatitis B susceptibility among men who sex with men on HIV pre-exposure prophylaxis in Northeastern Brazil: a cross-sectional study | Not included | Study done in other population |
|  | The Swiss STAR trial - an evaluation of target groups for sexually transmitted infection screening in the sub-sample of women | Not included | Unrelated outcome |
|  | Poor adherence and low persistency rates for hepatocellular carcinoma surveillance in patients with chronic hepatitis B | Not included | Outcome not measured clearly |
|  | Predictors of Hepatitis B Surface Antigen Titers two decades after vaccination in a cohort of students and post-graduates of the Medical School at the University of Palermo, Italy | Not included | Study done in other setting |
|  | High prevalence of hepatitis B virus and hepatitis D virus in the western Brazilian Amazon | Not included | Study done in other setting |
|  | Hepatitis B in Rondônia (Western amazon region, Brazil): descriptive analysis and spatial distribution | Not included | Study done in other setting |
|  | “I am still suffering:” The dilemma of multiple recoveries in the lives of methadone maintenance patients | Not included | It is not study |
|  | Syphilis, human immunodeficiency virus, herpes genital and hepatitis B in a women's prison in Cochabamba, Bolivia: prevalence and risk factors | Not included | Study done in other setting |
|  | Switch from intravenous or intramuscular to subcutaneous hepatitis B immunoglobulin: effect on quality of life after liver transplantation | Not included | Unrelated outcome |
|  | The value of APGA score, fibrosis index for diagnosing liver fibrosis in patients with chronic hepatitis B | Not included | Unrelated outcome |
|  | Epidemiological patterns and risk factors associated with hepatitis B virus in Pakistani population | Not included | Study done in other setting |
|  | Protocol: Prospective observational study investigating the prevalence and clinical outcome of portopulmonary hypertension in Japanese patients with chronic liver disease | Not included | Study done in other setting |
|  | Chapter 26 - Hepatitis A Vaccines | Not included | It is not study |
|  | Workplace hazards faced by nursing assistants in the United States: A focused literature review | Not included | Study done in other setting and is not primary study |
|  | Genotype Matters in Patients with Acute-on-chronic Liver Failure Due to Reactivation of Chronic Hepatitis B | Not included | Unrelated outcome |
|  | Sex difference in the associations among risk factors with hepatitis B and C infections in a large Taiwanese population study | Not included | Outcome not measured clearly |
|  | Protective effect of an improved immunization practice of mother-to-infant transmission of hepatitis B virus and risk factors associated with immunoprophylaxis failure | Not included | Outcome not measured clearly |
|  | Prevalence and factors associated with hepatitis B immunization and infection among men who have sex with men in Beijing, China | Not included | Study done in other setting |
|  | Comparison of hepatitis B virus and hepatitis C virus prevalence and risk factors in a community-based study | Not included | Outcome not measured clearly |
|  | Hepatitis C Virus Infection Associated With an Increased Risk of Deep Vein Thrombosis: A Population-Based Cohort Study | Not included | Unrelated study |
|  | Low immediate postoperative platelet count is associated with hepatic insufficiency after hepatectomy | Not included | Unrelated outcome |
|  | Clinical characteristics and risk factors of COVID-19 patients with chronic hepatitis B: a multi-center retrospective cohort study | Not included | Outcome not measured clearly |
|  | A statistical analysis of the correlations among various types of clinical indexes for patients with chronic hepatitis B: A hospital-based study | Not included | Outcome not clearly measured |
|  | Combined use of murine double minute-2 promoter methylation and serum AFP improves diagnostic efficiency in hepatitis B virus-related hepatocellular carcinoma | Not included | Outcome not related |
|  | Abnormal IL-10 levels were related to alanine aminotransferase abnormalities during postpartum in HBeAg positive women with chronic hepatitis B | Not included | Study done in other population |
|  | Gamma-glutamyl transpeptidase to platelet ratio index is a good noninvasive biomarker for predicting liver fibrosis in Chinese chronic hepatitis B patients | Not included | Study done in other setting |
|  | Risk factors for some tropical diseases in an African country | Not included | Outcome not clearly measured |
|  | Epidemiology of hepatitis B virus infection in Bangladesh: prevalence among general population, risk groups and genotype distribution | Not included | Study done in other population |
|  | Nosocomial infections: current situation in a resuscitation-unit | Not included | Outcome not measured clearly |
|  | Hepatitis B virus infection | Not included | Study done in other population |
|  | Does Nucleos(t)ide Analogues Treatment Affect Renal Function in Chronic Hepatitis B Patients Who Have Already Decreased eGFR? A Longitudinal Study | Not included | Outcome not related |
|  | Seroepidemiology of the human herpesvirus 8 infection among people living with HIV in Taiwan, 2014-2018 | Not included | Study done in other setting |
|  | Sero-prevalence and risk factors for hepatitis B virus infection among the consumers of the alcoholic beverage, cheka in Konso zone, southwestern Ethiopia | Not included | Study done in other population |
|  | Predictive factors for percutaneous and mucocutaneous exposure among healthcare workers in a developing country | Not included | Unrelated study |
|  | Prevalence and predictors of hepatitis B virus coinfection in a United States cohort of hepatitis C virus‐infected patients | Not included | Study done in other setting |
|  | Superiority of tenofovir alafenamide fumarate over entecavir for serum HBsAg level reduction in patients with chronic HBV infection: A 144-week outcome study after switching of the nucleos(t)ide analog | Not included | Outcome not clearly measured |
|  | The prevalence of hepatitis B and C viral infections among pregnant women | Not included | Study done in other population |
|  | Prevalence and risk factors of hepatitis B virus transmission among children in Enugu, Nigeria | Not included | Study done in other population |
|  | Hepatitis B virus infections and associated factors among pregnant women attending antenatal care clinic at Deder Hospital, Eastern Ethiopia | Not included | Study done in other population |
|  | Cross-sectional study of chronic hepatitis B virus infection in Rwandan high-risk groups: unexpected findings on prevalence and its determinants | Not included | Study done in other population |
|  | Screening a nation for hepatitis C virus elimination: a cross-sectional study on prevalence of hepatitis C and associated risk factors in the Rwandan general population | Not included | Unrelated outcome |
|  | Role of quantitative hepatitis B surface antigen in predicting inactive carriers and HBsAg seroclearance in HBeAg-negative chronic hepatitis B patients | Not included | Unrelated outcome |
|  | The role of Bcl-2 in hepatocarcinogenesis: Effects of overexpression on murine liver tumor development and hepatocyte cell cycle progression | Not included | Unrelated outcome |
|  | CHAPTER 12 - Alcohol and Substance Abuse | Not included | It is not study |
|  | Five‐year conditional survival for patients with hepatocellular carcinoma in Queensland, Australia | Not included | Study done in other setting |
|  | Admitted AIDS-associated Kaposi sarcoma patients: Indications for admission and predictors of mortality | Not included | Unrelated outcome |
|  | Sustained viral response and relapse after discontinuation of oral antiviral drugs in HBeAg-positive patients with chronic hepatitis B infection | Not included | Study done in other population |
|  | An observational study to evaluate the safety and efficacy of telbivudine in adults with chronic hepatitis B | Not included | Unrelated outcome |
|  | Clinical outcome indicators in chronic hepatitis B and C: A primer for value-based medicine in hepatology | Not included | Outcome not measured clearly |
|  | Patterns and co-occurrence of risk factors for hepatocellular carcinoma in four Asian American communities: a cross-sectional study | Not included | Study dome in other setting |
|  | A Prospective Study Evaluating Changes in Histology, Clinical and Virologic Outcomes in HBV-HIV Co-infected Adults in North America | Not included | Study dome in other setting |
|  | Long-term persistency of hepatitis B immunity: an observational cross-sectional study on medical students and resident doctors | Not included | Study dome in other population |
|  | Hepatitis B virus infection and factors associated with its acquisition among adults in a Lake Victoria HIV hyperendemic fishing community in Kyotera district, Uganda: a cross-sectional observation | Not included | Study dome in other population |
|  | Hepatitis B and C virus infection among 1.2 million persons with access to care: factors associated with testing and infection prevalence | Not included | Study dome in other population |
|  | Blood and virus detection on barber clippers | Not included | Study dome in other population |
|  | A new approach to prevent, diagnose, and treat hepatitis B in Africa | Not included | It is not primary study |
|  | Infectious diseases prevalence, vaccination coverage, and diagnostic challenges in a population of internationally adopted children referred to a Tertiary Care Children's Hospital from 2009 to 2015 | Not included | Study dome in other population |
|  | Renal angina index in critically ill children as an applicable and reliable tool in the prediction of severe acute kidney injury: Two tertiary centers' prospective observational study from the Middle East | Not included | Unrelated outcome |
|  | Associations Between Hepatitis B Virus Infection and Risk of All Cancer Types | Not included | Unrelated outcome |
|  | Off-treatment virologic relapse and outcomes of re-treatment in chronic hepatitis B patients who achieved complete viral suppression with oral nucleos(t)ide analogs | Not included | Unrelated outcome |
|  | Factors associated with sexually transmitted infections in sugarcane cutters: subsidies to caring for | Not included | Study dome in other population |
|  | Factors associated with immunoprophylaxis failure against vertical transmission of hepatitis B virus | Not included | Unrelated outcome |
|  | Hospital personnel sero-protected against hepatitis B virus following an accelerated vaccination program | Not included | Study dome in other population |
|  | Predictors of liver disease progression in people living with HIV-HBV co-infection on antiretroviral therapy | Not included | Study dome in other population |
|  | High prevalence of hepatitis B-antibody loss and a case report of de novo hepatitis B virus infection in a child after living-donor liver transplantation | Not included | Study dome in other population |
|  | Sexually transmitted infections among patients attending a sexual assault centre: a cohort study from Oslo, Norway | Not included | Unrelated outcome |
|  | Occupational risk perception in home health care workers | Not included | Unrelated outcome |
|  | Post-vaccination anti-HBs testing among healthcare workers: More economical than post-exposure management for Hepatitis B | Not included | Unrelated outcome |
|  | Blood donors and the supply of blood and blood products | Not included | It is not primary study |
|  | Pro-Inflammatory Interleukin-18 is Associated with Hepatic Steatosis and Elevated Liver Enzymes in People with HIV Monoinfection | Not included | Unrelated outcome |
|  | Sharps Injuries in Ambulatory Care: A Clinical Staff Perspective | Not included | Outcome not measured clearly |
|  | Seroevidence for a high prevalence of subclinical infection with avian influenza A (H5N1) virus among workers in a live-poultry market in Indonesia | Not included | Unrelated study |
|  | Effective therapeutic options for elderly patients with hepatocellular carcinoma: A nationwide cohort stud | Not included | Unrelated outcome |
|  | Risk Factors for Renal Functional Decline in Chronic Hepatitis B Patients Receiving Oral Antiviral Agents | Not included | Unrelated outcome |
|  | Prevalence and risk factors of hepatitis B virus, hepatitis C virus, and human immunodeficiency virus infections among drug addicts in Bangladesh | Not included | Study dome in other setting |
|  | Seroprevalence of hepatitis B virus infection and associated factors among prison inmates in state of Mato Grosso do Sul, Brazil | Not included | Study dome in other setting |
|  | Residual risk of mother-to-child transmission of hepatitis B virus infection despite timely birth-dose vaccination in Cameroon (ANRS 12303): a single-centre, longitudinal observational study | Not included | Unrelated outcome |
|  | Dually Active HIV/HBV Antiretrovirals as Protection Against Incident Hepatitis B Infections: Potential for Prophylaxis | Not included | Unrelated outcome |
|  | Practice and outcomes of neonatal resuscitation for newborns with birth asphyxia at Kakamega County General Hospital, Kenya: a direct observation study | Not included | Unrelated study |
|  | Hepatitis B virus infection among medical waste handlers in Addis Ababa, Ethiopia | Not included | Quality criteria |
|  | Information seeking behavior on hepatitis B virus, and its associated factors among pregnant women at teaching and specialized hospitals, Northwest Ethiopia: A cross-sectional study | Not included | Unrelated study |
|  | Prevalence and risk factors of hepatic steatosis and its impact on liver injury in Chinese patients with chronic hepatitis B infection | Not included | Outcome not measured clearly |
|  | Compliance and noncompliance of Universal Precautions among different groups of health care workers using the construct of the Health Belief Model: Implications for curriculum decision-making | Not included | Unrelated study |
|  | Hepatitis B virus serosurvey and awareness of mother-to-child transmission among pregnant women in Shenyang, China: An observational study | Not included | Study done in other setting |
|  | The hepatitis B core antibody positive/hepatitis B surface antigen negative pattern is associated with the increased risk of intracranial atherosclerotic stenosis | Not included | Unrelated outcome |
|  | Prevalence and factors associated with adverse drug reactions among heart failure patients hospitalized at Mbarara Regional Referral Hospital, Uganda | Not included | Unrelated study |
|  | High seroprevalence and associated risk factors for hepatitis B virus infection among pregnant women living with HIV in Mtwara region, Tanzania | Not included | Study done in other population |
|  | Acceptability and adherence to Isoniazid preventive therapy in HIV-infected patients clinically screened for latent tuberculosis in Dar es Salaam, Tanzania | Not included | Unrelated study |
|  | Seroprevalence of hepatitis B virus infection and associated factors among healthcare workers in northern Tanzania | Not included | Study done in other population |
|  | Seroprevalence of hepatitis B virus infection and associated factors among healthcare workers in northern Tanzania | Included |  |
|  | Multi-Omic Data Integration Allows Baseline Immune Signatures to Predict Hepatitis B Vaccine Response in a Small Cohort | Not included | Unrelated outcome |
|  | Alcohol-Related Liver Disease Is Rarely Detected at Early Stages Compared With Liver Diseases of Other Etiologies Worldwide | Not included | Unrelated outcome |
|  | Occupational safety and health in Spain | Not included | Study done in other setting |
|  | Infectious Diseases in Sub-Saharan Immigrants to Spain | Not included | Study done in other setting |
|  | Evaluating the appropriateness of chemotherapy in a low-resource cancer center in sub-Saharan Africa | Not included | Unrelated outcome |
|  | Presentation, patterns of care, and outcomes of patients with prostate cancer in sub-Saharan Africa: A population-based registry study | Not included | Unrelated study |
|  | Feasibility of the modified sequential organ function assessment score in a resource-constrained setting: a prospective observational study | Not included | Unrelated study |
|  | Prevalence and risk factors associated with HIV/hepatitis B and HIV/hepatitis C co-infections among people who inject drugs in Mozambique | Not included | Outcome not measured clearly |
|  | A prospective longitudinal study of psychosocial variables associated with the incidence of cancer among Seventh-day Adventists | Not included | Unrelated study |
|  | Anti-TB drug concentrations and drug-associated toxicities among TB/HIV-coinfected patients | Not included | Unrelated study |
|  | Occupational hazards | Not included | It is not study |
|  | Estimations of worldwide prevalence of chronic hepatitis B virus infection: a systematic review of data published between 1965 and 2013 | Not included | It is not primary study |
|  | Long-term follow-up of study participants from prophylactic HIV vaccine clinical trials in Africa | Not included | Unrelated study |
|  | The Swiss STAR trial - an evaluation of target groups for sexually transmitted infection screening in the sub-sample of men | Not included | Unrelated study |
|  | Using health-system-wide data to understand hepatitis B virus prophylaxis and reactivation outcomes in patients receiving rituximab | Not included | Unrelated outcome |
|  | Poor clinical and virological outcome of nucleos(t)ide analogue monotherapy in HBV/HDV co-infected patients | Not included | Unrelated outcome |
|  | A Study to Assess the Effectiveness of Structured Teaching Programme on Knowledge Regarding Universal Precautions and the Prevention of Blood Borne Infections Among the Final Year B. Sc. Nursing Students of Selected Nursing Colleges at Hassan, Karnataka | Not included | Unrelated outcome |
|  | Impact of changing societal trends on the spread of infections in American and Canadian homes | Not included | Unrelated outcome |
|  | The seroprevalence of the hepatitis B virus in Italian medical students after 3 decades since the introduction of universal vaccination | Not included | Study done in other setting |
|  | Correlates of infection and molecular characterization of blood-borne HIV, HCV, and HBV infections in HIV-1 infected inmates in Italy: An observational cross-sectional study | Not included | Study done in other setting |
|  | Prevalence and risk factors associated with hepatitis B and C in Nawabshah, Sindh, Pakistan | Not included | Study done in other setting |
|  | Percutaneous exposures among health care workers in a Greek tertiary hospital | Not included | Study done in other setting |
|  | Impact of Insulin Resistance on Therapeutic Response to Oral Treatment of Chronic Hepatitis C Virus Infection | Not included | Unrelated outcome |
|  | Antiretroviral prophylaxis of health care workers at two urban medical centers | Not included | Unrelated outcome |
|  | Patterns of antibiotic use, pathogens, and prediction of mortality in hospitalized neonates and young infants with sepsis: A global neonatal sepsis observational cohort study (NeoOBS) | Not included | Unrelated outcome |
|  | Guidelines for the prevention of invasive mould diseases caused by filamentous fungi by the Spanish Society of Infectious Diseases and Clinical Microbiology (SEIMC) | Not included | It is not study |
|  | Heterogeneity in neurocognitive change trajectories among people with HIV starting antiretroviral therapy in Rakai, Uganda | Not included | Unrelated outcome |
|  | Early reduced liver graft survival in hepatitis C recipients identified by two combined genetic markers | Not included | Unrelated outcome |
|  | Early reduced liver graft survival in hepatitis C recipients identified by two combined genetic markers | Not included | Unrelated outcome |
|  | Seroprevalence of Hepatitis B Virus and Associated Factors Among Pregnant Women Attending Antenatal Care in Public Health Facilities in Jigjiga Town, Eastern Ethiopia | Not included | Study done in other population |
|  | Career risk of hepatitis C virus infection among US emergency medical and public safety workers | Not included | Unrelated outcome |
|  | Prevalence, risk factors, and outcomes for occult hepatitis B virus infection among HIV-infected patients | Not included | Study done in other population |
|  | Reactive Blood Donor Notification; Their Responses And Perceptions: Experience From Southern Pakistan | Not included | Unrelated outcome |
|  | Steatosis in chronic hepatitis B: prevalence and correlation with biochemical, histologic, viral, and metabolic parameters | Not included | Unrelated outcome |
|  | Prevalence of hepatitis B virus, hepatitis C virus, and HIV infection among patients with newly diagnosed cancer from academic and community oncology practices | Not included | Study done in other population |
|  | Nationwide retrospective study of hepatitis B virological response and liver stiffness improvement in 465 patients on nucleos(t)ide analogue | Not included | Unrelated outcome |
|  | Hepatitis C virus infection in the Middle East and North Africa “MENA” region: injecting drug users (IDUs) is an under-investigated population | Not included | Unrelated outcome |
|  | Novel point-of-care cytokine biomarker lateral flow test for the screening for sexually transmitted infections and bacterial vaginosis: study protocol of a multicentre multidisciplinary prospective observational clinical study to evaluate the performance and feasibility of the Genital InFlammation Test (GIFT) | Not included | Unrelated outcome |
|  | Existing gaps and missed opportunities in delivering quality nutrition services in primary healthcare: a descriptive analysis of patient experience and provider competence in 11 low-income and middle-income countries | Not included | Unrelated outcome |
|  | Capacity and quality of maternal and child health services delivery at the subnational primary healthcare level in relation to intermediate health outputs: a cross-sectional study of 12 low-income and middle-income countries | Not included | Unrelated outcome |
|  | Waste Management With Special emphasis on occupational health and safety at selected healthcare establishments | Not included | Unrelated outcome |
|  | Factors affecting the serological testing of cadaveric donor cornea | Not included | Unrelated study |
|  | Identifying occupational hazards among healthcare workers in Australia and Bhutan | Not included | Unrelated outcome plus it is done in other setting |
|  | Chapter 30 - Occupational Health of Laboratory Animal Workers | Not included | It is not study |
|  | Prevalence of hepatitis B and C viral infections in Pakistan: findings of a national survey appealing for effective prevention and control measures | Not included | Study done in other setting |
|  | Immunizations and oral health care providers | Not included | Unrelated outcome |
|  | A positive-feedback loop between HBx and ALKBH5 promotes hepatocellular carcinogenesis | Not included | Unrelated outcome |
|  | Serum Liver Fibrosis Markers in the Prognosis of Liver Cirrhosis: A Prospective Observational Study | Not included | Unrelated outcome |
|  | Expansion of Stem Cell-Like CD4(+) Memory T Cells during Acute HIV-1 Infection Is Linked to Rapid Disease Progression | Not included | Unrelated outcome |
|  | Cancer risk by social class and occupation: a survey of 109,000 cancer cases among Finns of working age | Not included | Unrelated outcome |
|  | Improved Antibody Response to Three Additional Hepatitis B Vaccine Doses Following Primary Vaccination Failure in Patients with Inflammatory Bowel Disease | Not included | Unrelated outcome |
|  | Sleep and antibody response to hepatitis B vaccination | Not included | Unrelated outcome |
|  | Emerging and Re-emerging Pathogens and Diseases, and Health Consequences of a Changing Climate | Not included | It is not study |
|  | Nurses and AIDS care: Occupational risk perception and the social construction of HIV | Not included | Unrelated outcome |
|  | Liver steatosis in children with chronic hepatitis B and C: Prevalence, predictors, and impact on disease progression | Not included | It is not study |
|  | Prevalence of hepatitis B virus (HBV) infection among Makerere University medical students | Not included | Study done in other population |
|  | Suppl-1, M3: epidemiology of hepatitis B virus (HBV) and hepatitis C virus (HCV) related hepatocellular carcinoma | Not included | Unrelated outcome |
|  | Hepatitis B virus infection and vaccine-induced immunity in Madrid (Spain) | Not included | Study done in other setting |
|  | Costs of needlestick injuries and subsequent hepatitis and HIV infection | Not included | Unrelated outcome |
|  | To Study the Incidence, Predictive Factors and Clinical Outcome of Spontaneous Bacterial Peritonitis in Patients of Cirrhosis with Ascites | Not included | Unrelated outcome |
|  | Delayed-type hypersensitivity and hepatitis B vaccine responses, in vivo markers of cellular and humoral immune function, and the risk of AIDS or death | Not included | Unrelated outcome |
|  | Sharps injuries among hospital workers in Massachusetts, 2007 | Not included | Outcome not measured clearly |
|  | Clinical features of HBsAg seroclearance in hepatitis B virus carriers in South Korea: A retrospective longitudinal study | Not included | Study done in other setting |
|  | Global patterns of hepatocellular carcinoma management from diagnosis to death: the BRIDGE Study | Not included | Unrelated outcome |
|  | Assessing significant fibrosis using imaging-based elastography in chronic hepatitis B patients: Pilot study | Not included | Outcome not measured clearly |
|  | Liver Transplantation from Brain-Dead Donors with Hepatitis B or C in South Korea: A 2014-2020 Korean Organ Transplantation Registry Data Analysis | Not included | Outcome not measured clearly |
|  | Ten-Year Changes in the Hepatitis B Prevalence in the Birth Cohorts in Korea: Results From Nationally Representative Cross-Sectional Surveys | Not included | Unrelated outcome |
|  | A Study to Assess the Knowledge and Attitude Regarding Occupational Exposure and Post Exposure Prophylaxis (PEP) for Hiv Among Student Nurses of Selected Nursing Institutes of Hubballi with a View to Develop an Information Guide Sheet | Not included | Outcome not measured clearly |
|  | Observational and Genetic Associations of Body Mass Index and Hepatobiliary Diseases in a Relatively Lean Chinese Population | Not included | Unrelated outcome |
|  | Prevalence, risk factors and virological profile of chronic hepatitis B virus infection in pregnant women in India | Not included | Study done in other setting |
|  | A novel system for predicting liver histopathology in patients with chronic hepatitis B | Not included | Outcome not measured clearly |
|  | Hepatitis B immunization data of patients living with HIV/AIDS: a multi-centre study | Not included | Unrelated outcome |
|  | Human immunodeficiency virus infection predictors and genetic diversity of hepatitis B virus and hepatitis C virus co-infections among drug users in three major Kenyan cities | Not included | Unrelated outcome |
|  | Global epidemiology of hepatitis B virus infection: new estimates of age-specific HBsAg seroprevalence and endemicity | Not included | Outcome not measured clearly |
|  | Exposure time to hepatitis B virus and associated risk factors among children in Edirne, Turkey | Not included | Study done in other setting |
|  | Hepatitis B virus infection among illegal drug users in Enugu State, Nigeria: prevalence, immune status, and related risk factors | Not included | Study done in other population |
|  | Staff Nurse Education on Best Practices for Preventing Blood-Borne Pathogen Exposures | Not included | Unrelated outcome |
|  | Evaluating vertical transmission of sexually transmitted infections to newborns | Not included | Unrelated outcome |
|  | A systematic review and meta-analysis of the prevalence of hepatitis B virus infection among pregnant women in Nigeria | Not included | It is not a primary study |
|  | Knowledge, attitude, and risk factors of hepatitis B among waste scavengers in Lagos, Nigeria | Not included | Unrelated outcome |
|  | A multi-centre cross-sectional study on hepatitis B vaccination coverage and associated factors among personnel working in health facilities in Kumasi, Ghana | Not included | Unrelated outcome |
|  | The impact of HIV on hepatocellular cancer survival in Nigeria | Not included | Unrelated outcome |
|  | Factors Influencing Hospital Cleaners’ Knowledge and Practices toward Hepatitis B prevention in Northern Province of Rwanda | Not included | Unrelated outcome |
|  | A proposed predictive model for advanced fibrosis in patients with chronic hepatitis B and its validation | Not included | Unrelated outcome |
|  | Hepatitis C prevalence and associated risk factors among individuals who are homeless and diagnosed with mental illness: At Home/Chez Soi Study, Vancouver, BC | Not included | Unrelated outcome |
|  | Hepatitis A vaccines | Not included | It is not study |
|  | Prevalence of hepatitis B virus infection in Nigeria, 2000-2013: A systematic review and meta-analysis | Not included | The study is not primary study |
|  | Seroprevalence of hepatitis B virus among antenatal clinic attendees in Gamawa Local Government Area, Bauchi State, Nigeria | Not included | Study done in other population |
|  | Syphilis and HIV prevalence and associated factors to their co-infection, hepatitis B and hepatitis C viruses prevalence among female sex workers in Rwanda | Not included | Outcome not measured clearly |
|  | Prevalence of Hepatitis B Virus (HBV) surface antigen and HBVassociated hepatocellular carcinoma in Kenyans of various ages | Not included | Study done in general population |
|  | A nationwide cross-sectional review of in-hospital hepatitis B virus testing and disease burden estimation in Ghana, 2016 - 2021 | Not included | It is not primary study |
|  | Studies on prevalence and risk factors for Hepatitis B Surface Antigen among secondary school students in north-central, Nigeria | Not included | Stud done in other population |
|  | Multidimensional Analysis of the Mother-to-child Transmission Risk Factors in Chronic Hepatitis B Virus Infection in Pregnant Women in Vietnam | Not included | Study done in other setting |
|  | Healthcare resource utilization and costs by disease severity in an insured national sample of US patients with chronic hepatitis B | Not included | Unrelated outcome |
|  | Advancing Age and Comorbidity in a US Insured Population-Based Cohort of Patients With Chronic Hepatitis B | Not included | Unrelated outcome |
|  | Hepatitis B‐related hepatocellular carcinoma: epidemiological characteristics and disease burden | Not included | Stud done in other population |
|  | An enormous hepatitis B virus‐related liver disease burden projected in Vietnam by 2025 | Not included | Study done in other setting |
|  | Hepatitis B in healthcare personnel: an update on the global landscape | Not included | Outcome not measured clearly |
|  | Prevalence, infectivity and correlates of hepatitis B virus infection among pregnant women in a rural district of the Far North Region of Cameroon | Not included | Study done in other population |
|  | Assessing risk behaviors and prevalence of sexually transmitted and blood-borne infections among female crack cocaine users in Salvador-Bahia, Brazil | Not included | Study done in other setting |
|  | Standard Precautions among HealthCare Workers in a Tertiary Health Facility in Enugu Metropolis, South-East Nigeria | Not included | Unrelated outcome |
|  | HIV viraemia during hepatitis B vaccination shortens the duration of protective antibody levels | Not included | Unrelated outcome |
|  | Viral load is a significant prognostic factor for hepatitis B virus‐associated hepatocellular carcinoma | Not included | Unrelated outcome |
|  | Dual positivity of hepatitis B surface antigen and anti-hepatitis C virus antibody and associated factors among apparently healthy patients of Ekiti State, Nigeria | Not included | Unrelated outcome |
|  | Hepatitis C virus infection and its associated factors among prisoners in a Nigerian prison | Not included | Unrelated outcome |
|  | Early cranial ultrasound findings among infants with neonatal encephalopathy in Uganda: an observational study | Not included | Unrelated outcome |
|  | Sero-prevalence of hepatitis B virus and associated factors among pregnant women in Gambella hospital, South Western Ethiopia: facility based cross-sectional study | Not included | Study done in other population |
|  | Clinical characteristics and current management of hepatitis B and C in China | Not included | Study done in other setting |
|  | No contribution of lifestyle and environmental exposures to gender discrepancy of liver disease severity in chronic hepatitis b infection: Observations from the Haimen City cohort | Not included | Unrelated outcome |
|  | Sero-Prevalence of Hepatitis B Virus Infection and Associated Factors Among Pregnant Women Attending Antenatal Care Services in Gedeo Zone, Southern Ethiopia | Not included | Study done in other population |
|  | Effects of long-term antiretroviral therapy in reproductive-age women in sub-Saharan Africa (the PEPFAR PROMOTE study): a multi-country observational cohort study | Not included | Unrelated study |
|  | Prevalence, genotype distribution and mutations of hepatitis B virus and the associated risk factors among pregnant women residing in the northern shores of Persian Gulf, Iran | Not included | Study done in other setting |
|  | Global Estimates on Biological Risks at Work | Not included | It is not primary study |
|  | Telbivudine treatment of hepatitis B virus-infected pregnant women at different gestational stages for the prevention of mother-to-child transmission: Outcomes of telbivudine treatment during pregnancy | Not included | Unrelated outcome |
|  | Occult Hepatitis B Virus Infection in Maintenance Hemodialysis Patients: Prevalence and Mutations in "a" Determinant | Not included | Study done in other population |
|  | Prevalence of hepatitis B virus infection in Shenzhen, China, 2015–2018 | Not included | Study done in other setting |
|  | Identifying, preventing and controlling needle-stick injuries in Indonesia | Not included | Unrelated outcome |
|  | Splash of body fluids among healthcare support staff in Ghana: a cross-sectional study | Not included | Unrelated outcome |
|  | Physiological and psychosocial stressors among hemodialysis patients in the Buea Regional Hospital, Cameroon | Not included | Unrelated outcome |
|  | Safety practice and associated factors among waste handlers in Governmental Hospitals in Addis Ababa, Ethiopia | Not included | Unrelated outcome |
|  | Hepatitis B virus infection and associated risk factors among medical students in eastern Ethiopia | Not included | Study done in other population |
|  | Risk factors associated with Hepatitis B virus infection among pregnant women attending public hospitals in Addis Ababa, Ethiopia | Not included | Study done in other population |
|  | Clinicopathological analysis of patients with dual malignancies: A retrospective study | Not included | Study done in other population |
|  | Prevalence and the associated factors of hepatitis B and hepatitis C viral infections among HIV-positive individuals in same-day antiretroviral therapy initiation program in Bangkok, Thailand | Not included | Study done in other setting |
|  | Prevalence of liver steatosis in patients with chronic hepatitis B: a study of associated factors and of relationship with fibrosis | Not included | Unrelated outcome |
|  | Hepatitis B, hepatitis C, and mortality among HIV-positive individuals | Not included | Outcome not measured clearly |
|  | Nephrotoxicity caused by oral antiviral agents in patients with chronic hepatitis B treated in a hospital for tropical diseases in Thailand | Not included | Study done in other setting |
|  | Sexual behaviour and practices among adolescent blood donors in Harare and Masvingo provinces, Zimbabwe | Not included | Outcome not measured clearly |
|  | Thermal disinfection in hemodialysis using the A0 concept as dispenser | Not included | Unrelated study |
|  | Foodborne Diseases: Overview of Biological Hazards and Foodborne Diseases | Not included | It is not primary study |
|  | Guidelines for Preventing Infectious Complications among Hematopoietic Cell Transplantation Recipients: A Global Perspective | Not included | It is not study |
|  | Barriers to hepatitis B vaccine coverage among healthcare workers in the Republic of Georgia: An international perspective | Not included | Unrelated study |
|  | The impact of economic and social factors on the prevalence of hepatitis B in Turkey | Not included | Study done in other setting |
|  | Proliferative lupus nephritis in the absence of overt systemic lupus erythematosus: A historical study of 12 adult patients | Not included | Unrelated study |
|  | Seroprevalence of hepatitis B and C virus infections and risk factors in Turkey: a fieldwork TURHEP study | Not included | Study done in other setting |
|  | Chronic liver disease detection and quantification | Not included | Outcome not measured clearly |
|  | Inequalities in the use of secondary prevention of cardiovascular disease by socioeconomic status: evidence from the PURE observational study | Not included | Outcome not measured clearly |
|  | Distinct forms of migration and mobility are differentially associated with HIV treatment adherence | Not included | Outcome not related |
|  | Iron Status and Associated Malaria Risk Among African Children | Not included | Outcome not related |
|  | Comparison of viral hepatitis-associated hepatocellular carcinoma due to HBV and HCV - cohort from liver clinics in Pakistan | Not included | Outcome not related |
|  | Factors associated with anti-hepatitis A virus immunoglobulin G seropositivity among Korean workers: a cross-sectional study | Not included | Outcome not related |
|  | Uptake of long acting reversible contraception following integrated couples HIV and fertility goal-based family planning counselling in Catholic and non-Catholic, urban and rural government health centers in Kigali, Rwanda | Not included | Outcome not related |
|  | Infection control practices in clinical laboratories in Pakistan | Not included | Study done in other setting |
|  | Uptake of long acting reversible contraception following integrated couples HIV and fertility goal-based family planning counselling in Catholic and non-Catholic, urban and rural government health centers in Kigali, Rwanda | Not included | Outcome not related |
|  | Prevalence and Factors Associated with Percutaneous Injuries and Splash Exposures among Health-Care Workers in Rift Valley Provincial and War Memorial Hospitals, Kenya | Not included | Outcome not related |
|  | Prevalence of hepatitis B virus infection in the Gezira state of central Sudan | Not included | Outcome not related |
|  | Abdominal surgical site infections: a prospective study of determinant factors in Harare, Zimbabwe | Not included | Outcome not related |
|  | Innate Immune Responses in Viral Hepatitis: the role of Kupffer cells and liver-derived monocytes in shaping intrahepatic immunity in mice using the LCMV infection model | Not included | Outcome not related |
|  | Post exposure prophylaxis following occupational exposure to HIV: a survey of health care workers in Mbeya, Tanzania, 2009-2010 | Not included | Outcome not related |
|  | Value of early change of serum C reactive protein combined to modified Alvarado score in the diagnosis of acute appendicitis | Not included | Outcome not related |
|  | Needle Stick and Sharps Injuries among Healthcare Workers in an Oncology Setting: A Retrospective Seven-Year Study | Not included | Outcome not related |
|  | Clinical presentation of pregnant women in isolation units for Ebola virus disease in Sierra Leone, 2014 | Not included | Outcome not related |
|  | Paenibacillus spp infection among infants with postinfectious hydrocephalus in Uganda: an observational case-control study | Not included | Unrelated study |
|  | Medication exposure during pregnancy: a pilot pharmacovigilance system using health and demographic surveillance platform | Not included | Unrelated study |
|  | Safety of artemether-lumefantrine exposure in first trimester of pregnancy: an observational cohort | Not included | Unrelated study |
|  | The independent effect of living in malaria hotspots on future malaria infection: an observational study from Misungwi, Tanzania | Not included | Unrelated study |
|  | Trends of frequency, mortality and risk factors among patients admitted with stroke from 2017 to 2019 to the medical ward at Kilimanjaro Christian Medical Centre hospital: a retrospective observational study | Not included | Outcome not measured clearly |
|  | Case-control study of risk factors for avian influenza A (H5N1) disease, Hong Kong, 1997 | Not included | Unrelated study |
|  | Prevalence of syphilis, human immunodeficiency virus, hepatitis B virus, and human T-lymphotropic virus infections and coinfections during prenatal screening in an urban Northeastern Brazilian population | Not included | Outcome not measured clearly |
|  | Plasmodium falciparum resistance and malaria presentation in children at Dongola specialist hospital: A prospective cohort study | Not included | Unrelated study |
|  | Innate Immune Responses in Viral Hepatitis: the role of Kupffer cells and liver-derived monocytes in shaping intrahepatic immunity in mice using the LCMV infection model | Not included | Unrelated outcome |
|  | Maternal hookworm modifies risk factors for childhood eczema: results from a birth cohort in Uganda | Not included | Unrelated outcome |
|  | Clinical presentation of pregnant women in isolation units for Ebola virus disease in Sierra Leone, 2014 | Not included | Unrelated outcome |
|  | Post exposure prophylaxis following occupational exposure to HIV: a survey of health care workers in Mbeya, Tanzania, 2009-2010 | Not included | Unrelated outcome |
|  | Development of a Web Application based on Machine Learning for screening esophageal varices in cirrhosis | Not included | Unrelated outcome |
|  | Value of early change of serum C reactive protein combined to modified Alvarado score in the diagnosis of acute appendicitis | Not included | Unrelated outcome |
|  | Needlestick and sharps injuries among healthcare workers in an oncology setting: a retrospective 7-year cross-sectional study | Not included | Unrelated outcome |
|  | Needlestick and sharps injuries among healthcare workers in an oncology setting: a retrospective 7-year cross-sectional study | Not included | Unrelated outcome |
|  | Abdominal surgical site infections: a prospective study of determinant factors in Harare, Zimbabwe | Not included | Unrelated outcome |
|  | Prevalence of hepatitis B virus infection in the Gezira state of central Sudan | Not included | Unrelated outcome |
|  | Impact of the Safe Childbirth Checklist on health worker childbirth practices in Luapula province of Zambia: a pre-post study | Not included | Unrelated outcome |
|  | Prevalence and Factors Associated with Percutaneous Injuries and Splash Exposures among Health-Care Workers in Rift Valley Provincial and War Memorial Hospitals, Kenya | Not included | Unrelated outcome |
|  | Knowledge, attitudes, practices and prevalence of hepatitis B and C and hepatitis B vaccination coverage among public sector healthcare workers in Cambodia | Not included | Unrelated outcome |
|  | Natural History of Untreated HBeAg-Positive Chronic HBV Infection With Persistently Elevated HBV DNA but Normal Alanine Aminotransferase | Not included | Unrelated outcome |
|  | Pretransplant Hepatitis B Viral Infection Increases Risk of Death After Kidney Transplantation: A Multicenter Cohort Study in Korea | Not included | Study done in other setting |
|  | Adefovir- or Lamivudine-Induced Renal Tubular Dysfunction after Liver Transplantation | Not included | Unrelated outcome |
|  | Prevalence of hepatitis B virus and associated risk factors among adults patients at Dessie referral and Kemise general hospitals in northeastern Ethiopia | Not included | Study done in other population |
|  | Childhood vaccination coverage and regional differences in Swiss birth cohorts 2012-2021: Are we on track? | Not included | Unrelated outcome |
|  | The renal angina index accurately predicts low risk of developing severe acute kidney injury among children admitted to a low-resource pediatric intensive care unit | Not included | Unrelated outcome |
|  | Characteristics, complications, and gaps in evidence-based interventions in rheumatic heart disease: the Global Rheumatic Heart Disease Registry (the REMEDY study) | Not included | Unrelated outcome |
|  | Occupational stress and health among home health care workers | Not included | Unrelated outcome |
|  | The Safety and Immunogenicity of the mRNA-BNT162b2 SARS-CoV-2 Vaccine in Hemodialysis Patients | Not included | Unrelated outcome |
|  | Association of Combined Tobacco Smoking, Hormonal Contraceptive use and Status Matrimonial with Cervical Cancer Evolution in Tunisian Women | Not included | Unrelated outcome |
|  | Treatment effects of the differential first-line antiretroviral regimens among HIV/HBV coinfected patients in southwest China: an observational study | Not included | Unrelated outcome |
|  | Long-term survival and recurrence after curative resection for hepatocellular carcinoma in patients with chronic hepatitis C virus infection: a multicenter observational study from China | Not included | Unrelated outcome |
|  | Risk factors for combined hepatocellular-cholangiocarcinoma: a hospital-based case-control study | Not included | Unrelated outcome |
|  | Humoral immune responses to inactivated COVID-19 vaccine up to 1 year in children with chronic hepatitis B infection | Not included | Unrelated outcome |
|  | Validation of the Korean Stroop Test in Diagnosis of Minimal Hepatic Encephalopathy | Not included | Unrelated study |
|  | Seroprevalence and predictors of hepatitis B virus infection among pregnant women attending routine antenatal care in Arba Minch Hospital, South Ethiopia | Not included | Study done in other population |
|  | Conservation and variability of hepatitis B core at different chronic hepatitis stages | Not included | Unrelated outcome |
|  | Prevalence of Hepatitis B virus infection and its determinants among pregnant women in East Africa: Systematic review and Meta-analysis | Not included | It is not a primary study |
|  | HCC risk reduction with oral nucleos(t)ide analogues in patients with chronic hepatitis B: Not perfect, not good enough | Not included | Unrelated outcome |
|  | Occupational exposures to blood and body fluids (BBFS) among health care workers and medical students in University of Gondar Hospital, Northwest of Ethiopia | Not included | Outcome not measured clearly |
|  | Somatosensory Amplification, Anxiety, and Depression in Patients With Hepatitis B: Impact on Functionality | Not included | Unrelated outcome |
|  | Health behaviors of Korean adults with hepatitis B: Findings of the 2016 Korean National Health and Nutrition Examination Survey | Not included | Unrelated outcome |
|  | Prevalence of and risk factors for hepatitis C virus antibody among people who inject drugs in Cambodia: a national biological and behavioral survey | Not included | Study done in other population |
|  | An updated systematic review and meta-analysis of the prevalence of hepatitis B virus in Ethiopia | Not included | It is not a primary study |
|  | Management of Takotsubo cardiomyopathy in non-academic hospitals in France: The Observational French SyndromEs of TakoTsubo (OFSETT) study | Not included | Unrelated study |
|  | Clinical characteristics, prognosis, and surgical outcomes of patients with non-HBV and non-HCV related hepatocellular carcinoma: three-decade observational study | Not included | Unrelated outcome |
|  | Characteristics of female sexual dysfunctions and obstetric complications related to female genital mutilation in Omdurman maternity hospital, Sudan | Not included | Unrelated study |
|  | Prognostic value of immunoscore to identify mortality outcomes in adults with HBV-related primary hepatocellular carcinoma | Not included | Unrelated outcome |
|  | The association of adverse outcomes in the mother with disease progression in offspring in families with clusters of hepatitis B virus infection and unfavorable prognoses in Northwest China | Not included | Study done in other setting |
|  | Hospital acquired infections and infection prevention practices in teaching hospitals in the Amhara regional state, Ethiopia | Not included | Outcome not measured clearly |
|  | Sero-prevalence of hepatitis B virus infection and its risk factors among pregnant women attending antenatal clinic at Aminu Kano Teaching Hospital, Kano, Nigeria | Not included | Study done in other population |
|  | Relationship of Treg/Th17 balance with HBeAg change in HBeAg-positive chronic hepatitis B patients receiving telbivudine antiviral treatment: A longitudinal observational study | Not included | Unrelated outcome |
|  | Occupational exposures to blood and body fluids (BBFS) among health care workers and medical students in University of Gondar Hospital, Northwest of Ethiopia | Not included | Outcome not measured clearly |
|  | Telbivudine and adefovir dipivoxil combination therapy improves renal function in patients with chronic hepatitis B: A STROBE-compliant article | Not included | Outcome not measured clearly |
|  | Outcomes of Glucocorticoid Treatment in HBV Associated Acute-on-Chronic Liver Failure Patients: A Retrospective Observational Study | Not included | Study done in other population |
|  | Multimodal Ultrasound Model Based on the Left Gastric Vein in B-Viral Cirrhosis: Noninvasive Prediction of Esophageal Varices | Not included | Unrelated outcome |
|  | Multiple Primary Malignancies in Patients With Hepatocellular Carcinoma: A Largest Series With 26-Year Follow-Up | Not included | Unrelated outcome |
|  | Expression profiles of transcription factors for special CD4+ T-cell subsets in peripheral blood mononuclear cells from patients with hepatitis B virus infection | Not included | Unrelated outcome |
|  | Hepatitis B virus-associated hepatocellular carcinoma | Not included | Outcome not measured clearly |
|  | HBV pgRNA profiles in Chinese HIV/HBV coinfected patients under pre- and posttreatment: a multicentre observational cohort study | Not included | Study done in other setting |
|  | Population-Based Multicentric Survey of Hepatitis B Infection and Risk Factors in the North, South, and Southeast Regions of Brazil, 10-20 Years After the Beginning of Vaccination | Not included | Study done in general population |
|  | Analysis of serum hepatitis B virus RNA levels among HBsAg and HBsAb copositive patients and its correlation with HBV DNA | Not included | Study done in general population |
|  | Global Burden and Trends of Primary Liver Cancer Attributable to Comorbid Type 2 Diabetes Mellitus Among People Living with Hepatitis B: An Observational Trend Study from 1990 to 2019 | Not included | Study is not a primary study |
|  | Systemic immune-inflammation index predicts postoperative acute kidney injury in hepatocellular carcinoma patients after hepatectomy | Not included | Unrelated outcome |
|  | The imported infections among foreign travelers in China: an observational study | Not included | Study done in other setting |
|  | An updated systematic review and meta-analysis of the prevalence of hepatitis B virus in Ethiopia | Not included | Outcome not measured clearly |
|  | Hospital acquired infections and infection prevention practices in teaching hospitals in the Amhara regional state, Ethiopia | Not included | Outcome not measured clearly |
|  | 2022 International Consensus on Cardiopulmonary Resuscitation and Emergency Cardiovascular Care Science With Treatment Recommendations: Summary From the Basic Life Support; Advanced Life Support; Pediatric Life Support; Neonatal Life Support; Education, Implementation, and Teams; and First Aid Task Forces | Not included | Unrelated Study |
|  | Mercury Exposure and Poor Nutritional Status Reduce Response to Six Expanded Program on Immunization Vaccines in Children: An Observational Cohort Study of Communities Affected by Gold Mining in the Peruvian Amazon | Not included | Unrelated Study |
|  | Maternal exposure to carbon monoxide and fine particulate matter during pregnancy in an urban Tanzanian cohort | Not included | Unrelated Study |
|  | Sero-prevalence of hepatitis B virus infection and its risk factors among pregnant women attending antenatal clinic at Aminu Kano Teaching Hospital, Kano, Nigeria | Not included | Outcome not measured clearly |
|  | Hepatitis B virus-associated hepatocellular carcinoma | Not included | Outcome not measured clearly |
|  | Add-on pegylated interferon augments hepatitis B surface antigen clearance vs continuous nucleos(t)ide analog monotherapy in Chinese patients with chronic hepatitis B and hepatitis B surface antigen ≤ 1500 IU/mL: An observational study | Not included | Study done in other population |
|  | Pneumococcal and influenza vaccination coverage among at-risk adults: A 5-year French national observational study | Not included | Unrelated Study |
|  | A modified MELD model for Chinese pre-ACLF and ACLF patients and it reveals poor prognosis in pre-ACLF patients | Not included | Unrelated study |
|  | Analysis of serum hepatitis B virus RNA levels among HBsAg and HBsAb copositive patients and its correlation with HBV DNA | Not included | Outcome not measured clearly |
|  | Statins improve outcomes of nonsurgical curative treatments in hepatocellular carcinoma patients | Not included | Unrelated study |
|  | Genome-wide study of salivary microRNAs as potential noninvasive biomarkers for detection of nasopharyngeal carcinoma | Not included | Unrelated study |
|  | Comparison of the efficacy and safety of entecavir and tenofovir in nucleos(t)ide analogue-naive chronic hepatitis B patients with high viraemia: a retrospective cohort study | Not included | Outcome not measured clearly |
|  | Multicenter study of skin rashes and hepatotoxicity in antiretroviral-naïve HIV-positive patients receiving non-nucleoside reverse-transcriptase inhibitor plus nucleoside reverse-transcriptase inhibitors in Taiwan | Not included | Unrelated study |
|  | Methylation status of the stimulator of interferon genes promoter in patients with chronic hepatitis B | Not included | Unrelated outcome |
|  | Postnatal infection surveillance by telephone in Dar es Salaam, Tanzania: An observational cohort study | Not included | Outcome not measured clearly |
|  | MRI findings in people with epilepsy and nodding syndrome in an area endemic for onchocerciasis: an observational study | Not included | Unrelated study |
|  | Bacterial pathogenesis: a molecular approach | Not included | It is not study |
|  | Clinical features of treatment-naive patients with hepatitis B virus infection: A community-based survey from high- and intermediate-hepatitis B endemicity regions in Southeast China | Not included | Study done in other setting |
|  | Rituximab plus chemotherapy as first-line treatment in Chinese patients with diffuse large B-cell lymphoma in routine practice: a prospective, multicentre, non-interventional study | Not included | Unrelated study |
|  | Albumin-bilirubin and platelet-albumin-bilirubin grades for hepatitis B-associated hepatocellular carcinoma in Child-Pugh A patients treated with radical surgery: A retrospective observational study | Not included | Unrelated outcome |
|  | Hepatocellular carcinoma amongst aboriginal and torres strait islander peoples of Australia | Not included | Study done in other setting |
|  | Hepatitis B vaccination status and associated factors among undergraduate students of Makerere University College of Health Sciences | Not included | Study done in other population |
|  | Risk factors for underlying comorbidities and complications in patients with hepatitis B virus-related acute-on-chronic liver failure | Not included | Unrelated outcome |
|  | Psychological profiles of excluded living liver donor candidates: An observational study | Not included | Unrelated outcome |
|  | Prevalence of Hepatitis E Virus and Its Associated Outcomes among Pregnant Women in China | Not included | Study done in other population |
|  | Clinical cure induced by pegylated interferon α-2b in the advantaged population of chronic hepatitis B virus infection: a retrospective cohort study | Not included | Unrelated outcome |
|  | Prevalence and associated risk factors of Hepatitis B and Hepatitis C virus among volunteer blood donors in Arba Minch Blood Bank SNNPR, Ethiopia | Not included | Study done in other population |
|  | Genetic variation in FCER1A predicts peginterferon alfa-2a-induced hepatitis B surface antigen clearance in East Asian patients with chronic hepatitis B | Not included | Unrelated outcome |
|  | Cirrhosis and liver transplantation in patients co-infected with HIV and hepatitis B or C: an observational cohort study | Not included | Unrelated outcome |
|  | Mutations in pre-core and basic core promoter regions of hepatitis B virus in chronic hepatitis B patients | Not included | Unrelated outcome |
|  | Immune response pattern varies with the natural history of chronic hepatitis B | Not included | Unrelated outcome |
|  | Role of interleukin-21 and interleukin-21 receptor polymorphisms in the treatment of HBeAg-positive chronic hepatitis B patients with peginterferon | Not included | Unrelated outcome |
|  | The prevalence of hepatitis B virus infection in the United States in the era of vaccination | Not included | Study done in other setting |
|  | A Pilot Study of MicroRNAs Expression Profile in Serum and HBsAg Particles: Predictors of Therapeutic Vaccine Efficacy in Chronic Hepatitis B Patients | Not included | Study done in other population |
|  | An epidemiological survey of HBV infection and low-level HBsAg in military camps in eastern China | Not included | Study done in other setting |
|  | Epidemiology and etiology of diffuse large B-cell lymphoma | Not included | Unrelated study |
|  | Hepatitis B virus infection and related factors in hemodialysis patients in China–systematic review and meta-analysis | Not included | Study done in other setting and is not primary study |
|  | Prevalence of chronic obstructive pulmonary disease and associated risk factors in Uganda (FRESH AIR Uganda): a prospective cross-sectional observational study | Not included | Unrelated study |
|  | Stopping nucleos(t)ide analogue treatment in Caucasian hepatitis B patients after HBeAg seroconversion is associated with high relapse rates and fatal outcomes | Not included | Unrelated outcome |
|  | Caucasian Ethnicity, but Not Treatment Cessation is Associated with HBsAg Loss Following Nucleos(t)ide Analogue-Induced HBeAg Seroconversion | Not included | Unrelated outcome |
|  | Prevalence an d factors associated with hepatitis B susceptibility among men who sex with men on HIV pre-exposure prophylaxis in Northeastern Brazil: a cross-sectional study | Not included | Stud done in other population |
|  | The Swiss STAR trial - an evaluation of target groups for sexually transmitted infection screening in the sub-sample of women | Not included | Unrelated outcome |
|  | Poor adherence and low persistency rates for hepatocellular carcinoma surveillance in patients with chronic hepatitis B | Not included | Outcome not measured clearly |
|  | Predictors of Hepatitis B Surface Antigen Titers two decades after vaccination in a cohort of students and post-graduates of the Medical School at the University of Palermo, Italy | Not included | Study done in other setting |
|  | High prevalence of hepatitis B virus and hepatitis D virus in the western Brazilian Amazon | Not included | Study done in other setting |
|  | Hepatitis B in Rondônia (Western amazon region, Brazil): descriptive analysis and spatial distribution | Not included | Study done in other setting |
|  | “I am still suffering:” The dilemma of multiple recoveries in the lives of methadone maintenance patients | Not included | It is not study |
|  | Syphilis, human immunodeficiency virus, herpes genital and hepatitis B in a women's prison in Cochabamba, Bolivia: prevalence and risk factors | Not included | Study done in other setting |
|  | Switch from intravenous or intramuscular to subcutaneous hepatitis B immunoglobulin: effect on quality of life after liver transplantation | Not included | Unrelated outcome |
|  | The value of APGA score, fibrosis index for diagnosing liver fibrosis in patients with chronic hepatitis B | Not included | Unrelated outcome |
|  | Epidemiological patterns and risk factors associated with hepatitis B virus in Pakistani population | Not included | Study done in other setting |
|  | Protocol: Prospective observational study investigating the prevalence and clinical outcome of portopulmonary hypertension in Japanese patients with chronic liver disease | Not included | Study done in other setting |
|  | Chapter 26 - Hepatitis A Vaccines | Not included | It is not study |
|  | Workplace hazards faced by nursing assistants in the United States: A focused literature review | Not included | Study done in other setting and is not primary study |
|  | Genotype Matters in Patients with Acute-on-chronic Liver Failure Due to Reactivation of Chronic Hepatitis B | Not included | Unrelated outcome |
|  | Sex difference in the associations among risk factors with hepatitis B and C infections in a large Taiwanese population study | Not included | Outcome not measured clearly |
|  | Protective effect of an improved immunization practice of mother-to-infant transmission of hepatitis B virus and risk factors associated with immunoprophylaxis failure | Not included | Outcome not measured clearly |
|  | Prevalence and factors associated with hepatitis B immunization and infection among men who have sex with men in Beijing, China | Not included | Study done in other setting |
|  | Comparison of hepatitis B virus and hepatitis C virus prevalence and risk factors in a community-based study | Not included | Outcome not measured clearly |
|  | Hepatitis C Virus Infection Associated With an Increased Risk of Deep Vein Thrombosis: A Population-Based Cohort Study | Not included | Unrelated study |
|  | Low immediate postoperative platelet count is associated with hepatic insufficiency after hepatectomy | Not included | Unrelated outcome |
|  | Clinical characteristics and risk factors of COVID-19 patients with chronic hepatitis B: a multi-center retrospective cohort study | Not included | Outcome not measured clearly |
|  | A statistical analysis of the correlations among various types of clinical indexes for patients with chronic hepatitis B: A hospital-based study | Not included | Outcome not clearly measured |
|  | Combined use of murine double minute-2 promoter methylation and serum AFP improves diagnostic efficiency in hepatitis B virus-related hepatocellular carcinoma | Not included | Outcome not related |
|  | Abnormal IL-10 levels were related to alanine aminotransferase abnormalities during postpartum in HBeAg positive women with chronic hepatitis B | Not included | Study done in other population |
|  | Gamma-glutamyl transpeptidase to platelet ratio index is a good noninvasive biomarker for predicting liver fibrosis in Chinese chronic hepatitis B patients | Not included | Study done in other setting |
|  | Risk factors for some tropical diseases in an African country | Not included | Outcome not clearly measured |
|  | Epidemiology of hepatitis B virus infection in Bangladesh: prevalence among general population, risk groups and genotype distribution | Not included | Study done in other population |
|  | Nosocomial infections: current situation in a resuscitation-unit | Not included | Outcome not measured clearly |
|  | Hepatitis B virus infection | Not included | Study done in other population |
|  | Does Nucleos(t)ide Analogues Treatment Affect Renal Function in Chronic Hepatitis B Patients Who Have Already Decreased eGFR? A Longitudinal Study | Not included | Outcome not related |
|  | Seroepidemiology of the human herpesvirus 8 infection among people living with HIV in Taiwan, 2014-2018 | Not included | Study done in other setting |
|  | Sero-prevalence and risk factors for hepatitis B virus infection among the consumers of the alcoholic beverage, cheka in Konso zone, southwestern Ethiopia | Not included | Study done in other population |
|  | Predictive factors for percutaneous and mucocutaneous exposure among healthcare workers in a developing country | Not included | Unrelated study |
|  | Prevalence and predictors of hepatitis B virus coinfection in a United States cohort of hepatitis C virus‐infected patients | Not included | Study done in other setting |
|  | Superiority of tenofovir alafenamide fumarate over entecavir for serum HBsAg level reduction in patients with chronic HBV infection: A 144-week outcome study after switching of the nucleos(t)ide analog | Not included | Outcome not clearly measured |
|  | The prevalence of hepatitis B and C viral infections among pregnant women | Not included | Study done in other population |
|  | Prevalence and risk factors of hepatitis B virus transmission among children in Enugu, Nigeria | Not included | Study done in other population |
|  | Hepatitis B virus infections and associated factors among pregnant women attending antenatal care clinic at Deder Hospital, Eastern Ethiopia | Not included | Study done in other population |
|  | Cross-sectional study of chronic hepatitis B virus infection in Rwandan high-risk groups: unexpected findings on prevalence and its determinants | Not included | Study done in other population |
|  | Screening a nation for hepatitis C virus elimination: a cross-sectional study on prevalence of hepatitis C and associated risk factors in the Rwandan general population | Not included | Unrelated outcome |
|  | Role of quantitative hepatitis B surface antigen in predicting inactive carriers and HBsAg seroclearance in HBeAg-negative chronic hepatitis B patients | Not included | Unrelated outcome |
|  | The role of Bcl-2 in hepatocarcinogenesis: Effects of overexpression on murine liver tumor development and hepatocyte cell cycle progression | Not included | Unrelated outcome |
|  | CHAPTER 12 - Alcohol and Substance Abuse | Not included | It is not study |
|  | Five‐year conditional survival for patients with hepatocellular carcinoma in Queensland, Australia | Not included | Study done in other setting |
|  | Admitted AIDS-associated Kaposi sarcoma patients: Indications for admission and predictors of mortality | Not included | Unrelated outcome |
|  | Sustained viral response and relapse after discontinuation of oral antiviral drugs in HBeAg-positive patients with chronic hepatitis B infection | Not included | Study done in other population |
|  | An observational study to evaluate the safety and efficacy of telbivudine in adults with chronic hepatitis B | Not included | Unrelated outcome |
|  | Clinical outcome indicators in chronic hepatitis B and C: A primer for value-based medicine in hepatology | Not included | Outcome not measured clearly |
|  | Patterns and co-occurrence of risk factors for hepatocellular carcinoma in four Asian American communities: a cross-sectional study | Not included | Study dome in other setting |
|  | A Prospective Study Evaluating Changes in Histology, Clinical and Virologic Outcomes in HBV-HIV Co-infected Adults in North America | Not included | Study dome in other setting |
|  | Long-term persistency of hepatitis B immunity: an observational cross-sectional study on medical students and resident doctors | Not included | Study dome in other population |
|  | Hepatitis B virus infection and factors associated with its acquisition among adults in a Lake Victoria HIV hyperendemic fishing community in Kyotera district, Uganda: a cross-sectional observation | Not included | Study dome in other population |
|  | Hepatitis B and C virus infection among 1.2 million persons with access to care: factors associated with testing and infection prevalence | Not included | Study dome in other population |
|  | Blood and virus detection on barber clippers | Not included | Study dome in other population |
|  | A new approach to prevent, diagnose, and treat hepatitis B in Africa | Not included | It is not primary study |
|  | Infectious diseases prevalence, vaccination coverage, and diagnostic challenges in a population of internationally adopted children referred to a Tertiary Care Children's Hospital from 2009 to 2015 | Not included | Study dome in other population |
|  | Renal angina index in critically ill children as an applicable and reliable tool in the prediction of severe acute kidney injury: Two tertiary centers' prospective observational study from the Middle East | Not included | Unrelated outcome |
|  | Associations Between Hepatitis B Virus Infection and Risk of All Cancer Types | Not included | Unrelated outcome |
|  | Off-treatment virologic relapse and outcomes of re-treatment in chronic hepatitis B patients who achieved complete viral suppression with oral nucleos(t)ide analogs | Not included | Unrelated outcome |
|  | Factors associated with sexually transmitted infections in sugarcane cutters: subsidies to caring for | Not included | Study dome in other population |
|  | Factors associated with immunoprophylaxis failure against vertical transmission of hepatitis B virus | Not included | Unrelated outcome |
|  | Hospital personnel sero-protected against hepatitis B virus following an accelerated vaccination program | Not included | Study dome in other population |
|  | Predictors of liver disease progression in people living with HIV-HBV co-infection on antiretroviral therapy | Not included | Study dome in other population |
|  | High prevalence of hepatitis B-antibody loss and a case report of de novo hepatitis B virus infection in a child after living-donor liver transplantation | Not included | Study dome in other population |
|  | Sexually transmitted infections among patients attending a sexual assault centre: a cohort study from Oslo, Norway | Not included | Unrelated outcome |
|  | Occupational risk perception in home health care workers | Not included | Unrelated outcome |
|  | Post-vaccination anti-HBs testing among healthcare workers: More economical than post-exposure management for Hepatitis B | Not included | Unrelated outcome |
|  | Blood donors and the supply of blood and blood products | Not included | It is not primary study |
|  | Pro-Inflammatory Interleukin-18 is Associated with Hepatic Steatosis and Elevated Liver Enzymes in People with HIV Monoinfection | Not included | Unrelated outcome |
|  | Sharps Injuries in Ambulatory Care: A Clinical Staff Perspective | Not included | Outcome not measured clearly |
|  | Seroevidence for a high prevalence of subclinical infection with avian influenza A (H5N1) virus among workers in a live-poultry market in Indonesia | Not included | Unrelated study |
|  | Effective therapeutic options for elderly patients with hepatocellular carcinoma: A nationwide cohort stud | Not included | Unrelated outcome |
|  | Risk Factors for Renal Functional Decline in Chronic Hepatitis B Patients Receiving Oral Antiviral Agents | Not included | Unrelated outcome |
|  | Prevalence and risk factors of hepatitis B virus, hepatitis C virus, and human immunodeficiency virus infections among drug addicts in Bangladesh | Not included | Study dome in other setting |
|  | Seroprevalence of hepatitis B virus infection and associated factors among prison inmates in state of Mato Grosso do Sul, Brazil | Not included | Study dome in other setting |
|  | Residual risk of mother-to-child transmission of hepatitis B virus infection despite timely birth-dose vaccination in Cameroon (ANRS 12303): a single-centre, longitudinal observational study | Not included | Unrelated outcome |
|  | Dually Active HIV/HBV Antiretrovirals as Protection Against Incident Hepatitis B Infections: Potential for Prophylaxis | Not included | Unrelated outcome |
|  | Practice and outcomes of neonatal resuscitation for newborns with birth asphyxia at Kakamega County General Hospital, Kenya: a direct observation study | Not included | Unrelated study |
|  | Hepatitis B virus infection among medical waste handlers in Addis Ababa, Ethiopia | Not included | Quality criteria |
|  | Information seeking behavior on hepatitis B virus, and its associated factors among pregnant women at teaching and specialized hospitals, Northwest Ethiopia: A cross-sectional study | Not included | Unrelated study |
|  | Prevalence and risk factors of hepatic steatosis and its impact on liver injury in Chinese patients with chronic hepatitis B infection | Not included | Outcome not measured clearly |
|  | Compliance and noncompliance of Universal Precautions among different groups of health care workers using the construct of the Health Belief Model: Implications for curriculum decision-making | Not included | Unrelated study |
|  | Hepatitis B virus serosurvey and awareness of mother-to-child transmission among pregnant women in Shenyang, China: An observational study | Not included | Study done in other setting |
|  | The hepatitis B core antibody positive/hepatitis B surface antigen negative pattern is associated with the increased risk of intracranial atherosclerotic stenosis | Not included | Unrelated outcome |
|  | Prevalence and factors associated with adverse drug reactions among heart failure patients hospitalized at Mbarara Regional Referral Hospital, Uganda | Not included | Unrelated study |
|  | High seroprevalence and associated risk factors for hepatitis B virus infection among pregnant women living with HIV in Mtwara region, Tanzania | Not included | Study done in other population |
|  | Acceptability and adherence to Isoniazid preventive therapy in HIV-infected patients clinically screened for latent tuberculosis in Dar es Salaam, Tanzania | Not included | Unrelated study |
|  | Seroprevalence of hepatitis B virus infection and associated factors among healthcare workers in northern Tanzania | Not included | Study done in other population |
|  | Seroprevalence of hepatitis B virus infection and associated factors among healthcare workers in northern Tanzania | Included |  |
|  | Multi-Omic Data Integration Allows Baseline Immune Signatures to Predict Hepatitis B Vaccine Response in a Small Cohort | Not included | Unrelated outcome |
|  | Alcohol-Related Liver Disease Is Rarely Detected at Early Stages Compared With Liver Diseases of Other Etiologies Worldwide | Not included | Unrelated outcome |
|  | Occupational safety and health in Spain | Not included | Study done in other setting |
|  | Infectious Diseases in Sub-Saharan Immigrants to Spain | Not included | Study done in other setting |
|  | Evaluating the appropriateness of chemotherapy in a low-resource cancer center in sub-Saharan Africa | Not included | Unrelated outcome |
|  | Presentation, patterns of care, and outcomes of patients with prostate cancer in sub-Saharan Africa: A population-based registry study | Not included | Unrelated study |
|  | Feasibility of the modified sequential organ function assessment score in a resource-constrained setting: a prospective observational study | Not included | Unrelated study |
|  | Prevalence and risk factors associated with HIV/hepatitis B and HIV/hepatitis C co-infections among people who inject drugs in Mozambique | Not included | Outcome not measured clearly |
|  | A prospective longitudinal study of psychosocial variables associated with the incidence of cancer among Seventh-day Adventists | Not included | Unrelated study |
|  | Anti-TB drug concentrations and drug-associated toxicities among TB/HIV-coinfected patients | Not included | Unrelated study |
|  | Occupational hazards | Not included | It is not study |
|  | Estimations of worldwide prevalence of chronic hepatitis B virus infection: a systematic review of data published between 1965 and 2013 | Not included | It is not primary study |
|  | Long-term follow-up of study participants from prophylactic HIV vaccine clinical trials in Africa | Not included | Unrelated study |
|  | The Swiss STAR trial - an evaluation of target groups for sexually transmitted infection screening in the sub-sample of men | Not included | Unrelated study |
|  | Using health-system-wide data to understand hepatitis B virus prophylaxis and reactivation outcomes in patients receiving rituximab | Not included | Unrelated outcome |
|  | Poor clinical and virological outcome of nucleos(t)ide analogue monotherapy in HBV/HDV co-infected patients | Not included | Unrelated outcome |
|  | A Study to Assess the Effectiveness of Structured Teaching Programme on Knowledge Regarding Universal Precautions and the Prevention of Blood Borne Infections Among the Final Year B. Sc. Nursing Students of Selected Nursing Colleges at Hassan, Karnataka | Not included | Unrelated outcome |
|  | Impact of changing societal trends on the spread of infections in American and Canadian homes | Not included | Unrelated outcome |
|  | The seroprevalence of the hepatitis B virus in Italian medical students after 3 decades since the introduction of universal vaccination | Not included | Study done in other setting |
|  | Correlates of infection and molecular characterization of blood-borne HIV, HCV, and HBV infections in HIV-1 infected inmates in Italy: An observational cross-sectional study | Not included | Study done in other setting |
|  | Prevalence and risk factors associated with hepatitis B and C in Nawabshah, Sindh, Pakistan | Not included | Study done in other setting |
|  | Percutaneous exposures among health care workers in a Greek tertiary hospital | Not included | Study done in other setting |
|  | Impact of Insulin Resistance on Therapeutic Response to Oral Treatment of Chronic Hepatitis C Virus Infection | Not included | Unrelated outcome |
|  | Antiretroviral prophylaxis of health care workers at two urban medical centers | Not included | Unrelated outcome |
|  | Patterns of antibiotic use, pathogens, and prediction of mortality in hospitalized neonates and young infants with sepsis: A global neonatal sepsis observational cohort study (NeoOBS) | Not included | Unrelated outcome |
|  | Guidelines for the prevention of invasive mould diseases caused by filamentous fungi by the Spanish Society of Infectious Diseases and Clinical Microbiology (SEIMC) | Not included | It is not study |
|  | Heterogeneity in neurocognitive change trajectories among people with HIV starting antiretroviral therapy in Rakai, Uganda | Not included | Unrelated outcome |
|  | Early reduced liver graft survival in hepatitis C recipients identified by two combined genetic markers | Not included | Unrelated outcome |
|  | Early reduced liver graft survival in hepatitis C recipients identified by two combined genetic markers | Not included | Unrelated outcome |
|  | Seroprevalence of Hepatitis B Virus and Associated Factors Among Pregnant Women Attending Antenatal Care in Public Health Facilities in Jigjiga Town, Eastern Ethiopia | Not included | Study done in other population |
|  | Career risk of hepatitis C virus infection among US emergency medical and public safety workers | Not included | Unrelated outcome |
|  | Prevalence, risk factors, and outcomes for occult hepatitis B virus infection among HIV-infected patients | Not included | Study done in other population |
|  | Reactive Blood Donor Notification; Their Responses And Perceptions: Experience From Southern Pakistan | Not included | Unrelated outcome |
|  | Steatosis in chronic hepatitis B: prevalence and correlation with biochemical, histologic, viral, and metabolic parameters | Not included | Unrelated outcome |
|  | Prevalence of hepatitis B virus, hepatitis C virus, and HIV infection among patients with newly diagnosed cancer from academic and community oncology practices | Not included | Study done in other population |
|  | Nationwide retrospective study of hepatitis B virological response and liver stiffness improvement in 465 patients on nucleos(t)ide analogue | Not included | Unrelated outcome |
|  | Hepatitis C virus infection in the Middle East and North Africa “MENA” region: injecting drug users (IDUs) is an under-investigated population | Not included | Unrelated outcome |
|  | Novel point-of-care cytokine biomarker lateral flow test for the screening for sexually transmitted infections and bacterial vaginosis: study protocol of a multicentre multidisciplinary prospective observational clinical study to evaluate the performance and feasibility of the Genital InFlammation Test (GIFT) | Not included | Unrelated outcome |
|  | Existing gaps and missed opportunities in delivering quality nutrition services in primary healthcare: a descriptive analysis of patient experience and provider competence in 11 low-income and middle-income countries | Not included | Unrelated outcome |
|  | Capacity and quality of maternal and child health services delivery at the subnational primary healthcare level in relation to intermediate health outputs: a cross-sectional study of 12 low-income and middle-income countries | Not included | Unrelated outcome |
|  | Waste Management With Special emphasis on occupational health and safety at selected healthcare establishments | Not included | Unrelated outcome |
|  | Factors affecting the serological testing of cadaveric donor cornea | Not included | Unrelated study |
|  | Identifying occupational hazards among healthcare workers in Australia and Bhutan | Not included | Unrelated outcome plus it is done in other setting |
|  | Chapter 30 - Occupational Health of Laboratory Animal Workers | Not included | It is not study |
|  | Prevalence of hepatitis B and C viral infections in Pakistan: findings of a national survey appealing for effective prevention and control measures | Not included | Study done in other setting |
|  | Immunizations and oral health care providers | Not included | Unrelated outcome |
|  | A positive-feedback loop between HBx and ALKBH5 promotes hepatocellular carcinogenesis | Not included | Unrelated outcome |
|  | Serum Liver Fibrosis Markers in the Prognosis of Liver Cirrhosis: A Prospective Observational Study | Not included | Unrelated outcome |
|  | Expansion of Stem Cell-Like CD4(+) Memory T Cells during Acute HIV-1 Infection Is Linked to Rapid Disease Progression | Not included | Unrelated outcome |
|  | Cancer risk by social class and occupation: a survey of 109,000 cancer cases among Finns of working age | Not included | Unrelated outcome |
|  | Improved Antibody Response to Three Additional Hepatitis B Vaccine Doses Following Primary Vaccination Failure in Patients with Inflammatory Bowel Disease | Not included | Unrelated outcome |
|  | Sleep and antibody response to hepatitis B vaccination | Not included | Unrelated outcome |
|  | Emerging and Re-emerging Pathogens and Diseases, and Health Consequences of a Changing Climate | Not included | It is not study |
|  | Nurses and AIDS care: Occupational risk perception and the social construction of HIV | Not included | Unrelated outcome |
|  | Liver steatosis in children with chronic hepatitis B and C: Prevalence, predictors, and impact on disease progression | Not included | It is not study |
|  | Prevalence of hepatitis B virus (HBV) infection among Makerere University medical students | Not included | Study done in other population |
|  | Suppl-1, M3: epidemiology of hepatitis B virus (HBV) and hepatitis C virus (HCV) related hepatocellular carcinoma | Not included | Unrelated outcome |
|  | Hepatitis B virus infection and vaccine-induced immunity in Madrid (Spain) | Not included | Study done in other setting |
|  | Costs of needlestick injuries and subsequent hepatitis and HIV infection | Not included | Unrelated outcome |
|  | To Study the Incidence, Predictive Factors and Clinical Outcome of Spontaneous Bacterial Peritonitis in Patients of Cirrhosis with Ascites | Not included | Unrelated outcome |
|  | Delayed-type hypersensitivity and hepatitis B vaccine responses, in vivo markers of cellular and humoral immune function, and the risk of AIDS or death | Not included | Unrelated outcome |
|  | Sharps injuries among hospital workers in Massachusetts, 2007 | Not included | Outcome not measured clearly |
|  | Clinical features of HBsAg seroclearance in hepatitis B virus carriers in South Korea: A retrospective longitudinal study | Not included | Study done in other setting |
|  | Global patterns of hepatocellular carcinoma management from diagnosis to death: the BRIDGE Study | Not included | Unrelated outcome |
|  | Assessing significant fibrosis using imaging-based elastography in chronic hepatitis B patients: Pilot study | Not included | Outcome not measured clearly |
|  | Liver Transplantation from Brain-Dead Donors with Hepatitis B or C in South Korea: A 2014-2020 Korean Organ Transplantation Registry Data Analysis | Not included | Outcome not measured clearly |
|  | Ten-Year Changes in the Hepatitis B Prevalence in the Birth Cohorts in Korea: Results From Nationally Representative Cross-Sectional Surveys | Not included | Unrelated outcome |
|  | A Study to Assess the Knowledge and Attitude Regarding Occupational Exposure and Post Exposure Prophylaxis (PEP) for Hiv Among Student Nurses of Selected Nursing Institutes of Hubballi with a View to Develop an Information Guide Sheet | Not included | Outcome not measured clearly |
|  | Observational and Genetic Associations of Body Mass Index and Hepatobiliary Diseases in a Relatively Lean Chinese Population | Not included | Unrelated outcome |
|  | Prevalence, risk factors and virological profile of chronic hepatitis B virus infection in pregnant women in India | Not included | Study done in other setting |
|  | A novel system for predicting liver histopathology in patients with chronic hepatitis B | Not included | Outcome not measured clearly |
|  | Hepatitis B immunization data of patients living with HIV/AIDS: a multi-centre study | Not included | Unrelated outcome |
|  | Human immunodeficiency virus infection predictors and genetic diversity of hepatitis B virus and hepatitis C virus co-infections among drug users in three major Kenyan cities | Not included | Unrelated outcome |
|  | Global epidemiology of hepatitis B virus infection: new estimates of age-specific HBsAg seroprevalence and endemicity | Not included | Outcome not measured clearly |
|  | Exposure time to hepatitis B virus and associated risk factors among children in Edirne, Turkey | Not included | Study done in other setting |
|  | Hepatitis B virus infection among illegal drug users in Enugu State, Nigeria: prevalence, immune status, and related risk factors | Not included | Study done in other population |
|  | Staff Nurse Education on Best Practices for Preventing Blood-Borne Pathogen Exposures | Not included | Unrelated outcome |
|  | Evaluating vertical transmission of sexually transmitted infections to newborns | Not included | Unrelated outcome |
|  | A systematic review and meta-analysis of the prevalence of hepatitis B virus infection among pregnant women in Nigeria | Not included | It is not a primary study |
|  | Knowledge, attitude, and risk factors of hepatitis B among waste scavengers in Lagos, Nigeria | Not included | Unrelated outcome |
|  | A multi-centre cross-sectional study on hepatitis B vaccination coverage and associated factors among personnel working in health facilities in Kumasi, Ghana | Not included | Unrelated outcome |
|  | The impact of HIV on hepatocellular cancer survival in Nigeria | Not included | Unrelated outcome |
|  | Factors Influencing Hospital Cleaners’ Knowledge and Practices toward Hepatitis B prevention in Northern Province of Rwanda | Not included | Unrelated outcome |
|  | A proposed predictive model for advanced fibrosis in patients with chronic hepatitis B and its validation | Not included | Unrelated outcome |
|  | Hepatitis C prevalence and associated risk factors among individuals who are homeless and diagnosed with mental illness: At Home/Chez Soi Study, Vancouver, BC | Not included | Unrelated outcome |
|  | Hepatitis A vaccines | Not included | It is not study |
|  | Prevalence of hepatitis B virus infection in Nigeria, 2000-2013: A systematic review and meta-analysis | Not included | The study is not primary study |
|  | Seroprevalence of hepatitis B virus among antenatal clinic attendees in Gamawa Local Government Area, Bauchi State, Nigeria | Not included | Study done in other population |
|  | Syphilis and HIV prevalence and associated factors to their co-infection, hepatitis B and hepatitis C viruses prevalence among female sex workers in Rwanda | Not included | Outcome not measured clearly |
|  | Prevalence of Hepatitis B Virus (HBV) surface antigen and HBVassociated hepatocellular carcinoma in Kenyans of various ages | Not included | Study done in general population |
|  | A nationwide cross-sectional review of in-hospital hepatitis B virus testing and disease burden estimation in Ghana, 2016 - 2021 | Not included | It is not primary study |
|  | Studies on prevalence and risk factors for Hepatitis B Surface Antigen among secondary school students in north-central, Nigeria | Not included | Stud done in other population |
|  | Multidimensional Analysis of the Mother-to-child Transmission Risk Factors in Chronic Hepatitis B Virus Infection in Pregnant Women in Vietnam | Not included | Study done in other setting |
|  | Healthcare resource utilization and costs by disease severity in an insured national sample of US patients with chronic hepatitis B | Not included | Unrelated outcome |
|  | Advancing Age and Comorbidity in a US Insured Population-Based Cohort of Patients With Chronic Hepatitis B | Not included | Unrelated outcome |
|  | Hepatitis B‐related hepatocellular carcinoma: epidemiological characteristics and disease burden | Not included | Stud done in other population |
|  | An enormous hepatitis B virus‐related liver disease burden projected in Vietnam by 2025 | Not included | Study done in other setting |
|  | Hepatitis B in healthcare personnel: an update on the global landscape | Not included | Outcome not measured clearly |
|  | Prevalence, infectivity and correlates of hepatitis B virus infection among pregnant women in a rural district of the Far North Region of Cameroon | Not included | Study done in other population |
|  | Assessing risk behaviors and prevalence of sexually transmitted and blood-borne infections among female crack cocaine users in Salvador-Bahia, Brazil | Not included | Study done in other setting |
|  | Standard Precautions among HealthCare Workers in a Tertiary Health Facility in Enugu Metropolis, South-East Nigeria | Not included | Unrelated outcome |
|  | HIV viraemia during hepatitis B vaccination shortens the duration of protective antibody levels | Not included | Unrelated outcome |
|  | Viral load is a significant prognostic factor for hepatitis B virus‐associated hepatocellular carcinoma | Not included | Unrelated outcome |
|  | Dual positivity of hepatitis B surface antigen and anti-hepatitis C virus antibody and associated factors among apparently healthy patients of Ekiti State, Nigeria | Not included | Unrelated outcome |
|  | Hepatitis C virus infection and its associated factors among prisoners in a Nigerian prison | Not included | Unrelated outcome |
|  | Early cranial ultrasound findings among infants with neonatal encephalopathy in Uganda: an observational study | Not included | Unrelated outcome |
|  | Sero-prevalence of hepatitis B virus and associated factors among pregnant women in Gambella hospital, South Western Ethiopia: facility based cross-sectional study | Not included | Study done in other population |
|  | Clinical characteristics and current management of hepatitis B and C in China | Not included | Study done in other setting |
|  | No contribution of lifestyle and environmental exposures to gender discrepancy of liver disease severity in chronic hepatitis b infection: Observations from the Haimen City cohort | Not included | Unrelated outcome |
|  | Sero-Prevalence of Hepatitis B Virus Infection and Associated Factors Among Pregnant Women Attending Antenatal Care Services in Gedeo Zone, Southern Ethiopia | Not included | Study done in other population |
|  | Effects of long-term antiretroviral therapy in reproductive-age women in sub-Saharan Africa (the PEPFAR PROMOTE study): a multi-country observational cohort study | Not included | Unrelated study |
|  | Prevalence, genotype distribution and mutations of hepatitis B virus and the associated risk factors among pregnant women residing in the northern shores of Persian Gulf, Iran | Not included | Study done in other setting |
|  | Global Estimates on Biological Risks at Work | Not included | It is not primary study |
|  | Telbivudine treatment of hepatitis B virus-infected pregnant women at different gestational stages for the prevention of mother-to-child transmission: Outcomes of telbivudine treatment during pregnancy | Not included | Unrelated outcome |
|  | Occult Hepatitis B Virus Infection in Maintenance Hemodialysis Patients: Prevalence and Mutations in "a" Determinant | Not included | Study done in other population |
|  | Prevalence of hepatitis B virus infection in Shenzhen, China, 2015–2018 | Not included | Study done in other setting |
|  | Identifying, preventing and controlling needle-stick injuries in Indonesia | Not included | Unrelated outcome |
|  | Splash of body fluids among healthcare support staff in Ghana: a cross-sectional study | Not included | Unrelated outcome |
|  | Physiological and psychosocial stressors among hemodialysis patients in the Buea Regional Hospital, Cameroon | Not included | Unrelated outcome |
|  | Safety practice and associated factors among waste handlers in Governmental Hospitals in Addis Ababa, Ethiopia | Not included | Unrelated outcome |
|  | Hepatitis B virus infection and associated risk factors among medical students in eastern Ethiopia | Not included | Study done in other population |
|  | Risk factors associated with Hepatitis B virus infection among pregnant women attending public hospitals in Addis Ababa, Ethiopia | Not included | Study done in other population |
|  | Clinicopathological analysis of patients with dual malignancies: A retrospective study | Not included | Study done in other population |
|  | Prevalence and the associated factors of hepatitis B and hepatitis C viral infections among HIV-positive individuals in same-day antiretroviral therapy initiation program in Bangkok, Thailand | Not included | Study done in other setting |
|  | Prevalence of liver steatosis in patients with chronic hepatitis B: a study of associated factors and of relationship with fibrosis | Not included | Unrelated outcome |
|  | Hepatitis B, hepatitis C, and mortality among HIV-positive individuals | Not included | Outcome not measured clearly |
|  | Nephrotoxicity caused by oral antiviral agents in patients with chronic hepatitis B treated in a hospital for tropical diseases in Thailand | Not included | Study done in other setting |
|  | Sexual behaviour and practices among adolescent blood donors in Harare and Masvingo provinces, Zimbabwe | Not included | Outcome not measured clearly |
|  | Thermal disinfection in hemodialysis using the A0 concept as dispenser | Not included | Unrelated study |
|  | Foodborne Diseases: Overview of Biological Hazards and Foodborne Diseases | Not included | It is not primary study |
|  | Guidelines for Preventing Infectious Complications among Hematopoietic Cell Transplantation Recipients: A Global Perspective | Not included | It is not study |
|  | Barriers to hepatitis B vaccine coverage among healthcare workers in the Republic of Georgia: An international perspective | Not included | Unrelated study |
|  | The impact of economic and social factors on the prevalence of hepatitis B in Turkey | Not included | Study done in other setting |
|  | Proliferative lupus nephritis in the absence of overt systemic lupus erythematosus: A historical study of 12 adult patients | Not included | Unrelated study |
|  | Seroprevalence of hepatitis B and C virus infections and risk factors in Turkey: a fieldwork TURHEP study | Not included | Study done in other setting |
|  | Chronic liver disease detection and quantification | Not included | Outcome not measured clearly |
|  | Inequalities in the use of secondary prevention of cardiovascular disease by socioeconomic status: evidence from the PURE observational study | Not included | Outcome not measured clearly |
|  | Novel point-of-care cytokine biomarker lateral flow test for the screening for sexually transmitted infections and bacterial vaginosis: study protocol of a multicentre multidisciplinary prospective observational clinical study to evaluate the performance and feasibility of the Genital InFlammation Test (GIFT) | Not included | Duplicate |
|  | Existing gaps and missed opportunities in delivering quality nutrition services in primary healthcare: a descriptive analysis of patient experience and provider competence in 11 low-income and middle-income countries | Not included | Duplicate |
|  | Capacity and quality of maternal and child health services delivery at the subnational primary healthcare level in relation to intermediate health outputs: a cross-sectional study of 12 low-income and middle-income countries | Not included | Duplicate |
|  | Waste Management With Special emphasis on occupational health and safety at selected healthcare establishments | Not included | Duplicate |
|  | Factors affecting the serological testing of cadaveric donor cornea | Not included | Duplicate |
|  | Identifying occupational hazards among healthcare workers in Australia and Bhutan | Not included | Duplicate |
|  | Chapter 30 - Occupational Health of Laboratory Animal Workers | Not included | Duplicate |
|  | Prevalence of hepatitis B and C viral infections in Pakistan: findings of a national survey appealing for effective prevention and control measures | Not included | Duplicate |
|  | Immunizations and oral health care providers | Not included | Duplicate |
|  | A positive-feedback loop between HBx and ALKBH5 promotes hepatocellular carcinogenesis | Not included | Duplicate |
|  | Serum Liver Fibrosis Markers in the Prognosis of Liver Cirrhosis: A Prospective Observational Study | Not included | Duplicate |
|  | Expansion of Stem Cell-Like CD4(+) Memory T Cells during Acute HIV-1 Infection Is Linked to Rapid Disease Progression | Not included | Duplicate |
|  | Cancer risk by social class and occupation: a survey of 109,000 cancer cases among Finns of working age | Not included | Duplicate |
|  | Improved Antibody Response to Three Additional Hepatitis B Vaccine Doses Following Primary Vaccination Failure in Patients with Inflammatory Bowel Disease | Not included | Duplicate |
|  | Sleep and antibody response to hepatitis B vaccination | Not included | Duplicate |
|  | Emerging and Re-emerging Pathogens and Diseases, and Health Consequences of a Changing Climate | Not included | Duplicate |
|  | Nurses and AIDS care: Occupational risk perception and the social construction of HIV | Not included | Duplicate |
|  | Liver steatosis in children with chronic hepatitis B and C: Prevalence, predictors, and impact on disease progression | Not included | Duplicate |
|  | Prevalence of hepatitis B virus (HBV) infection among Makerere University medical students | Not included | Duplicate |
|  | Suppl-1, M3: epidemiology of hepatitis B virus (HBV) and hepatitis C virus (HCV) related hepatocellular carcinoma | Not included | Duplicate |
|  | Hepatitis B virus infection and vaccine-induced immunity in Madrid (Spain) | Not included | Duplicate |
|  | Costs of needlestick injuries and subsequent hepatitis and HIV infection | Not included | Duplicate |
|  | To Study the Incidence, Predictive Factors and Clinical Outcome of Spontaneous Bacterial Peritonitis in Patients of Cirrhosis with Ascites | Not included | Duplicate |
|  | Delayed-type hypersensitivity and hepatitis B vaccine responses, in vivo markers of cellular and humoral immune function, and the risk of AIDS or death | Not included | Duplicate |
|  | Sharps injuries among hospital workers in Massachusetts, 2007 | Not included | Duplicate |
|  | Clinical features of HBsAg seroclearance in hepatitis B virus carriers in South Korea: A retrospective longitudinal study | Not included | Duplicate |
|  | Global patterns of hepatocellular carcinoma management from diagnosis to death: the BRIDGE Study | Not included | Duplicate |
|  | Assessing significant fibrosis using imaging-based elastography in chronic hepatitis B patients: Pilot study | Not included | Duplicate |
|  | Liver Transplantation from Brain-Dead Donors with Hepatitis B or C in South Korea: A 2014-2020 Korean Organ Transplantation Registry Data Analysis | Not included | Duplicate |
|  | Ten-Year Changes in the Hepatitis B Prevalence in the Birth Cohorts in Korea: Results From Nationally Representative Cross-Sectional Surveys | Not included | Duplicate |
|  | A Study to Assess the Knowledge and Attitude Regarding Occupational Exposure and Post Exposure Prophylaxis (PEP) for Hiv Among Student Nurses of Selected Nursing Institutes of Hubballi with a View to Develop an Information Guide Sheet | Not included | Duplicate |
|  | Observational and Genetic Associations of Body Mass Index and Hepatobiliary Diseases in a Relatively Lean Chinese Population | Not included | Duplicate |
|  | Prevalence, risk factors and virological profile of chronic hepatitis B virus infection in pregnant women in India | Not included | Duplicate |
|  | A novel system for predicting liver histopathology in patients with chronic hepatitis B | Not included | Duplicate |
|  | Hepatitis B immunization data of patients living with HIV/AIDS: a multi-centre study | Not included | Duplicate |
|  | Human immunodeficiency virus infection predictors and genetic diversity of hepatitis B virus and hepatitis C virus co-infections among drug users in three major Kenyan cities | Not included | Duplicate |
|  | Global epidemiology of hepatitis B virus infection: new estimates of age-specific HBsAg seroprevalence and endemicity | Not included | Duplicate |
|  | Exposure time to hepatitis B virus and associated risk factors among children in Edirne, Turkey | Not included | Duplicate |
|  | Hepatitis B virus infection among illegal drug users in Enugu State, Nigeria: prevalence, immune status, and related risk factors | Not included | Duplicate |
|  | Staff Nurse Education on Best Practices for Preventing Blood-Borne Pathogen Exposures | Not included | Duplicate |
|  | Evaluating vertical transmission of sexually transmitted infections to newborns | Not included | Duplicate |
| 1. 7 | A systematic review and meta-analysis of the prevalence of hepatitis B virus infection among pregnant women in Nigeria | Not included | Duplicate |
|  | Knowledge, attitude, and risk factors of hepatitis B among waste scavengers in Lagos, Nigeria | Not included | Duplicate |
|  | A multi-centre cross-sectional study on hepatitis B vaccination coverage and associated factors among personnel working in health facilities in Kumasi, Ghana | Not included | Duplicate |
|  | The impact of HIV on hepatocellular cancer survival in Nigeria | Not included | Duplicate |
|  | Factors Influencing Hospital Cleaners’ Knowledge and Practices toward Hepatitis B prevention in Northern Province of Rwanda | Not included | Duplicate |
|  | A proposed predictive model for advanced fibrosis in patients with chronic hepatitis B and its validation | Not included | Duplicate |
|  | Hepatitis C prevalence and associated risk factors among individuals who are homeless and diagnosed with mental illness: At Home/Chez Soi Study, Vancouver, BC | Not included | Duplicate |
|  | Hepatitis A vaccines | Not included | Duplicate |
|  | Prevalence of hepatitis B virus infection in Nigeria, 2000-2013: A systematic review and meta-analysis | Not included | Duplicate |
|  | Seroprevalence of hepatitis B virus among antenatal clinic attendees in Gamawa Local Government Area, Bauchi State, Nigeria | Not included | Duplicate |
|  | Syphilis and HIV prevalence and associated factors to their co-infection, hepatitis B and hepatitis C viruses prevalence among female sex workers in Rwanda | Not included | Duplicate |
|  | Prevalence of Hepatitis B Virus (HBV) surface antigen and HBVassociated hepatocellular carcinoma in Kenyans of various ages | Not included | Duplicate |
|  | A nationwide cross-sectional review of in-hospital hepatitis B virus testing and disease burden estimation in Ghana, 2016 - 2021 | Not included | Duplicate |
|  | Studies on prevalence and risk factors for Hepatitis B Surface Antigen among secondary school students in north-central, Nigeria | Not included | Duplicate |
|  | Multidimensional Analysis of the Mother-to-child Transmission Risk Factors in Chronic Hepatitis B Virus Infection in Pregnant Women in Vietnam | Not included | Duplicate |
|  | Healthcare resource utilization and costs by disease severity in an insured national sample of US patients with chronic hepatitis B | Not included | Duplicate |
|  | Advancing Age and Comorbidity in a US Insured Population-Based Cohort of Patients With Chronic Hepatitis B | Not included | Duplicate |
|  | Hepatitis B‐related hepatocellular carcinoma: epidemiological characteristics and disease burden | Not included | Duplicate |
|  | An enormous hepatitis B virus‐related liver disease burden projected in Vietnam by 2025 | Not included | Duplicate |
|  | Hepatitis B in healthcare personnel: an update on the global landscape | Not included | Duplicate |
|  | Prevalence, infectivity and correlates of hepatitis B virus infection among pregnant women in a rural district of the Far North Region of Cameroon | Not included | Duplicate |
|  | Assessing risk behaviors and prevalence of sexually transmitted and blood-borne infections among female crack cocaine users in Salvador-Bahia, Brazil | Not included | Duplicate |
|  | Standard Precautions among HealthCare Workers in a Tertiary Health Facility in Enugu Metropolis, South-East Nigeria | Not included | Duplicate |
|  | HIV viraemia during hepatitis B vaccination shortens the duration of protective antibody levels | Not included | Duplicate |
|  | Viral load is a significant prognostic factor for hepatitis B virus‐associated hepatocellular carcinoma | Not included | Duplicate |
|  | Dual positivity of hepatitis B surface antigen and anti-hepatitis C virus antibody and associated factors among apparently healthy patients of Ekiti State, Nigeria | Not included | Duplicate |
|  | Hepatitis C virus infection and its associated factors among prisoners in a Nigerian prison | Not included | Duplicate |
|  | Early cranial ultrasound findings among infants with neonatal encephalopathy in Uganda: an observational study | Not included | Duplicate |
|  | Sero-prevalence of hepatitis B virus and associated factors among pregnant women in Gambella hospital, South Western Ethiopia: facility based cross-sectional study | Not included | Duplicate |
|  | Clinical characteristics and current management of hepatitis B and C in China | Not included | Duplicate |
|  | No contribution of lifestyle and environmental exposures to gender discrepancy of liver disease severity in chronic hepatitis b infection: Observations from the Haimen City cohort | Not included | Duplicate |
|  | Sero-Prevalence of Hepatitis B Virus Infection and Associated Factors Among Pregnant Women Attending Antenatal Care Services in Gedeo Zone, Southern Ethiopia | Not included | Duplicate |
|  | Effects of long-term antiretroviral therapy in reproductive-age women in sub-Saharan Africa (the PEPFAR PROMOTE study): a multi-country observational cohort study | Not included | Duplicate |
|  | Prevalence, genotype distribution and mutations of hepatitis B virus and the associated risk factors among pregnant women residing in the northern shores of Persian Gulf, Iran | Not included | Duplicate |
|  | Global Estimates on Biological Risks at Work | Not included | Duplicate |
|  | Telbivudine treatment of hepatitis B virus-infected pregnant women at different gestational stages for the prevention of mother-to-child transmission: Outcomes of telbivudine treatment during pregnancy | Not included | Duplicate |
|  | Occult Hepatitis B Virus Infection in Maintenance Hemodialysis Patients: Prevalence and Mutations in "a" Determinant | Not included | Duplicate |
|  | Prevalence of hepatitis B virus infection in Shenzhen, China, 2015–2018 | Not included | Duplicate |
|  | Identifying, preventing and controlling needle-stick injuries in Indonesia | Not included | Duplicate |
|  | Splash of body fluids among healthcare support staff in Ghana: a cross-sectional study | Not included | Duplicate |
|  | Physiological and psychosocial stressors among hemodialysis patients in the Buea Regional Hospital, Cameroon | Not included | Duplicate |
|  | Safety practice and associated factors among waste handlers in Governmental Hospitals in Addis Ababa, Ethiopia | Not included | Duplicate |
|  | Hepatitis B virus infection and associated risk factors among medical students in eastern Ethiopia | Not included | Duplicate |
|  | Risk factors associated with Hepatitis B virus infection among pregnant women attending public hospitals in Addis Ababa, Ethiopia | Not included | Duplicate |
|  | Clinicopathological analysis of patients with dual malignancies: A retrospective study | Not included | Duplicate |
|  | Prevalence and the associated factors of hepatitis B and hepatitis C viral infections among HIV-positive individuals in same-day antiretroviral therapy initiation program in Bangkok, Thailand | Not included | Duplicate |
|  | Prevalence of liver steatosis in patients with chronic hepatitis B: a study of associated factors and of relationship with fibrosis | Not included | Duplicate |
|  | Hepatitis B, hepatitis C, and mortality among HIV-positive individuals | Not included | Duplicate |
|  | Nephrotoxicity caused by oral antiviral agents in patients with chronic hepatitis B treated in a hospital for tropical diseases in Thailand | Not included | Duplicate |
|  | Sexual behaviour and practices among adolescent blood donors in Harare and Masvingo provinces, Zimbabwe | Not included | Duplicate |
|  | Thermal disinfection in hemodialysis using the A0 concept as dispenser | Not included | Duplicate |
|  | Foodborne Diseases: Overview of Biological Hazards and Foodborne Diseases | Not included | Duplicate |
|  | Guidelines for Preventing Infectious Complications among Hematopoietic Cell Transplantation Recipients: A Global Perspective | Not included | Duplicate |
|  | Barriers to hepatitis B vaccine coverage among healthcare workers in the Republic of Georgia: An international perspective | Not included | Duplicate |
|  | The impact of economic and social factors on the prevalence of hepatitis B in Turkey | Not included | Duplicate |
|  | Proliferative lupus nephritis in the absence of overt systemic lupus erythematosus: A historical study of 12 adult patients | Not included | Duplicate |
|  | Seroprevalence of hepatitis B and C virus infections and risk factors in Turkey: a fieldwork TURHEP study | Not included | Duplicate |
|  | Chronic liver disease detection and quantification | Not included | Duplicate |
|  | Inequalities in the use of secondary prevention of cardiovascular disease by socioeconomic status: evidence from the PURE observational study | Not included | Duplicate |
|  | Distinct forms of migration and mobility are differentially associated with HIV treatment adherence | Not included | Duplicate |
|  | Iron Status and Associated Malaria Risk Among African Children | Not included | Duplicate |
|  | Comparison of viral hepatitis-associated hepatocellular carcinoma due to HBV and HCV - cohort from liver clinics in Pakistan | Not included | Duplicate |
|  | Factors associated with anti-hepatitis A virus immunoglobulin G seropositivity among Korean workers: a cross-sectional study | Not included | Duplicate |
|  | Uptake of long acting reversible contraception following integrated couples HIV and fertility goal-based family planning counselling in Catholic and non-Catholic, urban and rural government health centers in Kigali, Rwanda | Not included | Duplicate |
|  | Infection control practices in clinical laboratories in Pakistan | Not included | Duplicate |
|  | Uptake of long acting reversible contraception following integrated couples HIV and fertility goal-based family planning counselling in Catholic and non-Catholic, urban and rural government health centers in Kigali, Rwanda | Not included | Duplicate |
|  | Prevalence and Factors Associated with Percutaneous Injuries and Splash Exposures among Health-Care Workers in Rift Valley Provincial and War Memorial Hospitals, Kenya | Not included | Duplicate |
|  | Prevalence of hepatitis B virus infection in the Gezira state of central Sudan | Not included | Duplicate |
|  | Abdominal surgical site infections: a prospective study of determinant factors in Harare, Zimbabwe | Not included | Duplicate |
|  | Innate Immune Responses in Viral Hepatitis: the role of Kupffer cells and liver-derived monocytes in shaping intrahepatic immunity in mice using the LCMV infection model | Not included | Duplicate |
|  | Post exposure prophylaxis following occupational exposure to HIV: a survey of health care workers in Mbeya, Tanzania, 2009-2010 | Not included | Duplicate |
|  | Value of early change of serum C reactive protein combined to modified Alvarado score in the diagnosis of acute appendicitis | Not included | Duplicate |
|  | Needle Stick and Sharps Injuries among Healthcare Workers in an Oncology Setting: A Retrospective Seven-Year Study | Not included | Duplicate |
|  | Clinical presentation of pregnant women in isolation units for Ebola virus disease in Sierra Leone, 2014 | Not included | Duplicate |
|  | Paenibacillus spp infection among infants with postinfectious hydrocephalus in Uganda: an observational case-control study | Not included | Duplicate |
|  | Medication exposure during pregnancy: a pilot pharmacovigilance system using health and demographic surveillance platform | Not included | Duplicate |
|  | Safety of artemether-lumefantrine exposure in first trimester of pregnancy: an observational cohort | Not included | Duplicate |
|  | The independent effect of living in malaria hotspots on future malaria infection: an observational study from Misungwi, Tanzania | Not included | Duplicate |
|  | Trends of frequency, mortality and risk factors among patients admitted with stroke from 2017 to 2019 to the medical ward at Kilimanjaro Christian Medical Centre hospital: a retrospective observational study | Not included | Duplicate |
|  | Case-control study of risk factors for avian influenza A (H5N1) disease, Hong Kong, 1997 | Not included | Duplicate |
|  | Prevalence of syphilis, human immunodeficiency virus, hepatitis B virus, and human T-lymphotropic virus infections and coinfections during prenatal screening in an urban Northeastern Brazilian population | Not included | Duplicate |
|  | Plasmodium falciparum resistance and malaria presentation in children at Dongola specialist hospital: A prospective cohort study | Not included | Duplicate |
|  | Innate Immune Responses in Viral Hepatitis: the role of Kupffer cells and liver-derived monocytes in shaping intrahepatic immunity in mice using the LCMV infection model | Not included | Duplicate |
|  | Maternal hookworm modifies risk factors for childhood eczema: results from a birth cohort in Uganda | Not included | Duplicate |
|  | Clinical presentation of pregnant women in isolation units for Ebola virus disease in Sierra Leone, 2014 | Not included | Duplicate |
|  | Post exposure prophylaxis following occupational exposure to HIV: a survey of health care workers in Mbeya, Tanzania, 2009-2010 | Not included | Duplicate |
|  | Development of a Web Application based on Machine Learning for screening esophageal varices in cirrhosis | Not included | Duplicate |
|  | Value of early change of serum C reactive protein combined to modified Alvarado score in the diagnosis of acute appendicitis | Not included | Duplicate |
|  | Needlestick and sharps injuries among healthcare workers in an oncology setting: a retrospective 7-year cross-sectional study | Not included | Duplicate |
|  | Needlestick and sharps injuries among healthcare workers in an oncology setting: a retrospective 7-year cross-sectional study | Not included | Duplicate |
|  | Abdominal surgical site infections: a prospective study of determinant factors in Harare, Zimbabwe | Not included | Duplicate |
|  | Prevalence of hepatitis B virus infection in the Gezira state of central Sudan | Not included | Duplicate |
|  | Impact of the Safe Childbirth Checklist on health worker childbirth practices in Luapula province of Zambia: a pre-post study | Not included | Duplicate |
|  | Prevalence and Factors Associated with Percutaneous Injuries and Splash Exposures among Health-Care Workers in Rift Valley Provincial and War Memorial Hospitals, Kenya | Not included | Duplicate |
|  | Knowledge, attitudes, practices and prevalence of hepatitis B and C and hepatitis B vaccination coverage among public sector healthcare workers in Cambodia | Not included | Duplicate |
|  | Natural History of Untreated HBeAg-Positive Chronic HBV Infection With Persistently Elevated HBV DNA but Normal Alanine Aminotransferase | Not included | Duplicate |
|  | Pretransplant Hepatitis B Viral Infection Increases Risk of Death After Kidney Transplantation: A Multicenter Cohort Study in Korea | Not included | Duplicate |
|  | Adefovir- or Lamivudine-Induced Renal Tubular Dysfunction after Liver Transplantation | Not included | Duplicate |
|  | Prevalence of hepatitis B virus and associated risk factors among adults patients at Dessie referral and Kemise general hospitals in northeastern Ethiopia | Not included | Duplicate |
|  | Childhood vaccination coverage and regional differences in Swiss birth cohorts 2012-2021: Are we on track? | Not included | Duplicate |
|  | The renal angina index accurately predicts low risk of developing severe acute kidney injury among children admitted to a low-resource pediatric intensive care unit | Not included | Duplicate |
|  | Characteristics, complications, and gaps in evidence-based interventions in rheumatic heart disease: the Global Rheumatic Heart Disease Registry (the REMEDY study) | Not included | Duplicate |
|  | Occupational stress and health among home health care workers | Not included | Duplicate |
|  | The Safety and Immunogenicity of the mRNA-BNT162b2 SARS-CoV-2 Vaccine in Hemodialysis Patients | Not included | Duplicate |
|  | Association of Combined Tobacco Smoking, Hormonal Contraceptive use and Status Matrimonial with Cervical Cancer Evolution in Tunisian Women | Not included | Duplicate |
|  | Treatment effects of the differential first-line antiretroviral regimens among HIV/HBV coinfected patients in southwest China: an observational study | Not included | Duplicate |
|  | Long-term survival and recurrence after curative resection for hepatocellular carcinoma in patients with chronic hepatitis C virus infection: a multicenter observational study from China | Not included | Duplicate |
|  | Risk factors for combined hepatocellular-cholangiocarcinoma: a hospital-based case-control study | Not included | Duplicate |
|  | Humoral immune responses to inactivated COVID-19 vaccine up to 1 year in children with chronic hepatitis B infection | Not included | Duplicate |
|  | Validation of the Korean Stroop Test in Diagnosis of Minimal Hepatic Encephalopathy | Not included | Duplicate |
|  | Seroprevalence and predictors of hepatitis B virus infection among pregnant women attending routine antenatal care in Arba Minch Hospital, South Ethiopia | Not included | Duplicate |
|  | Conservation and variability of hepatitis B core at different chronic hepatitis stages | Not included | Duplicate |
|  | Prevalence of Hepatitis B virus infection and its determinants among pregnant women in East Africa: Systematic review and Meta-analysis | Not included | Duplicate |
|  | HCC risk reduction with oral nucleos(t)ide analogues in patients with chronic hepatitis B: Not perfect, not good enough | Not included | Duplicate |
|  | Occupational exposures to blood and body fluids (BBFS) among health care workers and medical students in University of Gondar Hospital, Northwest of Ethiopia | Not included | Duplicate |
|  | Somatosensory Amplification, Anxiety, and Depression in Patients With Hepatitis B: Impact on Functionality | Not included | Duplicate |
|  | Health behaviors of Korean adults with hepatitis B: Findings of the 2016 Korean National Health and Nutrition Examination Survey | Not included | Duplicate |
|  | Prevalence of and risk factors for hepatitis C virus antibody among people who inject drugs in Cambodia: a national biological and behavioral survey | Not included | Duplicate |
|  | An updated systematic review and meta-analysis of the prevalence of hepatitis B virus in Ethiopia | Not included | Duplicate |
|  | Management of Takotsubo cardiomyopathy in non-academic hospitals in France: The Observational French SyndromEs of TakoTsubo (OFSETT) study | Not included | Duplicate |
|  | Clinical characteristics, prognosis, and surgical outcomes of patients with non-HBV and non-HCV related hepatocellular carcinoma: three-decade observational study | Not included | Duplicate |
|  | Characteristics of female sexual dysfunctions and obstetric complications related to female genital mutilation in Omdurman maternity hospital, Sudan | Not included | Duplicate |
|  | Prognostic value of immunoscore to identify mortality outcomes in adults with HBV-related primary hepatocellular carcinoma | Not included | Duplicate |
|  | The association of adverse outcomes in the mother with disease progression in offspring in families with clusters of hepatitis B virus infection and unfavorable prognoses in Northwest China | Not included | Duplicate |
|  | Hospital acquired infections and infection prevention practices in teaching hospitals in the Amhara regional state, Ethiopia | Not included | Duplicate |
|  | Sero-prevalence of hepatitis B virus infection and its risk factors among pregnant women attending antenatal clinic at Aminu Kano Teaching Hospital, Kano, Nigeria | Not included | Duplicate |
|  | Relationship of Treg/Th17 balance with HBeAg change in HBeAg-positive chronic hepatitis B patients receiving telbivudine antiviral treatment: A longitudinal observational study | Not included | Duplicate |
|  | Occupational exposures to blood and body fluids (BBFS) among health care workers and medical students in University of Gondar Hospital, Northwest of Ethiopia | Not included | Duplicate |
|  | Telbivudine and adefovir dipivoxil combination therapy improves renal function in patients with chronic hepatitis B: A STROBE-compliant article | Not included | Duplicate |
|  | Outcomes of Glucocorticoid Treatment in HBV Associated Acute-on-Chronic Liver Failure Patients: A Retrospective Observational Study | Not included | Duplicate |
|  | Multimodal Ultrasound Model Based on the Left Gastric Vein in B-Viral Cirrhosis: Noninvasive Prediction of Esophageal Varices | Not included | Duplicate |
|  | Multiple Primary Malignancies in Patients With Hepatocellular Carcinoma: A Largest Series With 26-Year Follow-Up | Not included | Duplicate |
|  | Expression profiles of transcription factors for special CD4+ T-cell subsets in peripheral blood mononuclear cells from patients with hepatitis B virus infection | Not included | Duplicate |
|  | Hepatitis B virus-associated hepatocellular carcinoma | Not included | Duplicate |
|  | HBV pgRNA profiles in Chinese HIV/HBV coinfected patients under pre- and posttreatment: a multicentre observational cohort study | Not included | Duplicate |
|  | Population-Based Multicentric Survey of Hepatitis B Infection and Risk Factors in the North, South, and Southeast Regions of Brazil, 10-20 Years After the Beginning of Vaccination | Not included | Duplicate |
|  | Analysis of serum hepatitis B virus RNA levels among HBsAg and HBsAb copositive patients and its correlation with HBV DNA | Not included | Duplicate |
|  | Global Burden and Trends of Primary Liver Cancer Attributable to Comorbid Type 2 Diabetes Mellitus Among People Living with Hepatitis B: An Observational Trend Study from 1990 to 2019 | Not included | Duplicate |
|  | Systemic immune-inflammation index predicts postoperative acute kidney injury in hepatocellular carcinoma patients after hepatectomy | Not included | Duplicate |
|  | The imported infections among foreign travelers in China: an observational study | Not included | Duplicate |
|  | An updated systematic review and meta-analysis of the prevalence of hepatitis B virus in Ethiopia | Not included | Duplicate |
|  | Hospital acquired infections and infection prevention practices in teaching hospitals in the Amhara regional state, Ethiopia | Not included | Duplicate |
|  | 2022 International Consensus on Cardiopulmonary Resuscitation and Emergency Cardiovascular Care Science With Treatment Recommendations: Summary From the Basic Life Support; Advanced Life Support; Pediatric Life Support; Neonatal Life Support; Education, Implementation, and Teams; and First Aid Task Forces | Not included | Duplicate |
|  | Mercury Exposure and Poor Nutritional Status Reduce Response to Six Expanded Program on Immunization Vaccines in Children: An Observational Cohort Study of Communities Affected by Gold Mining in the Peruvian Amazon | Not included | Duplicate |
|  | Maternal exposure to carbon monoxide and fine particulate matter during pregnancy in an urban Tanzanian cohort | Not included | Duplicate |
|  | Sero-prevalence of hepatitis B virus infection and its risk factors among pregnant women attending antenatal clinic at Aminu Kano Teaching Hospital, Kano, Nigeria | Not included | Duplicate |
|  | Hepatitis B virus-associated hepatocellular carcinoma | Not included | Duplicate |
|  | Add-on pegylated interferon augments hepatitis B surface antigen clearance vs continuous nucleos(t)ide analog monotherapy in Chinese patients with chronic hepatitis B and hepatitis B surface antigen ≤ 1500 IU/mL: An observational study | Not included | Duplicate |
|  | Pneumococcal and influenza vaccination coverage among at-risk adults: A 5-year French national observational study | Not included | Duplicate |
|  | A modified MELD model for Chinese pre-ACLF and ACLF patients and it reveals poor prognosis in pre-ACLF patients | Not included | Duplicate |
|  | Analysis of serum hepatitis B virus RNA levels among HBsAg and HBsAb copositive patients and its correlation with HBV DNA | Not included | Duplicate |
|  | Statins improve outcomes of nonsurgical curative treatments in hepatocellular carcinoma patients | Not included | Duplicate |
|  | Genome-wide study of salivary microRNAs as potential noninvasive biomarkers for detection of nasopharyngeal carcinoma | Not included | Duplicate |
|  | Comparison of the efficacy and safety of entecavir and tenofovir in nucleos(t)ide analogue-naive chronic hepatitis B patients with high viraemia: a retrospective cohort study | Not included | Duplicate |
|  | Multicenter study of skin rashes and hepatotoxicity in antiretroviral-naïve HIV-positive patients receiving non-nucleoside reverse-transcriptase inhibitor plus nucleoside reverse-transcriptase inhibitors in Taiwan | Not included | Duplicate |
|  | Methylation status of the stimulator of interferon genes promoter in patients with chronic hepatitis B | Not included | Duplicate |
|  | Postnatal infection surveillance by telephone in Dar es Salaam, Tanzania: An observational cohort study | Not included | Duplicate |
|  | MRI findings in people with epilepsy and nodding syndrome in an area endemic for onchocerciasis: an observational study | Not included | Duplicate |
|  | Bacterial pathogenesis: a molecular approach | Not included | Duplicate |
|  | Clinical features of treatment-naive patients with hepatitis B virus infection: A community-based survey from high- and intermediate-hepatitis B endemicity regions in Southeast China | Not included | Duplicate |
|  | Rituximab plus chemotherapy as first-line treatment in Chinese patients with diffuse large B-cell lymphoma in routine practice: a prospective, multicentre, non-interventional study | Not included | Duplicate |
|  | Albumin-bilirubin and platelet-albumin-bilirubin grades for hepatitis B-associated hepatocellular carcinoma in Child-Pugh A patients treated with radical surgery: A retrospective observational study | Not included | Duplicate |
|  | Hepatocellular carcinoma amongst aboriginal and torres strait islander peoples of Australia | Not included | Duplicate |
|  | Hepatitis B vaccination status and associated factors among undergraduate students of Makerere University College of Health Sciences | Not included | Duplicate |
|  | Risk factors for underlying comorbidities and complications in patients with hepatitis B virus-related acute-on-chronic liver failure | Not included | Duplicate |
|  | Psychological profiles of excluded living liver donor candidates: An observational study | Not included | Duplicate |
|  | Prevalence of Hepatitis E Virus and Its Associated Outcomes among Pregnant Women in China | Not included | Duplicate |
|  | Clinical cure induced by pegylated interferon α-2b in the advantaged population of chronic hepatitis B virus infection: a retrospective cohort study | Not included | Duplicate |
|  | Prevalence and associated risk factors of Hepatitis B and Hepatitis C virus among volunteer blood donors in Arba Minch Blood Bank SNNPR, Ethiopia | Not included | Duplicate |
|  | Genetic variation in FCER1A predicts peginterferon alfa-2a-induced hepatitis B surface antigen clearance in East Asian patients with chronic hepatitis B | Not included | Duplicate |
|  | Cirrhosis and liver transplantation in patients co-infected with HIV and hepatitis B or C: an observational cohort study | Not included | Duplicate |
|  | Mutations in pre-core and basic core promoter regions of hepatitis B virus in chronic hepatitis B patients | Not included | Duplicate |
|  | Immune response pattern varies with the natural history of chronic hepatitis B | Not included | Duplicate |
|  | Role of interleukin-21 and interleukin-21 receptor polymorphisms in the treatment of HBeAg-positive chronic hepatitis B patients with peginterferon | Not included | Duplicate |
|  | The prevalence of hepatitis B virus infection in the United States in the era of vaccination | Not included | Duplicate |
|  | A Pilot Study of MicroRNAs Expression Profile in Serum and HBsAg Particles: Predictors of Therapeutic Vaccine Efficacy in Chronic Hepatitis B Patients | Not included | Duplicate |
|  | An epidemiological survey of HBV infection and low-level HBsAg in military camps in eastern China | Not included | Duplicate |
|  | Epidemiology and etiology of diffuse large B-cell lymphoma | Not included | Duplicate |
|  | Hepatitis B virus infection and related factors in hemodialysis patients in China–systematic review and meta-analysis | Not included | Duplicate |
|  | Prevalence of chronic obstructive pulmonary disease and associated risk factors in Uganda (FRESH AIR Uganda): a prospective cross-sectional observational study | Not included | Duplicate |
|  | Stopping nucleos(t)ide analogue treatment in Caucasian hepatitis B patients after HBeAg seroconversion is associated with high relapse rates and fatal outcomes | Not included | Duplicate |
|  | Caucasian Ethnicity, but Not Treatment Cessation is Associated with HBsAg Loss Following Nucleos(t)ide Analogue-Induced HBeAg Seroconversion | Not included | Duplicate |
|  | Prevalence an d factors associated with hepatitis B susceptibility among men who sex with men on HIV pre-exposure prophylaxis in Northeastern Brazil: a cross-sectional study | Not included | Duplicate |
|  | The Swiss STAR trial - an evaluation of target groups for sexually transmitted infection screening in the sub-sample of women | Not included | Duplicate |
|  | Poor adherence and low persistency rates for hepatocellular carcinoma surveillance in patients with chronic hepatitis B | Not included | Duplicate |
|  | Predictors of Hepatitis B Surface Antigen Titers two decades after vaccination in a cohort of students and post-graduates of the Medical School at the University of Palermo, Italy | Not included | Duplicate |
|  | High prevalence of hepatitis B virus and hepatitis D virus in the western Brazilian Amazon | Not included | Duplicate |
|  | Hepatitis B in Rondônia (Western amazon region, Brazil): descriptive analysis and spatial distribution | Not included | Duplicate |
|  | “I am still suffering:” The dilemma of multiple recoveries in the lives of methadone maintenance patients | Not included | Duplicate |
|  | Syphilis, human immunodeficiency virus, herpes genital and hepatitis B in a women's prison in Cochabamba, Bolivia: prevalence and risk factors | Not included | Duplicate |
|  | Switch from intravenous or intramuscular to subcutaneous hepatitis B immunoglobulin: effect on quality of life after liver transplantation | Not included | Duplicate |
|  | The value of APGA score, fibrosis index for diagnosing liver fibrosis in patients with chronic hepatitis B | Not included | Duplicate |
|  | Epidemiological patterns and risk factors associated with hepatitis B virus in Pakistani population | Not included | Duplicate |
|  | Protocol: Prospective observational study investigating the prevalence and clinical outcome of portopulmonary hypertension in Japanese patients with chronic liver disease | Not included | Duplicate |
|  | Chapter 26 - Hepatitis A Vaccines | Not included | Duplicate |
|  | Workplace hazards faced by nursing assistants in the United States: A focused literature review | Not included | Duplicate |
|  | Genotype Matters in Patients with Acute-on-chronic Liver Failure Due to Reactivation of Chronic Hepatitis B | Not included | Duplicate |
|  | Sex difference in the associations among risk factors with hepatitis B and C infections in a large Taiwanese population study | Not included | Duplicate |
|  | Protective effect of an improved immunization practice of mother-to-infant transmission of hepatitis B virus and risk factors associated with immunoprophylaxis failure | Not included | Duplicate |
|  | Prevalence and factors associated with hepatitis B immunization and infection among men who have sex with men in Beijing, China | Not included | Duplicate |
|  | Comparison of hepatitis B virus and hepatitis C virus prevalence and risk factors in a community-based study | Not included | Duplicate |
|  | Hepatitis C Virus Infection Associated With an Increased Risk of Deep Vein Thrombosis: A Population-Based Cohort Study | Not included | Duplicate |
|  | Low immediate postoperative platelet count is associated with hepatic insufficiency after hepatectomy | Not included | Duplicate |
|  | Clinical characteristics and risk factors of COVID-19 patients with chronic hepatitis B: a multi-center retrospective cohort study | Not included | Duplicate |
|  | A statistical analysis of the correlations among various types of clinical indexes for patients with chronic hepatitis B: A hospital-based study | Not included | Duplicate |
|  | Combined use of murine double minute-2 promoter methylation and serum AFP improves diagnostic efficiency in hepatitis B virus-related hepatocellular carcinoma | Not included | Duplicate |
|  | Abnormal IL-10 levels were related to alanine aminotransferase abnormalities during postpartum in HBeAg positive women with chronic hepatitis B | Not included | Duplicate |
|  | Gamma-glutamyl transpeptidase to platelet ratio index is a good noninvasive biomarker for predicting liver fibrosis in Chinese chronic hepatitis B patients | Not included | Duplicate |
|  | Risk factors for some tropical diseases in an African country | Not included | Duplicate |
|  | Epidemiology of hepatitis B virus infection in Bangladesh: prevalence among general population, risk groups and genotype distribution | Not included | Duplicate |
|  | Nosocomial infections: current situation in a resuscitation-unit | Not included | Duplicate |
|  | Hepatitis B virus infection | Not included | Duplicate |
|  | Does Nucleos(t)ide Analogues Treatment Affect Renal Function in Chronic Hepatitis B Patients Who Have Already Decreased eGFR? A Longitudinal Study | Not included | Duplicate |
|  | Seroepidemiology of the human herpesvirus 8 infection among people living with HIV in Taiwan, 2014-2018 | Not included | Duplicate |
|  | Sero-prevalence and risk factors for hepatitis B virus infection among the consumers of the alcoholic beverage, cheka in Konso zone, southwestern Ethiopia | Not included | Duplicate |
|  | Predictive factors for percutaneous and mucocutaneous exposure among healthcare workers in a developing country | Not included | Duplicate |
|  | Prevalence and predictors of hepatitis B virus coinfection in a United States cohort of hepatitis C virus‐infected patients | Not included | Duplicate |
|  | Superiority of tenofovir alafenamide fumarate over entecavir for serum HBsAg level reduction in patients with chronic HBV infection: A 144-week outcome study after switching of the nucleos(t)ide analog | Not included | Duplicate |
|  | The prevalence of hepatitis B and C viral infections among pregnant women | Not included | Duplicate |
|  | Prevalence and risk factors of hepatitis B virus transmission among children in Enugu, Nigeria | Not included | Duplicate |
|  | Hepatitis B virus infections and associated factors among pregnant women attending antenatal care clinic at Deder Hospital, Eastern Ethiopia | Not included | Duplicate |
|  | Cross-sectional study of chronic hepatitis B virus infection in Rwandan high-risk groups: unexpected findings on prevalence and its determinants | Not included | Duplicate |
|  | Screening a nation for hepatitis C virus elimination: a cross-sectional study on prevalence of hepatitis C and associated risk factors in the Rwandan general population | Not included | Duplicate |
|  | Role of quantitative hepatitis B surface antigen in predicting inactive carriers and HBsAg seroclearance in HBeAg-negative chronic hepatitis B patients | Not included | Duplicate |
|  | The role of Bcl-2 in hepatocarcinogenesis: Effects of overexpression on murine liver tumor development and hepatocyte cell cycle progression | Not included | Duplicate |
|  | CHAPTER 12 - Alcohol and Substance Abuse | Not included | Duplicate |
|  | Five‐year conditional survival for patients with hepatocellular carcinoma in Queensland, Australia | Not included | Duplicate |
|  | Admitted AIDS-associated Kaposi sarcoma patients: Indications for admission and predictors of mortality | Not included | Duplicate |
|  | Sustained viral response and relapse after discontinuation of oral antiviral drugs in HBeAg-positive patients with chronic hepatitis B infection | Not included | Duplicate |
|  | An observational study to evaluate the safety and efficacy of telbivudine in adults with chronic hepatitis B | Not included | Duplicate |
|  | Clinical outcome indicators in chronic hepatitis B and C: A primer for value-based medicine in hepatology | Not included | Duplicate |
|  | Patterns and co-occurrence of risk factors for hepatocellular carcinoma in four Asian American communities: a cross-sectional study | Not included | Duplicate |
|  | A Prospective Study Evaluating Changes in Histology, Clinical and Virologic Outcomes in HBV-HIV Co-infected Adults in North America | Not included | Duplicate |
|  | Long-term persistency of hepatitis B immunity: an observational cross-sectional study on medical students and resident doctors | Not included | Duplicate |
|  | Hepatitis B virus infection and factors associated with its acquisition among adults in a Lake Victoria HIV hyperendemic fishing community in Kyotera district, Uganda: a cross-sectional observation | Not included | Duplicate |
|  | Hepatitis B and C virus infection among 1.2 million persons with access to care: factors associated with testing and infection prevalence | Not included | Duplicate |
|  | Blood and virus detection on barber clippers | Not included | Duplicate |
|  | A new approach to prevent, diagnose, and treat hepatitis B in Africa | Not included | Duplicate |
|  | Infectious diseases prevalence, vaccination coverage, and diagnostic challenges in a population of internationally adopted children referred to a Tertiary Care Children's Hospital from 2009 to 2015 | Not included | Duplicate |
|  | Renal angina index in critically ill children as an applicable and reliable tool in the prediction of severe acute kidney injury: Two tertiary centers' prospective observational study from the Middle East | Not included | Duplicate |
|  | Associations Between Hepatitis B Virus Infection and Risk of All Cancer Types | Not included | Duplicate |
|  | Off-treatment virologic relapse and outcomes of re-treatment in chronic hepatitis B patients who achieved complete viral suppression with oral nucleos(t)ide analogs | Not included | Duplicate |
|  | Factors associated with sexually transmitted infections in sugarcane cutters: subsidies to caring for | Not included | Duplicate |
|  | Factors associated with immunoprophylaxis failure against vertical transmission of hepatitis B virus | Not included | Duplicate |
|  | Hospital personnel sero-protected against hepatitis B virus following an accelerated vaccination program | Not included | Duplicate |
|  | Predictors of liver disease progression in people living with HIV-HBV co-infection on antiretroviral therapy | Not included | Duplicate |
|  | High prevalence of hepatitis B-antibody loss and a case report of de novo hepatitis B virus infection in a child after living-donor liver transplantation | Not included | Duplicate |
|  | Sexually transmitted infections among patients attending a sexual assault centre: a cohort study from Oslo, Norway | Not included | Duplicate |
|  | Occupational risk perception in home health care workers | Not included | Duplicate |
|  | Post-vaccination anti-HBs testing among healthcare workers: More economical than post-exposure management for Hepatitis B | Not included | Duplicate |
|  | Blood donors and the supply of blood and blood products | Not included | Duplicate |
|  | Pro-Inflammatory Interleukin-18 is Associated with Hepatic Steatosis and Elevated Liver Enzymes in People with HIV Monoinfection | Not included | Duplicate |
|  | Sharps Injuries in Ambulatory Care: A Clinical Staff Perspective | Not included | Duplicate |
|  | Seroevidence for a high prevalence of subclinical infection with avian influenza A (H5N1) virus among workers in a live-poultry market in Indonesia | Not included | Duplicate |
|  | Effective therapeutic options for elderly patients with hepatocellular carcinoma: A nationwide cohort stud | Not included | Duplicate |
|  | Risk Factors for Renal Functional Decline in Chronic Hepatitis B Patients Receiving Oral Antiviral Agents | Not included | Duplicate |
|  | Prevalence and risk factors of hepatitis B virus, hepatitis C virus, and human immunodeficiency virus infections among drug addicts in Bangladesh | Not included | Duplicate |
|  | Seroprevalence of hepatitis B virus infection and associated factors among prison inmates in state of Mato Grosso do Sul, Brazil | Not included | Duplicate |
|  | Residual risk of mother-to-child transmission of hepatitis B virus infection despite timely birth-dose vaccination in Cameroon (ANRS 12303): a single-centre, longitudinal observational study | Not included | Duplicate |
|  | Dually Active HIV/HBV Antiretrovirals as Protection Against Incident Hepatitis B Infections: Potential for Prophylaxis | Not included | Duplicate |
|  | Practice and outcomes of neonatal resuscitation for newborns with birth asphyxia at Kakamega County General Hospital, Kenya: a direct observation study | Not included | Duplicate |
|  | Hepatitis B virus infection among medical waste handlers in Addis Ababa, Ethiopia | Not included | Duplicate |
|  | Information seeking behavior on hepatitis B virus, and its associated factors among pregnant women at teaching and specialized hospitals, Northwest Ethiopia: A cross-sectional study | Not included | Duplicate |
|  | Prevalence and risk factors of hepatic steatosis and its impact on liver injury in Chinese patients with chronic hepatitis B infection | Not included | Duplicate |
|  | Compliance and noncompliance of Universal Precautions among different groups of health care workers using the construct of the Health Belief Model: Implications for curriculum decision-making | Not included | Duplicate |
|  | Hepatitis B virus serosurvey and awareness of mother-to-child transmission among pregnant women in Shenyang, China: An observational study | Not included | Duplicate |
|  | The hepatitis B core antibody positive/hepatitis B surface antigen negative pattern is associated with the increased risk of intracranial atherosclerotic stenosis | Not included | Duplicate |
|  | Prevalence and factors associated with adverse drug reactions among heart failure patients hospitalized at Mbarara Regional Referral Hospital, Uganda | Not included | Duplicate |
|  | High seroprevalence and associated risk factors for hepatitis B virus infection among pregnant women living with HIV in Mtwara region, Tanzania | Not included | Duplicate |
|  | Acceptability and adherence to Isoniazid preventive therapy in HIV-infected patients clinically screened for latent tuberculosis in Dar es Salaam, Tanzania | Not included | Duplicate |
|  | Seroprevalence of hepatitis B virus infection and associated factors among healthcare workers in northern Tanzania | Not included | Duplicate |
|  | Seroprevalence of hepatitis B virus infection and associated factors among healthcare workers in northern Tanzania | Included | Duplicate |
|  | Multi-Omic Data Integration Allows Baseline Immune Signatures to Predict Hepatitis B Vaccine Response in a Small Cohort | Not included | Duplicate |
|  | Alcohol-Related Liver Disease Is Rarely Detected at Early Stages Compared With Liver Diseases of Other Etiologies Worldwide | Not included | Duplicate |
|  | Occupational safety and health in Spain | Not included | Duplicate |
|  | Infectious Diseases in Sub-Saharan Immigrants to Spain | Not included | Duplicate |
|  | Evaluating the appropriateness of chemotherapy in a low-resource cancer center in sub-Saharan Africa | Not included | Duplicate |
|  | Presentation, patterns of care, and outcomes of patients with prostate cancer in sub-Saharan Africa: A population-based registry study | Not included | Duplicate |
|  | Feasibility of the modified sequential organ function assessment score in a resource-constrained setting: a prospective observational study | Not included | Duplicate |
|  | Prevalence and risk factors associated with HIV/hepatitis B and HIV/hepatitis C co-infections among people who inject drugs in Mozambique | Not included | Duplicate |
|  | A prospective longitudinal study of psychosocial variables associated with the incidence of cancer among Seventh-day Adventists | Not included | Duplicate |
|  | Anti-TB drug concentrations and drug-associated toxicities among TB/HIV-coinfected patients | Not included | Duplicate |
|  | Occupational hazards | Not included | Duplicate |
|  | Estimations of worldwide prevalence of chronic hepatitis B virus infection: a systematic review of data published between 1965 and 2013 | Not included | Duplicate |
|  | Long-term follow-up of study participants from prophylactic HIV vaccine clinical trials in Africa | Not included | Duplicate |
|  | The Swiss STAR trial - an evaluation of target groups for sexually transmitted infection screening in the sub-sample of men | Not included | Duplicate |
|  | Using health-system-wide data to understand hepatitis B virus prophylaxis and reactivation outcomes in patients receiving rituximab | Not included | Duplicate |
|  | Poor clinical and virological outcome of nucleos(t)ide analogue monotherapy in HBV/HDV co-infected patients | Not included | Duplicate |
|  | A Study to Assess the Effectiveness of Structured Teaching Programme on Knowledge Regarding Universal Precautions and the Prevention of Blood Borne Infections Among the Final Year B. Sc. Nursing Students of Selected Nursing Colleges at Hassan, Karnataka | Not included | Duplicate |
|  | Impact of changing societal trends on the spread of infections in American and Canadian homes | Not included | Duplicate |
|  | The seroprevalence of the hepatitis B virus in Italian medical students after 3 decades since the introduction of universal vaccination | Not included | Duplicate |
|  | Correlates of infection and molecular characterization of blood-borne HIV, HCV, and HBV infections in HIV-1 infected inmates in Italy: An observational cross-sectional study | Not included | Duplicate |
|  | Prevalence and risk factors associated with hepatitis B and C in Nawabshah, Sindh, Pakistan | Not included | Duplicate |
|  | Percutaneous exposures among health care workers in a Greek tertiary hospital | Not included | Duplicate |
|  | Impact of Insulin Resistance on Therapeutic Response to Oral Treatment of Chronic Hepatitis C Virus Infection | Not included | Duplicate |
|  | Antiretroviral prophylaxis of health care workers at two urban medical centers | Not included | Duplicate |
|  | Patterns of antibiotic use, pathogens, and prediction of mortality in hospitalized neonates and young infants with sepsis: A global neonatal sepsis observational cohort study (NeoOBS) | Not included | Duplicate |
|  | Guidelines for the prevention of invasive mould diseases caused by filamentous fungi by the Spanish Society of Infectious Diseases and Clinical Microbiology (SEIMC) | Not included | Duplicate |
|  | Heterogeneity in neurocognitive change trajectories among people with HIV starting antiretroviral therapy in Rakai, Uganda | Not included | Duplicate |
|  | Early reduced liver graft survival in hepatitis C recipients identified by two combined genetic markers | Not included | Duplicate |
|  | Early reduced liver graft survival in hepatitis C recipients identified by two combined genetic markers | Not included | Duplicate |
|  | Seroprevalence of Hepatitis B Virus and Associated Factors Among Pregnant Women Attending Antenatal Care in Public Health Facilities in Jigjiga Town, Eastern Ethiopia | Not included | Duplicate |
|  | Career risk of hepatitis C virus infection among US emergency medical and public safety workers | Not included | Duplicate |
|  | Prevalence, risk factors, and outcomes for occult hepatitis B virus infection among HIV-infected patients | Not included | Duplicate |
|  | Reactive Blood Donor Notification; Their Responses And Perceptions: Experience From Southern Pakistan | Not included | Duplicate |
|  | Steatosis in chronic hepatitis B: prevalence and correlation with biochemical, histologic, viral, and metabolic parameters | Not included | Duplicate |
|  | Prevalence of hepatitis B virus, hepatitis C virus, and HIV infection among patients with newly diagnosed cancer from academic and community oncology practices | Not included | Duplicate |
|  | Nationwide retrospective study of hepatitis B virological response and liver stiffness improvement in 465 patients on nucleos(t)ide analogue | Not included | Duplicate |
|  | Hepatitis C virus infection in the Middle East and North Africa “MENA” region: injecting drug users (IDUs) is an under-investigated population | Not included | Duplicate |
|  | Novel point-of-care cytokine biomarker lateral flow test for the screening for sexually transmitted infections and bacterial vaginosis: study protocol of a multicentre multidisciplinary prospective observational clinical study to evaluate the performance and feasibility of the Genital InFlammation Test (GIFT) | Not included | Duplicate |
|  | Existing gaps and missed opportunities in delivering quality nutrition services in primary healthcare: a descriptive analysis of patient experience and provider competence in 11 low-income and middle-income countries | Not included | Duplicate |
|  | Capacity and quality of maternal and child health services delivery at the subnational primary healthcare level in relation to intermediate health outputs: a cross-sectional study of 12 low-income and middle-income countries | Not included | Duplicate |
|  | Waste Management With Special emphasis on occupational health and safety at selected healthcare establishments | Not included | Duplicate |
|  | Factors affecting the serological testing of cadaveric donor cornea | Not included | Duplicate |
|  | Identifying occupational hazards among healthcare workers in Australia and Bhutan | Not included | Duplicate |
|  | Chapter 30 - Occupational Health of Laboratory Animal Workers | Not included | Duplicate |
|  | Prevalence of hepatitis B and C viral infections in Pakistan: findings of a national survey appealing for effective prevention and control measures | Not included | Duplicate |
|  | Immunizations and oral health care providers | Not included | Duplicate |
|  | A positive-feedback loop between HBx and ALKBH5 promotes hepatocellular carcinogenesis | Not included | Duplicate |
|  | Serum Liver Fibrosis Markers in the Prognosis of Liver Cirrhosis: A Prospective Observational Study | Not included | Duplicate |
|  | Expansion of Stem Cell-Like CD4(+) Memory T Cells during Acute HIV-1 Infection Is Linked to Rapid Disease Progression | Not included | Duplicate |
|  | Cancer risk by social class and occupation: a survey of 109,000 cancer cases among Finns of working age | Not included | Duplicate |
|  | Improved Antibody Response to Three Additional Hepatitis B Vaccine Doses Following Primary Vaccination Failure in Patients with Inflammatory Bowel Disease | Not included | Duplicate |
|  | Sleep and antibody response to hepatitis B vaccination | Not included | Duplicate |
|  | Emerging and Re-emerging Pathogens and Diseases, and Health Consequences of a Changing Climate | Not included | Duplicate |
|  | Nurses and AIDS care: Occupational risk perception and the social construction of HIV | Not included | Duplicate |
|  | Liver steatosis in children with chronic hepatitis B and C: Prevalence, predictors, and impact on disease progression | Not included | Duplicate |
|  | Prevalence of hepatitis B virus (HBV) infection among Makerere University medical students | Not included | Duplicate |
|  | Suppl-1, M3: epidemiology of hepatitis B virus (HBV) and hepatitis C virus (HCV) related hepatocellular carcinoma | Not included | Duplicate |
|  | Hepatitis B virus infection and vaccine-induced immunity in Madrid (Spain) | Not included | Duplicate |
|  | Costs of needlestick injuries and subsequent hepatitis and HIV infection | Not included | Duplicate |
|  | To Study the Incidence, Predictive Factors and Clinical Outcome of Spontaneous Bacterial Peritonitis in Patients of Cirrhosis with Ascites | Not included | Duplicate |
|  | Delayed-type hypersensitivity and hepatitis B vaccine responses, in vivo markers of cellular and humoral immune function, and the risk of AIDS or death | Not included | Duplicate |
|  | Sharps injuries among hospital workers in Massachusetts, 2007 | Not included | Duplicate |
|  | Clinical features of HBsAg seroclearance in hepatitis B virus carriers in South Korea: A retrospective longitudinal study | Not included | Duplicate |
|  | Global patterns of hepatocellular carcinoma management from diagnosis to death: the BRIDGE Study | Not included | Duplicate |
|  | Assessing significant fibrosis using imaging-based elastography in chronic hepatitis B patients: Pilot study | Not included | Duplicate |
|  | Liver Transplantation from Brain-Dead Donors with Hepatitis B or C in South Korea: A 2014-2020 Korean Organ Transplantation Registry Data Analysis | Not included | Duplicate |
|  | Ten-Year Changes in the Hepatitis B Prevalence in the Birth Cohorts in Korea: Results From Nationally Representative Cross-Sectional Surveys | Not included | Duplicate |
|  | A Study to Assess the Knowledge and Attitude Regarding Occupational Exposure and Post Exposure Prophylaxis (PEP) for Hiv Among Student Nurses of Selected Nursing Institutes of Hubballi with a View to Develop an Information Guide Sheet | Not included | Duplicate |
|  | Observational and Genetic Associations of Body Mass Index and Hepatobiliary Diseases in a Relatively Lean Chinese Population | Not included | Duplicate |
|  | Prevalence, risk factors and virological profile of chronic hepatitis B virus infection in pregnant women in India | Not included | Duplicate |
|  | A novel system for predicting liver histopathology in patients with chronic hepatitis B | Not included | Duplicate |
|  | Hepatitis B immunization data of patients living with HIV/AIDS: a multi-centre study | Not included | Duplicate |
|  | Human immunodeficiency virus infection predictors and genetic diversity of hepatitis B virus and hepatitis C virus co-infections among drug users in three major Kenyan cities | Not included | Duplicate |
|  | Global epidemiology of hepatitis B virus infection: new estimates of age-specific HBsAg seroprevalence and endemicity | Not included | Duplicate |
|  | Exposure time to hepatitis B virus and associated risk factors among children in Edirne, Turkey | Not included | Duplicate |
|  | Hepatitis B virus infection among illegal drug users in Enugu State, Nigeria: prevalence, immune status, and related risk factors | Not included | Duplicate |
|  | Staff Nurse Education on Best Practices for Preventing Blood-Borne Pathogen Exposures | Not included | Duplicate |
|  | Evaluating vertical transmission of sexually transmitted infections to newborns | Not included | Duplicate |
|  | A systematic review and meta-analysis of the prevalence of hepatitis B virus infection among pregnant women in Nigeria | Not included | Duplicate |
|  | Knowledge, attitude, and risk factors of hepatitis B among waste scavengers in Lagos, Nigeria | Not included | Duplicate |
|  | A multi-centre cross-sectional study on hepatitis B vaccination coverage and associated factors among personnel working in health facilities in Kumasi, Ghana | Not included | Duplicate |
|  | The impact of HIV on hepatocellular cancer survival in Nigeria | Not included | Duplicate |
|  | Factors Influencing Hospital Cleaners’ Knowledge and Practices toward Hepatitis B prevention in Northern Province of Rwanda | Not included | Duplicate |
|  | A proposed predictive model for advanced fibrosis in patients with chronic hepatitis B and its validation | Not included | Duplicate |
|  | Hepatitis C prevalence and associated risk factors among individuals who are homeless and diagnosed with mental illness: At Home/Chez Soi Study, Vancouver, BC | Not included | Duplicate |
|  | Hepatitis A vaccines | Not included | Duplicate |
|  | Prevalence of hepatitis B virus infection in Nigeria, 2000-2013: A systematic review and meta-analysis | Not included | Duplicate |
|  | Seroprevalence of hepatitis B virus among antenatal clinic attendees in Gamawa Local Government Area, Bauchi State, Nigeria | Not included | Duplicate |
|  | Syphilis and HIV prevalence and associated factors to their co-infection, hepatitis B and hepatitis C viruses prevalence among female sex workers in Rwanda | Not included | Duplicate |
|  | Prevalence of Hepatitis B Virus (HBV) surface antigen and HBVassociated hepatocellular carcinoma in Kenyans of various ages | Not included | Duplicate |
|  | A nationwide cross-sectional review of in-hospital hepatitis B virus testing and disease burden estimation in Ghana, 2016 - 2021 | Not included | Duplicate |
|  | Studies on prevalence and risk factors for Hepatitis B Surface Antigen among secondary school students in north-central, Nigeria | Not included | Duplicate |
|  | Multidimensional Analysis of the Mother-to-child Transmission Risk Factors in Chronic Hepatitis B Virus Infection in Pregnant Women in Vietnam | Not included | Duplicate |
|  | Healthcare resource utilization and costs by disease severity in an insured national sample of US patients with chronic hepatitis B | Not included | Duplicate |
|  | Advancing Age and Comorbidity in a US Insured Population-Based Cohort of Patients With Chronic Hepatitis B | Not included | Duplicate |
|  | Hepatitis B‐related hepatocellular carcinoma: epidemiological characteristics and disease burden | Not included | Duplicate |
|  | An enormous hepatitis B virus‐related liver disease burden projected in Vietnam by 2025 | Not included | Duplicate |
|  | Hepatitis B in healthcare personnel: an update on the global landscape | Not included | Duplicate |
|  | Prevalence, infectivity and correlates of hepatitis B virus infection among pregnant women in a rural district of the Far North Region of Cameroon | Not included | Duplicate |
|  | Assessing risk behaviors and prevalence of sexually transmitted and blood-borne infections among female crack cocaine users in Salvador-Bahia, Brazil | Not included | Duplicate |
|  | Standard Precautions among HealthCare Workers in a Tertiary Health Facility in Enugu Metropolis, South-East Nigeria | Not included | Duplicate |
|  | HIV viraemia during hepatitis B vaccination shortens the duration of protective antibody levels | Not included | Duplicate |
|  | Viral load is a significant prognostic factor for hepatitis B virus‐associated hepatocellular carcinoma | Not included | Duplicate |
|  | Dual positivity of hepatitis B surface antigen and anti-hepatitis C virus antibody and associated factors among apparently healthy patients of Ekiti State, Nigeria | Not included | Duplicate |
|  | Hepatitis C virus infection and its associated factors among prisoners in a Nigerian prison | Not included | Duplicate |
|  | Early cranial ultrasound findings among infants with neonatal encephalopathy in Uganda: an observational study | Not included | Duplicate |
|  | Sero-prevalence of hepatitis B virus and associated factors among pregnant women in Gambella hospital, South Western Ethiopia: facility based cross-sectional study | Not included | Duplicate |
|  | Clinical characteristics and current management of hepatitis B and C in China | Not included | Duplicate |
|  | No contribution of lifestyle and environmental exposures to gender discrepancy of liver disease severity in chronic hepatitis b infection: Observations from the Haimen City cohort | Not included | Duplicate |
|  | Sero-Prevalence of Hepatitis B Virus Infection and Associated Factors Among Pregnant Women Attending Antenatal Care Services in Gedeo Zone, Southern Ethiopia | Not included | Duplicate |
|  | Effects of long-term antiretroviral therapy in reproductive-age women in sub-Saharan Africa (the PEPFAR PROMOTE study): a multi-country observational cohort study | Not included | Duplicate |
|  | Prevalence, genotype distribution and mutations of hepatitis B virus and the associated risk factors among pregnant women residing in the northern shores of Persian Gulf, Iran | Not included | Duplicate |
|  | Global Estimates on Biological Risks at Work | Not included | Duplicate |
|  | Telbivudine treatment of hepatitis B virus-infected pregnant women at different gestational stages for the prevention of mother-to-child transmission: Outcomes of telbivudine treatment during pregnancy | Not included | Duplicate |
|  | Occult Hepatitis B Virus Infection in Maintenance Hemodialysis Patients: Prevalence and Mutations in "a" Determinant | Not included | Duplicate |
|  | Prevalence of hepatitis B virus infection in Shenzhen, China, 2015–2018 | Not included | Duplicate |
|  | Identifying, preventing and controlling needle-stick injuries in Indonesia | Not included | Duplicate |
|  | Splash of body fluids among healthcare support staff in Ghana: a cross-sectional study | Not included | Duplicate |
|  | Physiological and psychosocial stressors among hemodialysis patients in the Buea Regional Hospital, Cameroon | Not included | Duplicate |
|  | Safety practice and associated factors among waste handlers in Governmental Hospitals in Addis Ababa, Ethiopia | Not included | Duplicate |
|  | Hepatitis B virus infection and associated risk factors among medical students in eastern Ethiopia | Not included | Duplicate |
|  | Risk factors associated with Hepatitis B virus infection among pregnant women attending public hospitals in Addis Ababa, Ethiopia | Not included | Duplicate |
|  | Clinicopathological analysis of patients with dual malignancies: A retrospective study | Not included | Duplicate |
|  | Prevalence and the associated factors of hepatitis B and hepatitis C viral infections among HIV-positive individuals in same-day antiretroviral therapy initiation program in Bangkok, Thailand | Not included | Duplicate |
|  | Prevalence of liver steatosis in patients with chronic hepatitis B: a study of associated factors and of relationship with fibrosis | Not included | Duplicate |
|  | Hepatitis B, hepatitis C, and mortality among HIV-positive individuals | Not included | Duplicate |
|  | Nephrotoxicity caused by oral antiviral agents in patients with chronic hepatitis B treated in a hospital for tropical diseases in Thailand | Not included | Duplicate |
|  | Sexual behaviour and practices among adolescent blood donors in Harare and Masvingo provinces, Zimbabwe | Not included | Duplicate |
|  | Thermal disinfection in hemodialysis using the A0 concept as dispenser | Not included | Duplicate |
|  | Foodborne Diseases: Overview of Biological Hazards and Foodborne Diseases | Not included | Duplicate |
|  | Guidelines for Preventing Infectious Complications among Hematopoietic Cell Transplantation Recipients: A Global Perspective | Not included | Duplicate |
|  | Barriers to hepatitis B vaccine coverage among healthcare workers in the Republic of Georgia: An international perspective | Not included | Duplicate |
|  | The impact of economic and social factors on the prevalence of hepatitis B in Turkey | Not included | Duplicate |
|  | Proliferative lupus nephritis in the absence of overt systemic lupus erythematosus: A historical study of 12 adult patients | Not included | Duplicate |
|  | Seroprevalence of hepatitis B and C virus infections and risk factors in Turkey: a fieldwork TURHEP study | Not included | Duplicate |
|  | Chronic liver disease detection and quantification | Not included | Duplicate |
|  | Inequalities in the use of secondary prevention of cardiovascular disease by socioeconomic status: evidence from the PURE observational study | Not included | Duplicate |
|  | Prevention of hepatitis B and C in the EU/EEA April 2024 | Not included | Duplicate |
|  | Sero-Prevalence of HBV and its Associated Factors Among Healthcare Providers in Public Health Facilities in Eastern Ethiopia | Not included | Duplicate |
|  | An Assessment of the Knowledge, Attitude, and Practice Toward Standard Precautions Among Health Workers From a Hospital in Northern Cyprus | Not included | Duplicate |
|  | Burden of Hepatitis-B Infections and Risk Factors among Healthcare Workers in Resource Limited Setting, Addis Ababa, Ethiopia | Included | Duplicate |
|  | Knowledge, attitude and prevalence of hepatitis B virus among healthcare workers: a cross- sectional, Hospital based study in Bamenda Health District, NWR, Cameroon | Not included | Duplicate |
|  | Seroprevalence of Hepatitis B Surface Antigen and Occupational Risk Factors Among Health Care Workers in Ekiti State, Nigeria | Not included | Duplicate |
|  | Epidemiology of viral hepatitis and HIV co-infection | Not included | Duplicate |
|  | The exposure rate to hepatitis B and C viruses among medical waste handlers in three government hospitals, southern Ethiopia | Included | Duplicate |
|  | Assessment of Knowledge, Attitude and Vaccination Status of Hepatitis B among Nursing Training Students in Ho, Ghana | Not included | Duplicate |
|  | Precautions for health care workers to avoid hepatitis B and C virus infection | Not included | Duplicate |
|  | Serological Evidence and Associated Factors of Hepatitis B Virus and Hepatitis C Virus Among Waste Handlers: A Cross-Sectional Study from Northeastern Ethiopia | Included | Duplicate |
|  | Systematic review and meta-analysis of HIV, HBV and HCV infection prevalence in Sudan | Not included | Duplicate |
|  | Hepatitis B Virus Infection and Associated Factors Among Adults in Southwest Ethiopia: Community-Based Cross-Sectional Study | Not included | Duplicate |
|  | Prevalence of hepatitis B virus markers in surgeons in Lagos, Nigeria | Included | Duplicate |
|  | Risk and management of blood-borne infections in health care worker | Not included | Duplicate |
|  | Hepatitis B vaccination status and needle-stick and sharps-related Injuries among medical school students in Nepal: a cross-sectional study | Not included | Duplicate |
|  | Seroprevalence of hepatitis B virus infection in Cameroon: a systematic review and meta-analysis | Not included | Duplicate |
|  | A historical perspective on the discovery and elucidation of the hepatitis B virus | Not included | Duplicate |
|  | Hepatitis B infection among health workers in Uganda: evidence of the need for health worker protection | Included | Duplicate |
|  | Hepatitis B Virus Blood Screening: Need for Reappraisal of Blood Safety Measures? | Not included | Duplicate |
|  | Coverage of hepatitis B vaccination in Swedish healthcare workers | Not included | Duplicate |
|  | Prevalence of hepatitis B surface antigen (HbsAg) among health professionals in public Hospitals in Addis Ababa, Ethiopia | Not included | Duplicate |
|  | Hepatitis B and human immunodeficiency virus co-infection among pregnant women in resource-limited high endemic setting, Addis Ababa, Ethiopia: implications for prevention and control measures | Not included | Duplicate |
|  | Knowledge, Attitude, and Practice towards Hepatitis B Infection Prevention and Screening among Indonesians | Not included | Duplicate |
|  | Epidemiological patterns of hepatitis B virus (HBV) in highly endemic areas | Not included | Duplicate |
|  | Seroprevalence of Hepatitis B virus infection and associated factors among health care workers in Southern Ghana | Included | Duplicate |
|  | Economic burden of hepatitis C-associated diseases: Europe, Asia Pacific, and the Americas | Not included | Duplicate |
|  | Seroprevalence of hepatitis B surface antigenaemia among healthcare worker in a private Nigerian tertiary health institution | Included | Duplicate |
|  | Hepatitis B Virus Infection in Pregnant Women, in Al Fashir Town, North Darfur State, Sudan | Not included | Duplicate |
|  | Epidemiologic and socioeconomic factors impacting hepatitis B virus and related hepatocellular carcinoma | Not included | Duplicate |
|  | Prevalence and correlates of hepatitis B and C seropositivity among health care workers in a semi urban setting in North Central Nigeria | Not included | Duplicate |
|  | Epidemiology of viral hepatitis in Somalia: Systematic review and meta-analysis study | Not included | Duplicate |
|  | The global burden of disease attributable to contaminated injections given in health care settings | Not included | Duplicate |
|  | Hepatitis B and C Viral Infection: Prevalence, Knowledge, Attitude, Practice, and Occupational Exposure among Healthcare Workers of Jimma University Medical Center, Southwest Ethiopia | Not included | Duplicate |
|  | Prevalence and predictors of hepatitis B virus (HBV) infection in east Africa: evidence from a systematic review and meta-analysis of epidemiological studies published from 2005 to 2020 | Not included | Duplicate |
|  | Hepatitis B and C seroprevalence among health care workers in a tertiary hospital in Rwanda | Included | Duplicate |
|  | Prevalence of hepatitis B virus infection and uptake of hepatitis B vaccine among healthcare workers, Makueni County, Kenya 2017 | Included | Duplicate |
|  | High prevalence of hepatitis B infections in Burkina Faso (1996-2017): a systematic review with meta-analysis of epidemiological studies | Not included | Duplicate |
|  | Impact of the national hepatitis B immunization program in China: a modeling study | Not included | Duplicate |
|  | Changing prevalence of chronic hepatitis B virus infection in China between 1973 and 2021: a systematic literature review and meta-analysis of 3740 studies and 231 million people | Not included | Duplicate |
|  | Assessing the prevalence of hepatitis B virus infection among health care workers in a referral hospital in Kisantu, Congo DR: a pilot study | Included | Duplicate |
|  | Seroprevalence of Hepatitis B Among Healthcare Workers in Asia and Africa and Its Association With Their Knowledge and Awareness: A Systematic Review and Meta-Analysis | Not included | Duplicate |
|  | Prevalence of hepatitis B virus and immunity status among healthcare workers in Beira City, Mozambique | Included | Duplicate |
|  | Seroprevalence and Knowledge of Hepatitis B Virus Infection Among Laboratory Workers at Kilimanjaro Christian Medical Centre in Moshi, Tanzania | Included | Duplicate |
|  | Hepatitis B virus in Lao dentists: A cross-sectional serological study | Not included | Duplicate |
|  | Cross sectional study of chronic hepatitis B prevalence among healthcare workers in an urban setting, Sierra Leone | Included | Duplicate |
|  | Distribution of hepatitis B virus genotypes among patients with chronic infection in Japan shifting toward an increase of genotype A | Not included | Duplicate |
|  | Hepatitis B Virus and Human Immunodeficiency Virus Infections among Health Care Workers in Some Health Care Centers in Benue State, Niger | Included | Duplicate |
|  | Hepatitis B virus infection status and associated factors among health care workers in selected hospitals in Kisumu County, Kenya: A cross-sectional study | Included | Duplicate |
|  | Prevalence of HBV, HCV and Associated Risk Factors Among Cleaners at Selected Public Health Centers in Addis Ababa, Ethiopia | Included | Duplicate |
|  | Hepatitis B virus infection and its associated factors among medical waste collectors at public health facilities in eastern Ethiopia: a facility-based cross-sectional study | Included | Duplicate |
|  | Prevalence of hepatitis B virus and associated risk factors among adults patients at Dessie referral and Kemise general hospitals in northeastern Ethiopia | Not included | Duplicate |
|  | Seroprevalence of hepatitis B surface antigen and anti HCV antibody and its associated risk factors among pregnant women attending maternity ward of Felege Hiwot Referral Hospital, northwest Ethiopia: a cross-sectional study | Not included | Duplicate |
|  | Prevalence of hepatitis B virus infection among health care workers in a tertiary hospital in Tanzania | Not included | Duplicate |
|  | Prevalence of hepatitis B virus infection in Nigeria, 2000-2013: a systematic review and meta-analysis | Not included | Duplicate |
|  | Seroprevalence of Hepatitis B and C among health care workers in Omdurman, Sudan | Included | Duplicate |
|  | Hepatitis B Prevalence, Knowledge and Occupational Factors among Health Care Workers in Fako Division, South West Region Cameroon | Not included | Duplicate |
|  | An enormous hepatitis B virus-related liver disease burden projected in Vietnam by 2025 | Not included | Duplicate |
|  | Hepatitis B in Healthcare Personnel: An Update on the Global Landscape | Not included | Duplicate |
|  | Hepatitis B infection is highly prevalent among patients presenting with jaundice in Kenya | Not included | Duplicate |
|  | Hepatitis B in Ghana: a systematic review & meta-analysis of prevalence studies (1995-2015) | Not included | Duplicate |
|  | Hepatitis B Fact sheet | Not included | Duplicate |
|  | The prevalence of serological markers for hepatitis B virus infection in Australian Naval personnel | Not included | Duplicate |
|  | The prevalence of serological markers for hepatitis B virus infection in Australian Naval personnel | Not included | Duplicate |
|  | Is universal HBV vaccination of healthcare workers a relevant strategy in developing endemic countries? The case of a university hospital in Niger | Not included | Duplicate |
|  | Prevalence and associated knowledge of hepatitis B infection among healthcare workers in Freetown, Sierra Leone | Included | Duplicate |
|  | Enzyme-linked immunosorbent assay for the quantitative/qualitative analysis of plant secondary metabolites | Not included | Duplicate |
|  | Occupational injury history and universal precautions awareness: a survey in Kabul hospital staff | Not included | Duplicate |
|  | Seroprevalence of Hepatitis B and C among Healthcare Workers in Dutse Metropolis Jigawa State, Nigeria | Included | Duplicate |
|  | Estimations of worldwide prevalence of chronic hepatitis B virus infection: a systematic review of data published between 1965 and 2013 | Not included | Duplicate |
|  | Seroprevalence of hepatitis B virus infection and associated factors among healthcare workers in northern Tanzania | Included | Duplicate |
|  | Impact of Training about Knowledge, Attitude and Practice of Hepatitis B infection and its Vaccination among Healthcare Professionals in a Tertiary Care Hospital of Northern Gujarat | Not included | Duplicate |
|  | A new approach to prevent, diagnose, and treat hepatitis B in Africa | Not included | Duplicate |
|  | Effectiveness of Hepatitis B Vaccination Campaign in Italy: Towards the Control of HBV Infection for the First Time in a European Country | Not included | Duplicate |
|  | Prevalence of central obesity and associated factors in Ethiopia: A systematic review and meta-analysis | Not included | Duplicate |
|  | Global, regional, and national incidence, prevalence, and years lived with disability for 301 acute and chronic diseases and injuries in 188 countries, 1990-2013: a systematic analysis for the Global Burden of Disease Study 2013 | Not included | Duplicate |
|  | Hepatitis | Not included | Duplicate |
|  | Health care worker safety | Not included | Duplicate |
|  | Global health sector strategy on Viral hepatitis 2016–2021 | Not included | Duplicate |
|  | Global Hepatitis Report | Not included | Duplicate |
|  | Overcoming fragility in Somalia to build a strong primary health care system | Not included | Duplicate |
|  | Occupational health: health workers | Not included | Duplicate |
|  | Hepatitis B | Not included | Duplicate |
|  | Global hepatitis report: Action for access in low- and middle-income countries | Not included | Duplicate |
|  | Sero-Prevalence and Associated Factors of Hepatitis B Virus Infection among Health Professionals in Adama Town, Oromia, Central Ethiopia | Included | Duplicate |
|  | Sero-prevalence of hepatitis B virus infection and associated factors among health care workers and medical waste handlers in primary hospitals of North-west Ethiopia | Included | Duplicate |
|  | Seroprevalence and risk factors for hepatitis B infection in an adult population in Northeast China | Not included | Duplicate |
|  | Let's Talk About B: Barriers to Hepatitis B Screening and Vaccination Among Asian and South Asian Immigrants in British Columbia | Not included | Duplicate |
|  | Sero-prevalence and risk factors for hepatitis B virus infection among health care workers in a tertiary hospital in Uganda | Included | Duplicate |
|  | Gamma-glutamyl transpeptidase-to-platelet ratio and the fibrosis-4 index in predicting hepatitis B virus-related hepatocellular carcinoma development in elderly chronic hepatitis B patients in China: A single-center retrospective study | Not included | Duplicate |
|  | Epidemiology of hepatitis B virus infection: results from a community-based study of 0.15 million residents in South China | Not included | Duplicate |
|  | Sero-prevalence and risk factors of hepatitis B virus and human immunodeficiency virus infection among pregnant women in Bahir Dar city, Northwest Ethiopia: a cross sectional study | Not included | Duplicate |
|  | Out of hospital cardiac arrest: when to resuscitat | Not included | Duplicate |
|  | Sharps injuries among health care workers in Cairo University Hospitals | Not included | Duplicate |
|  | Impact of Occupational Hazards on Healthcare Professionals' Mental (Psychological) Health: Evidence from Government-Owned Hospitals in Khulna, Bangladesh | Not included | Duplicate |
|  | Health Profiles of Newly Arrived Refugee Children in the United States, 2006-2012 | Not included | Duplicate |
|  | Cardiac health in patients with hepatitis B virus-related cirrhosis | Not included | Duplicate |
|  | Prevalence and Associated Factors of Human Papillomavirus Infection among Iraqi Women | Not included | Duplicate |
|  | Hepatitis C Virus Cascades of Care in the era of Direct-Acting Antiviral Therapy | Not included | Duplicate |
|  | Maternal health in China - challenges of the next decade | Not included | Duplicate |
|  | A study on prevention of bleeding complications using lusutrombopag for safe RFA in patients with hepatocellular carcinoma with low platelet counts: prospective observational study | Not included | Duplicate |
|  | Validation of the Korean Stroop Test in Diagnosis of Minimal Hepatic Encephalopathy | Not included | Duplicate |
|  | Seroprevalence and predictors of hepatitis B virus infection among pregnant women attending routine antenatal care in Arba Minch Hospital, South Ethiopia | Not included | Duplicate |
|  | Conservation and variability of hepatitis B core at different chronic hepatitis stages | Not included | Duplicate |
|  | Prevalence of Hepatitis B virus infection and its determinants among pregnant women in East Africa: Systematic review and Meta-analysis | Not included | Duplicate |
|  | HCC risk reduction with oral nucleos(t)ide analogues in patients with chronic hepatitis B: Not perfect, not good enough | Not included | Duplicate |
|  | Occupational exposures to blood and body fluids (BBFS) among health care workers and medical students in University of Gondar Hospital, Northwest of Ethiopia | Not included | Duplicate |
|  | Somatosensory Amplification, Anxiety, and Depression in Patients With Hepatitis B: Impact on Functionality | Not included | Duplicate |
|  | Health behaviors of Korean adults with hepatitis B: Findings of the 2016 Korean National Health and Nutrition Examination Survey | Not included | Duplicate |
|  | Prevalence of and risk factors for hepatitis C virus antibody among people who inject drugs in Cambodia: a national biological and behavioral survey | Not included | Duplicate |
|  | An updated systematic review and meta-analysis of the prevalence of hepatitis B virus in Ethiopia | Not included | Duplicate |
|  | Management of Takotsubo cardiomyopathy in non-academic hospitals in France: The Observational French SyndromEs of TakoTsubo (OFSETT) study | Not included | Duplicate |
|  | Clinical characteristics, prognosis, and surgical outcomes of patients with non-HBV and non-HCV related hepatocellular carcinoma: three-decade observational study | Not included | Duplicate |
|  | Characteristics of female sexual dysfunctions and obstetric complications related to female genital mutilation in Omdurman maternity hospital, Sudan | Not included | Duplicate |
|  | Prognostic value of immunoscore to identify mortality outcomes in adults with HBV-related primary hepatocellular carcinoma | Not included | Duplicate |
|  | The association of adverse outcomes in the mother with disease progression in offspring in families with clusters of hepatitis B virus infection and unfavorable prognoses in Northwest China | Not included | Duplicate |
|  | Hospital acquired infections and infection prevention practices in teaching hospitals in the Amhara regional state, Ethiopia | Not included | Duplicate |
|  | Sero-prevalence of hepatitis B virus infection and its risk factors among pregnant women attending antenatal clinic at Aminu Kano Teaching Hospital, Kano, Nigeria | Not included | Duplicate |
|  | Relationship of Treg/Th17 balance with HBeAg change in HBeAg-positive chronic hepatitis B patients receiving telbivudine antiviral treatment: A longitudinal observational study | Not included | Duplicate |
|  | Occupational exposures to blood and body fluids (BBFS) among health care workers and medical students in University of Gondar Hospital, Northwest of Ethiopia | Not included | Duplicate |
|  | Telbivudine and adefovir dipivoxil combination therapy improves renal function in patients with chronic hepatitis B: A STROBE-compliant article | Not included | Duplicate |
|  | Outcomes of Glucocorticoid Treatment in HBV Associated Acute-on-Chronic Liver Failure Patients: A Retrospective Observational Study | Not included | Duplicate |
|  | Multimodal Ultrasound Model Based on the Left Gastric Vein in B-Viral Cirrhosis: Noninvasive Prediction of Esophageal Varices | Not included | Duplicate |
|  | Multiple Primary Malignancies in Patients With Hepatocellular Carcinoma: A Largest Series With 26-Year Follow-Up | Not included | Duplicate |
|  | Expression profiles of transcription factors for special CD4+ T-cell subsets in peripheral blood mononuclear cells from patients with hepatitis B virus infection | Not included | Duplicate |
|  | Hepatitis B virus-associated hepatocellular carcinoma | Not included | Duplicate |
|  | HBV pgRNA profiles in Chinese HIV/HBV coinfected patients under pre- and posttreatment: a multicentre observational cohort study | Not included | Duplicate |
|  | Population-Based Multicentric Survey of Hepatitis B Infection and Risk Factors in the North, South, and Southeast Regions of Brazil, 10-20 Years After the Beginning of Vaccination | Not included | Duplicate |
|  | Analysis of serum hepatitis B virus RNA levels among HBsAg and HBsAb copositive patients and its correlation with HBV DNA | Not included | Duplicate |
|  | Global Burden and Trends of Primary Liver Cancer Attributable to Comorbid Type 2 Diabetes Mellitus Among People Living with Hepatitis B: An Observational Trend Study from 1990 to 2019 | Not included | Duplicate |
|  | Systemic immune-inflammation index predicts postoperative acute kidney injury in hepatocellular carcinoma patients after hepatectomy | Not included | Duplicate |
|  | The imported infections among foreign travelers in China: an observational study | Not included | Duplicate |
|  | An updated systematic review and meta-analysis of the prevalence of hepatitis B virus in Ethiopia | Not included | Duplicate |
|  | Hospital acquired infections and infection prevention practices in teaching hospitals in the Amhara regional state, Ethiopia | Not included | Duplicate |
|  | 2022 International Consensus on Cardiopulmonary Resuscitation and Emergency Cardiovascular Care Science With Treatment Recommendations: Summary From the Basic Life Support; Advanced Life Support; Pediatric Life Support; Neonatal Life Support; Education, Implementation, and Teams; and First Aid Task Forces | Not included | Duplicate |
|  | Mercury Exposure and Poor Nutritional Status Reduce Response to Six Expanded Program on Immunization Vaccines in Children: An Observational Cohort Study of Communities Affected by Gold Mining in the Peruvian Amazon | Not included | Duplicate |
|  | Maternal exposure to carbon monoxide and fine particulate matter during pregnancy in an urban Tanzanian cohort | Not included | Duplicate |
|  | Sero-prevalence of hepatitis B virus infection and its risk factors among pregnant women attending antenatal clinic at Aminu Kano Teaching Hospital, Kano, Nigeria | Not included | Duplicate |
|  | Hepatitis B virus-associated hepatocellular carcinoma | Not included | Duplicate |
|  | Add-on pegylated interferon augments hepatitis B surface antigen clearance vs continuous nucleos(t)ide analog monotherapy in Chinese patients with chronic hepatitis B and hepatitis B surface antigen ≤ 1500 IU/mL: An observational study | Not included | Duplicate |
|  | Pneumococcal and influenza vaccination coverage among at-risk adults: A 5-year French national observational study | Not included | Duplicate |
|  | A modified MELD model for Chinese pre-ACLF and ACLF patients and it reveals poor prognosis in pre-ACLF patients | Not included | Duplicate |
|  | Analysis of serum hepatitis B virus RNA levels among HBsAg and HBsAb copositive patients and its correlation with HBV DNA | Not included | Duplicate |
|  | Statins improve outcomes of nonsurgical curative treatments in hepatocellular carcinoma patients | Not included | Duplicate |
|  | Genome-wide study of salivary microRNAs as potential noninvasive biomarkers for detection of nasopharyngeal carcinoma | Not included | Duplicate |
|  | Comparison of the efficacy and safety of entecavir and tenofovir in nucleos(t)ide analogue-naive chronic hepatitis B patients with high viraemia: a retrospective cohort study | Not included | Duplicate |
|  | Multicenter study of skin rashes and hepatotoxicity in antiretroviral-naïve HIV-positive patients receiving non-nucleoside reverse-transcriptase inhibitor plus nucleoside reverse-transcriptase inhibitors in Taiwan | Not included | Duplicate |
|  | Methylation status of the stimulator of interferon genes promoter in patients with chronic hepatitis B | Not included | Duplicate |
|  | Postnatal infection surveillance by telephone in Dar es Salaam, Tanzania: An observational cohort study | Not included | Duplicate |
|  | MRI findings in people with epilepsy and nodding syndrome in an area endemic for onchocerciasis: an observational study | Not included | Duplicate |
|  | Bacterial pathogenesis: a molecular approach | Not included | Duplicate |
|  | Clinical features of treatment-naive patients with hepatitis B virus infection: A community-based survey from high- and intermediate-hepatitis B endemicity regions in Southeast China | Not included | Duplicate |
|  | Rituximab plus chemotherapy as first-line treatment in Chinese patients with diffuse large B-cell lymphoma in routine practice: a prospective, multicentre, non-interventional study | Not included | Duplicate |
|  | Albumin-bilirubin and platelet-albumin-bilirubin grades for hepatitis B-associated hepatocellular carcinoma in Child-Pugh A patients treated with radical surgery: A retrospective observational study | Not included | Duplicate |
|  | Hepatocellular carcinoma amongst aboriginal and torres strait islander peoples of Australia | Not included | Duplicate |
|  | Hepatitis B vaccination status and associated factors among undergraduate students of Makerere University College of Health Sciences | Not included | Duplicate |
|  | Risk factors for underlying comorbidities and complications in patients with hepatitis B virus-related acute-on-chronic liver failure | Not included | Duplicate |
|  | Psychological profiles of excluded living liver donor candidates: An observational study | Not included | Duplicate |
|  | Prevalence of Hepatitis E Virus and Its Associated Outcomes among Pregnant Women in China | Not included | Duplicate |
|  | Clinical cure induced by pegylated interferon α-2b in the advantaged population of chronic hepatitis B virus infection: a retrospective cohort study | Not included | Duplicate |
|  | Prevalence and associated risk factors of Hepatitis B and Hepatitis C virus among volunteer blood donors in Arba Minch Blood Bank SNNPR, Ethiopia | Not included | Duplicate |
|  | Genetic variation in FCER1A predicts peginterferon alfa-2a-induced hepatitis B surface antigen clearance in East Asian patients with chronic hepatitis B | Not included | Duplicate |
|  | Cirrhosis and liver transplantation in patients co-infected with HIV and hepatitis B or C: an observational cohort study | Not included | Duplicate |
|  | Mutations in pre-core and basic core promoter regions of hepatitis B virus in chronic hepatitis B patients | Not included | Duplicate |
|  | Immune response pattern varies with the natural history of chronic hepatitis B | Not included | Duplicate |
|  | Role of interleukin-21 and interleukin-21 receptor polymorphisms in the treatment of HBeAg-positive chronic hepatitis B patients with peginterferon | Not included | Duplicate |
|  | The prevalence of hepatitis B virus infection in the United States in the era of vaccination | Not included | Duplicate |
|  | A Pilot Study of MicroRNAs Expression Profile in Serum and HBsAg Particles: Predictors of Therapeutic Vaccine Efficacy in Chronic Hepatitis B Patients | Not included | Duplicate |
|  | An epidemiological survey of HBV infection and low-level HBsAg in military camps in eastern China | Not included | Duplicate |
|  | Epidemiology and etiology of diffuse large B-cell lymphoma | Not included | Duplicate |
|  | Hepatitis B virus infection and related factors in hemodialysis patients in China–systematic review and meta-analysis | Not included | Duplicate |
|  | Prevalence of chronic obstructive pulmonary disease and associated risk factors in Uganda (FRESH AIR Uganda): a prospective cross-sectional observational study | Not included | Duplicate |
|  | Stopping nucleos(t)ide analogue treatment in Caucasian hepatitis B patients after HBeAg seroconversion is associated with high relapse rates and fatal outcomes | Not included | Duplicate |
|  | Caucasian Ethnicity, but Not Treatment Cessation is Associated with HBsAg Loss Following Nucleos(t)ide Analogue-Induced HBeAg Seroconversion | Not included | Duplicate |
|  | Prevalence an d factors associated with hepatitis B susceptibility among men who sex with men on HIV pre-exposure prophylaxis in Northeastern Brazil: a cross-sectional study | Not included | Duplicate |
|  | The Swiss STAR trial - an evaluation of target groups for sexually transmitted infection screening in the sub-sample of women | Not included | Duplicate |
|  | Poor adherence and low persistency rates for hepatocellular carcinoma surveillance in patients with chronic hepatitis B | Not included | Duplicate |
|  | Predictors of Hepatitis B Surface Antigen Titers two decades after vaccination in a cohort of students and post-graduates of the Medical School at the University of Palermo, Italy | Not included | Duplicate |
|  | High prevalence of hepatitis B virus and hepatitis D virus in the western Brazilian Amazon | Not included | Duplicate |
|  | Hepatitis B in Rondônia (Western amazon region, Brazil): descriptive analysis and spatial distribution | Not included | Duplicate |
|  | “I am still suffering:” The dilemma of multiple recoveries in the lives of methadone maintenance patients | Not included | Duplicate |
|  | Syphilis, human immunodeficiency virus, herpes genital and hepatitis B in a women's prison in Cochabamba, Bolivia: prevalence and risk factors | Not included | Duplicate |
|  | Switch from intravenous or intramuscular to subcutaneous hepatitis B immunoglobulin: effect on quality of life after liver transplantation | Not included | Duplicate |
|  | The value of APGA score, fibrosis index for diagnosing liver fibrosis in patients with chronic hepatitis B | Not included | Duplicate |
|  | Epidemiological patterns and risk factors associated with hepatitis B virus in Pakistani population | Not included | Duplicate |
|  | Protocol: Prospective observational study investigating the prevalence and clinical outcome of portopulmonary hypertension in Japanese patients with chronic liver disease | Not included | Duplicate |
|  | Chapter 26 - Hepatitis A Vaccines | Not included | Duplicate |
|  | Workplace hazards faced by nursing assistants in the United States: A focused literature review | Not included | Duplicate |
|  | Genotype Matters in Patients with Acute-on-chronic Liver Failure Due to Reactivation of Chronic Hepatitis B | Not included | Duplicate |
|  | Sex difference in the associations among risk factors with hepatitis B and C infections in a large Taiwanese population study | Not included | Duplicate |
|  | Protective effect of an improved immunization practice of mother-to-infant transmission of hepatitis B virus and risk factors associated with immunoprophylaxis failure | Not included | Duplicate |
|  | Prevalence and factors associated with hepatitis B immunization and infection among men who have sex with men in Beijing, China | Not included | Duplicate |
|  | Comparison of hepatitis B virus and hepatitis C virus prevalence and risk factors in a community-based study | Not included | Duplicate |
|  | Hepatitis C Virus Infection Associated With an Increased Risk of Deep Vein Thrombosis: A Population-Based Cohort Study | Not included | Duplicate |
|  | Low immediate postoperative platelet count is associated with hepatic insufficiency after hepatectomy | Not included | Duplicate |
|  | Clinical characteristics and risk factors of COVID-19 patients with chronic hepatitis B: a multi-center retrospective cohort study | Not included | Duplicate |
|  | A statistical analysis of the correlations among various types of clinical indexes for patients with chronic hepatitis B: A hospital-based study | Not included | Duplicate |
|  | Combined use of murine double minute-2 promoter methylation and serum AFP improves diagnostic efficiency in hepatitis B virus-related hepatocellular carcinoma | Not included | Duplicate |
|  | Abnormal IL-10 levels were related to alanine aminotransferase abnormalities during postpartum in HBeAg positive women with chronic hepatitis B | Not included | Duplicate |
|  | Gamma-glutamyl transpeptidase to platelet ratio index is a good noninvasive biomarker for predicting liver fibrosis in Chinese chronic hepatitis B patients | Not included | Duplicate |
|  | Risk factors for some tropical diseases in an African country | Not included | Duplicate |
|  | Epidemiology of hepatitis B virus infection in Bangladesh: prevalence among general population, risk groups and genotype distribution | Not included | Duplicate |
|  | Nosocomial infections: current situation in a resuscitation-unit | Not included | Duplicate |
|  | Hepatitis B virus infection | Not included | Duplicate |
|  | Does Nucleos(t)ide Analogues Treatment Affect Renal Function in Chronic Hepatitis B Patients Who Have Already Decreased eGFR? A Longitudinal Study | Not included | Duplicate |
|  | Seroepidemiology of the human herpesvirus 8 infection among people living with HIV in Taiwan, 2014-2018 | Not included | Duplicate |
|  | Sero-prevalence and risk factors for hepatitis B virus infection among the consumers of the alcoholic beverage, cheka in Konso zone, southwestern Ethiopia | Not included | Duplicate |
|  | Predictive factors for percutaneous and mucocutaneous exposure among healthcare workers in a developing country | Not included | Duplicate |
|  | Prevalence and predictors of hepatitis B virus coinfection in a United States cohort of hepatitis C virus‐infected patients | Not included | Duplicate |
|  | Superiority of tenofovir alafenamide fumarate over entecavir for serum HBsAg level reduction in patients with chronic HBV infection: A 144-week outcome study after switching of the nucleos(t)ide analog | Not included | Duplicate |
|  | The prevalence of hepatitis B and C viral infections among pregnant women | Not included | Duplicate |
|  | Prevalence and risk factors of hepatitis B virus transmission among children in Enugu, Nigeria | Not included | Duplicate |
|  | Hepatitis B virus infections and associated factors among pregnant women attending antenatal care clinic at Deder Hospital, Eastern Ethiopia | Not included | Duplicate |
|  | Cross-sectional study of chronic hepatitis B virus infection in Rwandan high-risk groups: unexpected findings on prevalence and its determinants | Not included | Duplicate |
|  | Screening a nation for hepatitis C virus elimination: a cross-sectional study on prevalence of hepatitis C and associated risk factors in the Rwandan general population | Not included | Duplicate |
|  | Role of quantitative hepatitis B surface antigen in predicting inactive carriers and HBsAg seroclearance in HBeAg-negative chronic hepatitis B patients | Not included | Duplicate |
|  | The role of Bcl-2 in hepatocarcinogenesis: Effects of overexpression on murine liver tumor development and hepatocyte cell cycle progression | Not included | Duplicate |
|  | CHAPTER 12 - Alcohol and Substance Abuse | Not included | Duplicate |
|  | Five‐year conditional survival for patients with hepatocellular carcinoma in Queensland, Australia | Not included | Duplicate |
|  | Admitted AIDS-associated Kaposi sarcoma patients: Indications for admission and predictors of mortality | Not included | Duplicate |
|  | Sustained viral response and relapse after discontinuation of oral antiviral drugs in HBeAg-positive patients with chronic hepatitis B infection | Not included | Duplicate |
|  | An observational study to evaluate the safety and efficacy of telbivudine in adults with chronic hepatitis B | Not included | Duplicate |
|  | Clinical outcome indicators in chronic hepatitis B and C: A primer for value-based medicine in hepatology | Not included | Duplicate |
|  | Patterns and co-occurrence of risk factors for hepatocellular carcinoma in four Asian American communities: a cross-sectional study | Not included | Duplicate |
|  | A Prospective Study Evaluating Changes in Histology, Clinical and Virologic Outcomes in HBV-HIV Co-infected Adults in North America | Not included | Duplicate |
|  | Long-term persistency of hepatitis B immunity: an observational cross-sectional study on medical students and resident doctors | Not included | Duplicate |
|  | Hepatitis B virus infection and factors associated with its acquisition among adults in a Lake Victoria HIV hyperendemic fishing community in Kyotera district, Uganda: a cross-sectional observation | Not included | Duplicate |
|  | Hepatitis B and C virus infection among 1.2 million persons with access to care: factors associated with testing and infection prevalence | Not included | Duplicate |
|  | Blood and virus detection on barber clippers | Not included | Duplicate |
|  | A new approach to prevent, diagnose, and treat hepatitis B in Africa | Not included | Duplicate |
|  | Infectious diseases prevalence, vaccination coverage, and diagnostic challenges in a population of internationally adopted children referred to a Tertiary Care Children's Hospital from 2009 to 2015 | Not included | Duplicate |
|  | Renal angina index in critically ill children as an applicable and reliable tool in the prediction of severe acute kidney injury: Two tertiary centers' prospective observational study from the Middle East | Not included | Duplicate |
|  | Associations Between Hepatitis B Virus Infection and Risk of All Cancer Types | Not included | Duplicate |
|  | Off-treatment virologic relapse and outcomes of re-treatment in chronic hepatitis B patients who achieved complete viral suppression with oral nucleos(t)ide analogs | Not included | Duplicate |
|  | Factors associated with sexually transmitted infections in sugarcane cutters: subsidies to caring for | Not included | Duplicate |
|  | Factors associated with immunoprophylaxis failure against vertical transmission of hepatitis B virus | Not included | Duplicate |
|  | Hospital personnel sero-protected against hepatitis B virus following an accelerated vaccination program | Not included | Duplicate |
|  | Predictors of liver disease progression in people living with HIV-HBV co-infection on antiretroviral therapy | Not included | Duplicate |
|  | High prevalence of hepatitis B-antibody loss and a case report of de novo hepatitis B virus infection in a child after living-donor liver transplantation | Not included | Duplicate |
|  | Sexually transmitted infections among patients attending a sexual assault centre: a cohort study from Oslo, Norway | Not included | Duplicate |
|  | Occupational risk perception in home health care workers | Not included | Duplicate |
|  | Post-vaccination anti-HBs testing among healthcare workers: More economical than post-exposure management for Hepatitis B | Not included | Duplicate |
|  | Blood donors and the supply of blood and blood products | Not included | Duplicate |
|  | Pro-Inflammatory Interleukin-18 is Associated with Hepatic Steatosis and Elevated Liver Enzymes in People with HIV Monoinfection | Not included | Duplicate |
|  | Sharps Injuries in Ambulatory Care: A Clinical Staff Perspective | Not included | Duplicate |
|  | Seroevidence for a high prevalence of subclinical infection with avian influenza A (H5N1) virus among workers in a live-poultry market in Indonesia | Not included | Duplicate |
|  | Effective therapeutic options for elderly patients with hepatocellular carcinoma: A nationwide cohort stud | Not included | Duplicate |
|  | Risk Factors for Renal Functional Decline in Chronic Hepatitis B Patients Receiving Oral Antiviral Agents | Not included | Duplicate |
|  | Prevalence and risk factors of hepatitis B virus, hepatitis C virus, and human immunodeficiency virus infections among drug addicts in Bangladesh | Not included | Duplicate |
|  | Seroprevalence of hepatitis B virus infection and associated factors among prison inmates in state of Mato Grosso do Sul, Brazil | Not included | Duplicate |
|  | Residual risk of mother-to-child transmission of hepatitis B virus infection despite timely birth-dose vaccination in Cameroon (ANRS 12303): a single-centre, longitudinal observational study | Not included | Duplicate |
|  | Dually Active HIV/HBV Antiretrovirals as Protection Against Incident Hepatitis B Infections: Potential for Prophylaxis | Not included | Duplicate |
|  | Practice and outcomes of neonatal resuscitation for newborns with birth asphyxia at Kakamega County General Hospital, Kenya: a direct observation study | Not included | Duplicate |
|  | Hepatitis B virus infection among medical waste handlers in Addis Ababa, Ethiopia | Not included | Duplicate |
|  | Information seeking behavior on hepatitis B virus, and its associated factors among pregnant women at teaching and specialized hospitals, Northwest Ethiopia: A cross-sectional study | Not included | Duplicate |
|  | Prevalence and risk factors of hepatic steatosis and its impact on liver injury in Chinese patients with chronic hepatitis B infection | Not included | Duplicate |
|  | Compliance and noncompliance of Universal Precautions among different groups of health care workers using the construct of the Health Belief Model: Implications for curriculum decision-making | Not included | Duplicate |
|  | Hepatitis B virus serosurvey and awareness of mother-to-child transmission among pregnant women in Shenyang, China: An observational study | Not included | Duplicate |
|  | The hepatitis B core antibody positive/hepatitis B surface antigen negative pattern is associated with the increased risk of intracranial atherosclerotic stenosis | Not included | Duplicate |
|  | Prevalence and factors associated with adverse drug reactions among heart failure patients hospitalized at Mbarara Regional Referral Hospital, Uganda | Not included | Duplicate |
|  | High seroprevalence and associated risk factors for hepatitis B virus infection among pregnant women living with HIV in Mtwara region, Tanzania | Not included | Duplicate |
|  | Acceptability and adherence to Isoniazid preventive therapy in HIV-infected patients clinically screened for latent tuberculosis in Dar es Salaam, Tanzania | Not included | Duplicate |
|  | Seroprevalence of hepatitis B virus infection and associated factors among healthcare workers in northern Tanzania | Not included | Duplicate |
|  | Seroprevalence of hepatitis B virus infection and associated factors among healthcare workers in northern Tanzania | Included | Duplicate |
|  | Multi-Omic Data Integration Allows Baseline Immune Signatures to Predict Hepatitis B Vaccine Response in a Small Cohort | Not included | Duplicate |
|  | Alcohol-Related Liver Disease Is Rarely Detected at Early Stages Compared With Liver Diseases of Other Etiologies Worldwide | Not included | Duplicate |
|  | Occupational safety and health in Spain | Not included | Duplicate |
|  | Infectious Diseases in Sub-Saharan Immigrants to Spain | Not included | Duplicate |
|  | Evaluating the appropriateness of chemotherapy in a low-resource cancer center in sub-Saharan Africa | Not included | Duplicate |
|  | Presentation, patterns of care, and outcomes of patients with prostate cancer in sub-Saharan Africa: A population-based registry study | Not included | Duplicate |
|  | Feasibility of the modified sequential organ function assessment score in a resource-constrained setting: a prospective observational study | Not included | Duplicate |
|  | Prevalence and risk factors associated with HIV/hepatitis B and HIV/hepatitis C co-infections among people who inject drugs in Mozambique | Not included | Duplicate |
|  | A prospective longitudinal study of psychosocial variables associated with the incidence of cancer among Seventh-day Adventists | Not included | Duplicate |
|  | Anti-TB drug concentrations and drug-associated toxicities among TB/HIV-coinfected patients | Not included | Duplicate |
|  | Occupational hazards | Not included | Duplicate |
|  | Estimations of worldwide prevalence of chronic hepatitis B virus infection: a systematic review of data published between 1965 and 2013 | Not included | Duplicate |
|  | Long-term follow-up of study participants from prophylactic HIV vaccine clinical trials in Africa | Not included | Duplicate |
|  | The Swiss STAR trial - an evaluation of target groups for sexually transmitted infection screening in the sub-sample of men | Not included | Duplicate |
|  | Using health-system-wide data to understand hepatitis B virus prophylaxis and reactivation outcomes in patients receiving rituximab | Not included | Duplicate |
|  | Poor clinical and virological outcome of nucleos(t)ide analogue monotherapy in HBV/HDV co-infected patients | Not included | Duplicate |
|  | A Study to Assess the Effectiveness of Structured Teaching Programme on Knowledge Regarding Universal Precautions and the Prevention of Blood Borne Infections Among the Final Year B. Sc. Nursing Students of Selected Nursing Colleges at Hassan, Karnataka | Not included | Duplicate |
|  | Impact of changing societal trends on the spread of infections in American and Canadian homes | Not included | Duplicate |
|  | The seroprevalence of the hepatitis B virus in Italian medical students after 3 decades since the introduction of universal vaccination | Not included | Duplicate |
|  | Correlates of infection and molecular characterization of blood-borne HIV, HCV, and HBV infections in HIV-1 infected inmates in Italy: An observational cross-sectional study | Not included | Duplicate |
|  | Prevalence and risk factors associated with hepatitis B and C in Nawabshah, Sindh, Pakistan | Not included | Duplicate |
|  | Percutaneous exposures among health care workers in a Greek tertiary hospital | Not included | Duplicate |
|  | Impact of Insulin Resistance on Therapeutic Response to Oral Treatment of Chronic Hepatitis C Virus Infection | Not included | Duplicate |
|  | Antiretroviral prophylaxis of health care workers at two urban medical centers | Not included | Duplicate |
|  | Patterns of antibiotic use, pathogens, and prediction of mortality in hospitalized neonates and young infants with sepsis: A global neonatal sepsis observational cohort study (NeoOBS) | Not included | Duplicate |
|  | Guidelines for the prevention of invasive mould diseases caused by filamentous fungi by the Spanish Society of Infectious Diseases and Clinical Microbiology (SEIMC) | Not included | Duplicate |
|  | Heterogeneity in neurocognitive change trajectories among people with HIV starting antiretroviral therapy in Rakai, Uganda | Not included | Duplicate |
|  | Early reduced liver graft survival in hepatitis C recipients identified by two combined genetic markers | Not included | Duplicate |
|  | Early reduced liver graft survival in hepatitis C recipients identified by two combined genetic markers | Not included | Duplicate |
|  | Seroprevalence of Hepatitis B Virus and Associated Factors Among Pregnant Women Attending Antenatal Care in Public Health Facilities in Jigjiga Town, Eastern Ethiopia | Not included | Duplicate |
|  | Career risk of hepatitis C virus infection among US emergency medical and public safety workers | Not included | Duplicate |
|  | Prevalence, risk factors, and outcomes for occult hepatitis B virus infection among HIV-infected patients | Not included | Duplicate |
|  | Reactive Blood Donor Notification; Their Responses And Perceptions: Experience From Southern Pakistan | Not included | Duplicate |
|  | Steatosis in chronic hepatitis B: prevalence and correlation with biochemical, histologic, viral, and metabolic parameters | Not included | Duplicate |
|  | Prevalence of hepatitis B virus, hepatitis C virus, and HIV infection among patients with newly diagnosed cancer from academic and community oncology practices | Not included | Duplicate |
|  | Nationwide retrospective study of hepatitis B virological response and liver stiffness improvement in 465 patients on nucleos(t)ide analogue | Not included | Duplicate |
|  | Hepatitis C virus infection in the Middle East and North Africa “MENA” region: injecting drug users (IDUs) is an under-investigated population | Not included | Duplicate |
|  | Novel point-of-care cytokine biomarker lateral flow test for the screening for sexually transmitted infections and bacterial vaginosis: study protocol of a multicentre multidisciplinary prospective observational clinical study to evaluate the performance and feasibility of the Genital InFlammation Test (GIFT) | Not included | Duplicate |
|  | Existing gaps and missed opportunities in delivering quality nutrition services in primary healthcare: a descriptive analysis of patient experience and provider competence in 11 low-income and middle-income countries | Not included | Duplicate |
|  | Capacity and quality of maternal and child health services delivery at the subnational primary healthcare level in relation to intermediate health outputs: a cross-sectional study of 12 low-income and middle-income countries | Not included | Duplicate |
|  | Waste Management With Special emphasis on occupational health and safety at selected healthcare establishments | Not included | Duplicate |
|  | Factors affecting the serological testing of cadaveric donor cornea | Not included | Duplicate |
|  | Identifying occupational hazards among healthcare workers in Australia and Bhutan | Not included | Duplicate |
|  | Chapter 30 - Occupational Health of Laboratory Animal Workers | Not included | Duplicate |
|  | Prevalence of hepatitis B and C viral infections in Pakistan: findings of a national survey appealing for effective prevention and control measures | Not included | Duplicate |
|  | Immunizations and oral health care providers | Not included | Duplicate |
|  | A positive-feedback loop between HBx and ALKBH5 promotes hepatocellular carcinogenesis | Not included | Duplicate |
|  | Serum Liver Fibrosis Markers in the Prognosis of Liver Cirrhosis: A Prospective Observational Study | Not included | Duplicate |
|  | Expansion of Stem Cell-Like CD4(+) Memory T Cells during Acute HIV-1 Infection Is Linked to Rapid Disease Progression | Not included | Duplicate |
|  | Cancer risk by social class and occupation: a survey of 109,000 cancer cases among Finns of working age | Not included | Duplicate |
|  | Improved Antibody Response to Three Additional Hepatitis B Vaccine Doses Following Primary Vaccination Failure in Patients with Inflammatory Bowel Disease | Not included | Duplicate |
|  | Sleep and antibody response to hepatitis B vaccination | Not included | Duplicate |
|  | Emerging and Re-emerging Pathogens and Diseases, and Health Consequences of a Changing Climate | Not included | Duplicate |
|  | Nurses and AIDS care: Occupational risk perception and the social construction of HIV | Not included | Duplicate |
|  | Liver steatosis in children with chronic hepatitis B and C: Prevalence, predictors, and impact on disease progression | Not included | Duplicate |
|  | Prevalence of hepatitis B virus (HBV) infection among Makerere University medical students | Not included | Duplicate |
|  | Suppl-1, M3: epidemiology of hepatitis B virus (HBV) and hepatitis C virus (HCV) related hepatocellular carcinoma | Not included | Duplicate |
|  | Hepatitis B virus infection and vaccine-induced immunity in Madrid (Spain) | Not included | Duplicate |
|  | Costs of needlestick injuries and subsequent hepatitis and HIV infection | Not included | Duplicate |
|  | To Study the Incidence, Predictive Factors and Clinical Outcome of Spontaneous Bacterial Peritonitis in Patients of Cirrhosis with Ascites | Not included | Duplicate |
|  | Delayed-type hypersensitivity and hepatitis B vaccine responses, in vivo markers of cellular and humoral immune function, and the risk of AIDS or death | Not included | Duplicate |
|  | Sharps injuries among hospital workers in Massachusetts, 2007 | Not included | Duplicate |
|  | Clinical features of HBsAg seroclearance in hepatitis B virus carriers in South Korea: A retrospective longitudinal study | Not included | Duplicate |
|  | Global patterns of hepatocellular carcinoma management from diagnosis to death: the BRIDGE Study | Not included | Duplicate |
|  | Assessing significant fibrosis using imaging-based elastography in chronic hepatitis B patients: Pilot study | Not included | Duplicate |
|  | Liver Transplantation from Brain-Dead Donors with Hepatitis B or C in South Korea: A 2014-2020 Korean Organ Transplantation Registry Data Analysis | Not included | Duplicate |
|  | Ten-Year Changes in the Hepatitis B Prevalence in the Birth Cohorts in Korea: Results From Nationally Representative Cross-Sectional Surveys | Not included | Duplicate |
|  | A Study to Assess the Knowledge and Attitude Regarding Occupational Exposure and Post Exposure Prophylaxis (PEP) for Hiv Among Student Nurses of Selected Nursing Institutes of Hubballi with a View to Develop an Information Guide Sheet | Not included | Duplicate |
|  | Observational and Genetic Associations of Body Mass Index and Hepatobiliary Diseases in a Relatively Lean Chinese Population | Not included | Duplicate |
|  | Prevalence, risk factors and virological profile of chronic hepatitis B virus infection in pregnant women in India | Not included | Duplicate |
|  | A novel system for predicting liver histopathology in patients with chronic hepatitis B | Not included | Duplicate |
|  | Hepatitis B immunization data of patients living with HIV/AIDS: a multi-centre study | Not included | Duplicate |
|  | Human immunodeficiency virus infection predictors and genetic diversity of hepatitis B virus and hepatitis C virus co-infections among drug users in three major Kenyan cities | Not included | Duplicate |
|  | Global epidemiology of hepatitis B virus infection: new estimates of age-specific HBsAg seroprevalence and endemicity | Not included | Duplicate |
|  | Exposure time to hepatitis B virus and associated risk factors among children in Edirne, Turkey | Not included | Duplicate |
|  | Hepatitis B virus infection among illegal drug users in Enugu State, Nigeria: prevalence, immune status, and related risk factors | Not included | Duplicate |
|  | Staff Nurse Education on Best Practices for Preventing Blood-Borne Pathogen Exposures | Not included | Duplicate |
|  | Evaluating vertical transmission of sexually transmitted infections to newborns | Not included | Duplicate |
|  | A systematic review and meta-analysis of the prevalence of hepatitis B virus infection among pregnant women in Nigeria | Not included | Duplicate |
|  | Knowledge, attitude, and risk factors of hepatitis B among waste scavengers in Lagos, Nigeria | Not included | Duplicate |
|  | A multi-centre cross-sectional study on hepatitis B vaccination coverage and associated factors among personnel working in health facilities in Kumasi, Ghana | Not included | Duplicate |
|  | The impact of HIV on hepatocellular cancer survival in Nigeria | Not included | Duplicate |
|  | Factors Influencing Hospital Cleaners’ Knowledge and Practices toward Hepatitis B prevention in Northern Province of Rwanda | Not included | Duplicate |
|  | A proposed predictive model for advanced fibrosis in patients with chronic hepatitis B and its validation | Not included | Duplicate |
|  | Hepatitis C prevalence and associated risk factors among individuals who are homeless and diagnosed with mental illness: At Home/Chez Soi Study, Vancouver, BC | Not included | Duplicate |
|  | Hepatitis A vaccines | Not included | Duplicate |
|  | Prevalence of hepatitis B virus infection in Nigeria, 2000-2013: A systematic review and meta-analysis | Not included | Duplicate |
|  | Seroprevalence of hepatitis B virus among antenatal clinic attendees in Gamawa Local Government Area, Bauchi State, Nigeria | Not included | Duplicate |
|  | Syphilis and HIV prevalence and associated factors to their co-infection, hepatitis B and hepatitis C viruses prevalence among female sex workers in Rwanda | Not included | Duplicate |
|  | Prevalence of Hepatitis B Virus (HBV) surface antigen and HBVassociated hepatocellular carcinoma in Kenyans of various ages | Not included | Duplicate |
|  | A nationwide cross-sectional review of in-hospital hepatitis B virus testing and disease burden estimation in Ghana, 2016 - 2021 | Not included | Duplicate |
|  | Studies on prevalence and risk factors for Hepatitis B Surface Antigen among secondary school students in north-central, Nigeria | Not included | Duplicate |
|  | Multidimensional Analysis of the Mother-to-child Transmission Risk Factors in Chronic Hepatitis B Virus Infection in Pregnant Women in Vietnam | Not included | Duplicate |
|  | Healthcare resource utilization and costs by disease severity in an insured national sample of US patients with chronic hepatitis B | Not included | Duplicate |
|  | Advancing Age and Comorbidity in a US Insured Population-Based Cohort of Patients With Chronic Hepatitis B | Not included | Duplicate |
|  | Hepatitis B‐related hepatocellular carcinoma: epidemiological characteristics and disease burden | Not included | Duplicate |
|  | An enormous hepatitis B virus‐related liver disease burden projected in Vietnam by 2025 | Not included | Duplicate |
|  | Hepatitis B in healthcare personnel: an update on the global landscape | Not included | Duplicate |
|  | Prevalence, infectivity and correlates of hepatitis B virus infection among pregnant women in a rural district of the Far North Region of Cameroon | Not included | Duplicate |
|  | Assessing risk behaviors and prevalence of sexually transmitted and blood-borne infections among female crack cocaine users in Salvador-Bahia, Brazil | Not included | Duplicate |
|  | Standard Precautions among HealthCare Workers in a Tertiary Health Facility in Enugu Metropolis, South-East Nigeria | Not included | Duplicate |
|  | HIV viraemia during hepatitis B vaccination shortens the duration of protective antibody levels | Not included | Duplicate |
|  | Viral load is a significant prognostic factor for hepatitis B virus‐associated hepatocellular carcinoma | Not included | Duplicate |
|  | Dual positivity of hepatitis B surface antigen and anti-hepatitis C virus antibody and associated factors among apparently healthy patients of Ekiti State, Nigeria | Not included | Duplicate |
|  | Hepatitis C virus infection and its associated factors among prisoners in a Nigerian prison | Not included | Duplicate |
|  | Early cranial ultrasound findings among infants with neonatal encephalopathy in Uganda: an observational study | Not included | Duplicate |
|  | Sero-prevalence of hepatitis B virus and associated factors among pregnant women in Gambella hospital, South Western Ethiopia: facility based cross-sectional study | Not included | Duplicate |
|  | Clinical characteristics and current management of hepatitis B and C in China | Not included | Duplicate |
|  | No contribution of lifestyle and environmental exposures to gender discrepancy of liver disease severity in chronic hepatitis b infection: Observations from the Haimen City cohort | Not included | Duplicate |
|  | Sero-Prevalence of Hepatitis B Virus Infection and Associated Factors Among Pregnant Women Attending Antenatal Care Services in Gedeo Zone, Southern Ethiopia | Not included | Duplicate |
|  | Effects of long-term antiretroviral therapy in reproductive-age women in sub-Saharan Africa (the PEPFAR PROMOTE study): a multi-country observational cohort study | Not included | Duplicate |
|  | Prevalence, genotype distribution and mutations of hepatitis B virus and the associated risk factors among pregnant women residing in the northern shores of Persian Gulf, Iran | Not included | Duplicate |
|  | Global Estimates on Biological Risks at Work | Not included | Duplicate |
|  | Telbivudine treatment of hepatitis B virus-infected pregnant women at different gestational stages for the prevention of mother-to-child transmission: Outcomes of telbivudine treatment during pregnancy | Not included | Duplicate |
|  | Occult Hepatitis B Virus Infection in Maintenance Hemodialysis Patients: Prevalence and Mutations in "a" Determinant | Not included | Duplicate |
|  | Prevalence of hepatitis B virus infection in Shenzhen, China, 2015–2018 | Not included | Duplicate |
|  | Identifying, preventing and controlling needle-stick injuries in Indonesia | Not included | Duplicate |
|  | Splash of body fluids among healthcare support staff in Ghana: a cross-sectional study | Not included | Duplicate |
|  | Physiological and psychosocial stressors among hemodialysis patients in the Buea Regional Hospital, Cameroon | Not included | Duplicate |
|  | Safety practice and associated factors among waste handlers in Governmental Hospitals in Addis Ababa, Ethiopia | Not included | Duplicate |
|  | Hepatitis B virus infection and associated risk factors among medical students in eastern Ethiopia | Not included | Duplicate |
|  | Risk factors associated with Hepatitis B virus infection among pregnant women attending public hospitals in Addis Ababa, Ethiopia | Not included | Duplicate |
|  | Clinicopathological analysis of patients with dual malignancies: A retrospective study | Not included | Duplicate |
|  | Prevalence and the associated factors of hepatitis B and hepatitis C viral infections among HIV-positive individuals in same-day antiretroviral therapy initiation program in Bangkok, Thailand | Not included | Duplicate |
|  | Prevalence of liver steatosis in patients with chronic hepatitis B: a study of associated factors and of relationship with fibrosis | Not included | Duplicate |
|  | Hepatitis B, hepatitis C, and mortality among HIV-positive individuals | Not included | Duplicate |
|  | Nephrotoxicity caused by oral antiviral agents in patients with chronic hepatitis B treated in a hospital for tropical diseases in Thailand | Not included | Duplicate |
|  | Sexual behaviour and practices among adolescent blood donors in Harare and Masvingo provinces, Zimbabwe | Not included | Duplicate |
|  | Thermal disinfection in hemodialysis using the A0 concept as dispenser | Not included | Duplicate |
|  | Foodborne Diseases: Overview of Biological Hazards and Foodborne Diseases | Not included | Duplicate |
|  | Guidelines for Preventing Infectious Complications among Hematopoietic Cell Transplantation Recipients: A Global Perspective | Not included | Duplicate |
|  | Barriers to hepatitis B vaccine coverage among healthcare workers in the Republic of Georgia: An international perspective | Not included | Duplicate |
|  | The impact of economic and social factors on the prevalence of hepatitis B in Turkey | Not included | Duplicate |
|  | Proliferative lupus nephritis in the absence of overt systemic lupus erythematosus: A historical study of 12 adult patients | Not included | Duplicate |
|  | Seroprevalence of hepatitis B and C virus infections and risk factors in Turkey: a fieldwork TURHEP study | Not included | Duplicate |
|  | Chronic liver disease detection and quantification | Not included | Duplicate |
|  | Inequalities in the use of secondary prevention of cardiovascular disease by socioeconomic status: evidence from the PURE observational study | Not included | Duplicate |
|  | Prevention of hepatitis B and C in the EU/EEA April 2024 | Not included | Duplicate |
|  | Sero-Prevalence of HBV and its Associated Factors Among Healthcare Providers in Public Health Facilities in Eastern Ethiopia | Not included | Duplicate |
|  | An Assessment of the Knowledge, Attitude, and Practice Toward Standard Precautions Among Health Workers From a Hospital in Northern Cyprus | Not included | Duplicate |
|  | Burden of Hepatitis-B Infections and Risk Factors among Healthcare Workers in Resource Limited Setting, Addis Ababa, Ethiopia | Included | Duplicate |
|  | Knowledge, attitude and prevalence of hepatitis B virus among healthcare workers: a cross- sectional, Hospital based study in Bamenda Health District, NWR, Cameroon | Not included | Duplicate |
|  | Seroprevalence of Hepatitis B Surface Antigen and Occupational Risk Factors Among Health Care Workers in Ekiti State, Nigeria | Not included | Duplicate |
|  | Epidemiology of viral hepatitis and HIV co-infection | Not included | Duplicate |
|  | The exposure rate to hepatitis B and C viruses among medical waste handlers in three government hospitals, southern Ethiopia | Included | Duplicate |
|  | Assessment of Knowledge, Attitude and Vaccination Status of Hepatitis B among Nursing Training Students in Ho, Ghana | Not included | Duplicate |
|  | Precautions for health care workers to avoid hepatitis B and C virus infection | Not included | Duplicate |
|  | Serological Evidence and Associated Factors of Hepatitis B Virus and Hepatitis C Virus Among Waste Handlers: A Cross-Sectional Study from Northeastern Ethiopia | Included | Duplicate |
|  | Systematic review and meta-analysis of HIV, HBV and HCV infection prevalence in Sudan | Not included | Duplicate |
|  | Hepatitis B Virus Infection and Associated Factors Among Adults in Southwest Ethiopia: Community-Based Cross-Sectional Study | Not included | Duplicate |
|  | Prevalence of hepatitis B virus markers in surgeons in Lagos, Nigeria | Included | Duplicate |
|  | Risk and management of blood-borne infections in health care worker | Not included | Duplicate |
|  | Hepatitis B vaccination status and needle-stick and sharps-related Injuries among medical school students in Nepal: a cross-sectional study | Not included | Duplicate |
|  | Seroprevalence of hepatitis B virus infection in Cameroon: a systematic review and meta-analysis | Not included | Duplicate |
|  | A historical perspective on the discovery and elucidation of the hepatitis B virus | Not included | Duplicate |
|  | Hepatitis B infection among health workers in Uganda: evidence of the need for health worker protection | Included | Duplicate |
|  | Hepatitis B Virus Blood Screening: Need for Reappraisal of Blood Safety Measures? | Not included | Duplicate |
|  | Coverage of hepatitis B vaccination in Swedish healthcare workers | Not included | Duplicate |
|  | Prevalence of hepatitis B surface antigen (HbsAg) among health professionals in public Hospitals in Addis Ababa, Ethiopia | Not included | Duplicate |
|  | Hepatitis B and human immunodeficiency virus co-infection among pregnant women in resource-limited high endemic setting, Addis Ababa, Ethiopia: implications for prevention and control measures | Not included | Duplicate |
|  | Knowledge, Attitude, and Practice towards Hepatitis B Infection Prevention and Screening among Indonesians | Not included | Duplicate |
|  | Epidemiological patterns of hepatitis B virus (HBV) in highly endemic areas | Not included | Duplicate |
|  | Seroprevalence of Hepatitis B virus infection and associated factors among health care workers in Southern Ghana | Included | Duplicate |
|  | Economic burden of hepatitis C-associated diseases: Europe, Asia Pacific, and the Americas | Not included | Duplicate |
|  | Seroprevalence of hepatitis B surface antigenaemia among healthcare worker in a private Nigerian tertiary health institution | Included | Duplicate |
|  | Hepatitis B Virus Infection in Pregnant Women, in Al Fashir Town, North Darfur State, Sudan | Not included | Duplicate |
|  | Epidemiologic and socioeconomic factors impacting hepatitis B virus and related hepatocellular carcinoma | Not included | Duplicate |
|  | Prevalence and correlates of hepatitis B and C seropositivity among health care workers in a semi urban setting in North Central Nigeria | Not included | Duplicate |
|  | Epidemiology of viral hepatitis in Somalia: Systematic review and meta-analysis study | Not included | Duplicate |
|  | The global burden of disease attributable to contaminated injections given in health care settings | Not included | Duplicate |
|  | Hepatitis B and C Viral Infection: Prevalence, Knowledge, Attitude, Practice, and Occupational Exposure among Healthcare Workers of Jimma University Medical Center, Southwest Ethiopia | Not included | Duplicate |
|  | Prevalence and predictors of hepatitis B virus (HBV) infection in east Africa: evidence from a systematic review and meta-analysis of epidemiological studies published from 2005 to 2020 | Not included | Duplicate |
|  | Hepatitis B and C seroprevalence among health care workers in a tertiary hospital in Rwanda | Included | Duplicate |
|  | Prevalence of hepatitis B virus infection and uptake of hepatitis B vaccine among healthcare workers, Makueni County, Kenya 2017 | Included | Duplicate |
|  | High prevalence of hepatitis B infections in Burkina Faso (1996-2017): a systematic review with meta-analysis of epidemiological studies | Not included | Duplicate |
|  | Impact of the national hepatitis B immunization program in China: a modeling study | Not included | Duplicate |
|  | Changing prevalence of chronic hepatitis B virus infection in China between 1973 and 2021: a systematic literature review and meta-analysis of 3740 studies and 231 million people | Not included | Duplicate |
|  | Assessing the prevalence of hepatitis B virus infection among health care workers in a referral hospital in Kisantu, Congo DR: a pilot study | Included | Duplicate |
|  | Seroprevalence of Hepatitis B Among Healthcare Workers in Asia and Africa and Its Association With Their Knowledge and Awareness: A Systematic Review and Meta-Analysis | Not included | Duplicate |
|  | Prevalence of hepatitis B virus and immunity status among healthcare workers in Beira City, Mozambique | Included | Duplicate |
|  | Seroprevalence and Knowledge of Hepatitis B Virus Infection Among Laboratory Workers at Kilimanjaro Christian Medical Centre in Moshi, Tanzania | Included | Duplicate |
|  | Hepatitis B virus in Lao dentists: A cross-sectional serological study | Not included | Duplicate |
|  | Cross sectional study of chronic hepatitis B prevalence among healthcare workers in an urban setting, Sierra Leone | Included | Duplicate |
|  | Distribution of hepatitis B virus genotypes among patients with chronic infection in Japan shifting toward an increase of genotype A | Not included | Duplicate |
|  | Hepatitis B Virus and Human Immunodeficiency Virus Infections among Health Care Workers in Some Health Care Centers in Benue State, Niger | Included | Duplicate |
|  | Hepatitis B virus infection status and associated factors among health care workers in selected hospitals in Kisumu County, Kenya: A cross-sectional study | Included | Duplicate |
|  | Prevalence of HBV, HCV and Associated Risk Factors Among Cleaners at Selected Public Health Centers in Addis Ababa, Ethiopia | Included | Duplicate |
|  | Hepatitis B virus infection and its associated factors among medical waste collectors at public health facilities in eastern Ethiopia: a facility-based cross-sectional study | Included | Duplicate |
|  | Prevalence of hepatitis B virus and associated risk factors among adults patients at Dessie referral and Kemise general hospitals in northeastern Ethiopia | Not included | Duplicate |
|  | Seroprevalence of hepatitis B surface antigen and anti HCV antibody and its associated risk factors among pregnant women attending maternity ward of Felege Hiwot Referral Hospital, northwest Ethiopia: a cross-sectional study | Not included | Duplicate |
|  | Prevalence of hepatitis B virus infection among health care workers in a tertiary hospital in Tanzania | Not included | Duplicate |
|  | Prevalence of hepatitis B virus infection in Nigeria, 2000-2013: a systematic review and meta-analysis | Not included | Duplicate |
|  | Seroprevalence of Hepatitis B and C among health care workers in Omdurman, Sudan | Included | Duplicate |
|  | Hepatitis B Prevalence, Knowledge and Occupational Factors among Health Care Workers in Fako Division, South West Region Cameroon | Not included | Duplicate |
|  | An enormous hepatitis B virus-related liver disease burden projected in Vietnam by 2025 | Not included | Duplicate |
|  | Hepatitis B in Healthcare Personnel: An Update on the Global Landscape | Not included | Duplicate |
|  | Hepatitis B infection is highly prevalent among patients presenting with jaundice in Kenya | Not included | Duplicate |
|  | Hepatitis B in Ghana: a systematic review & meta-analysis of prevalence studies (1995-2015) | Not included | Duplicate |
|  | Hepatitis B Fact sheet | Not included | Duplicate |
|  | The prevalence of serological markers for hepatitis B virus infection in Australian Naval personnel | Not included | Duplicate |
|  | The prevalence of serological markers for hepatitis B virus infection in Australian Naval personnel | Not included | Duplicate |
|  | Is universal HBV vaccination of healthcare workers a relevant strategy in developing endemic countries? The case of a university hospital in Niger | Not included | Duplicate |
|  | Prevalence and associated knowledge of hepatitis B infection among healthcare workers in Freetown, Sierra Leone | Included | Duplicate |
|  | Enzyme-linked immunosorbent assay for the quantitative/qualitative analysis of plant secondary metabolites | Not included | Duplicate |
|  | Occupational injury history and universal precautions awareness: a survey in Kabul hospital staff | Not included | Duplicate |
|  | Seroprevalence of Hepatitis B and C among Healthcare Workers in Dutse Metropolis Jigawa State, Nigeria | Included | Duplicate |
|  | Estimations of worldwide prevalence of chronic hepatitis B virus infection: a systematic review of data published between 1965 and 2013 | Not included | Duplicate |
|  | Seroprevalence of hepatitis B virus infection and associated factors among healthcare workers in northern Tanzania | Included | Duplicate |
|  | Impact of Training about Knowledge, Attitude and Practice of Hepatitis B infection and its Vaccination among Healthcare Professionals in a Tertiary Care Hospital of Northern Gujarat | Not included | Duplicate |
|  | A new approach to prevent, diagnose, and treat hepatitis B in Africa | Not included | Duplicate |
|  | Effectiveness of Hepatitis B Vaccination Campaign in Italy: Towards the Control of HBV Infection for the First Time in a European Country | Not included | Duplicate |
|  | Prevalence of central obesity and associated factors in Ethiopia: A systematic review and meta-analysis | Not included | Duplicate |
|  | Global, regional, and national incidence, prevalence, and years lived with disability for 301 acute and chronic diseases and injuries in 188 countries, 1990-2013: a systematic analysis for the Global Burden of Disease Study 2013 | Not included | Duplicate |
|  | Hepatitis | Not included | Duplicate |
|  | Health care worker safety | Not included | Duplicate |
|  | Global health sector strategy on Viral hepatitis 2016–2021 | Not included | Duplicate |
|  | Global Hepatitis Report | Not included | Duplicate |
|  | Overcoming fragility in Somalia to build a strong primary health care system | Not included | Duplicate |
|  | Occupational health: health workers | Not included | Duplicate |
|  | Hepatitis B | Not included | Duplicate |
|  | Global hepatitis report: Action for access in low- and middle-income countries | Not included | Duplicate |
|  | Sero-Prevalence and Associated Factors of Hepatitis B Virus Infection among Health Professionals in Adama Town, Oromia, Central Ethiopia | Included | Duplicate |
|  | Sero-prevalence of hepatitis B virus infection and associated factors among health care workers and medical waste handlers in primary hospitals of North-west Ethiopia | Included | Duplicate |
|  | Seroprevalence and risk factors for hepatitis B infection in an adult population in Northeast China | Not included | Duplicate |
|  | Let's Talk About B: Barriers to Hepatitis B Screening and Vaccination Among Asian and South Asian Immigrants in British Columbia | Not included | Duplicate |
|  | Sero-prevalence and risk factors for hepatitis B virus infection among health care workers in a tertiary hospital in Uganda | Included | Duplicate |
|  | Gamma-glutamyl transpeptidase-to-platelet ratio and the fibrosis-4 index in predicting hepatitis B virus-related hepatocellular carcinoma development in elderly chronic hepatitis B patients in China: A single-center retrospective study | Not included | Duplicate |
|  | Epidemiology of hepatitis B virus infection: results from a community-based study of 0.15 million residents in South China | Not included | Duplicate |
|  | Sero-prevalence and risk factors of hepatitis B virus and human immunodeficiency virus infection among pregnant women in Bahir Dar city, Northwest Ethiopia: a cross sectional study | Not included | Duplicate |
|  | Out of hospital cardiac arrest: when to resuscitat | Not included | Duplicate |
|  | Sharps injuries among health care workers in Cairo University Hospitals | Not included | Duplicate |
|  | Impact of Occupational Hazards on Healthcare Professionals' Mental (Psychological) Health: Evidence from Government-Owned Hospitals in Khulna, Bangladesh | Not included | Duplicate |
|  | Health Profiles of Newly Arrived Refugee Children in the United States, 2006-2012 | Not included | Duplicate |
|  | Cardiac health in patients with hepatitis B virus-related cirrhosis | Not included | Duplicate |
|  | Prevalence and Associated Factors of Human Papillomavirus Infection among Iraqi Women | Not included | Duplicate |
|  | Hepatitis C Virus Cascades of Care in the era of Direct-Acting Antiviral Therapy | Not included | Duplicate |
|  | Maternal health in China - challenges of the next decade | Not included | Duplicate |
|  | A study on prevention of bleeding complications using lusutrombopag for safe RFA in patients with hepatocellular carcinoma with low platelet counts: prospective observational study | Not included | Duplicate |
|  | Validation of the Korean Stroop Test in Diagnosis of Minimal Hepatic Encephalopathy | Not included | Duplicate |
|  | Seroprevalence and predictors of hepatitis B virus infection among pregnant women attending routine antenatal care in Arba Minch Hospital, South Ethiopia | Not included | Duplicate |
|  | Conservation and variability of hepatitis B core at different chronic hepatitis stages | Not included | Duplicate |
|  | Prevalence of Hepatitis B virus infection and its determinants among pregnant women in East Africa: Systematic review and Meta-analysis | Not included | Duplicate |
|  | HCC risk reduction with oral nucleos(t)ide analogues in patients with chronic hepatitis B: Not perfect, not good enough | Not included | Duplicate |
|  | Occupational exposures to blood and body fluids (BBFS) among health care workers and medical students in University of Gondar Hospital, Northwest of Ethiopia | Not included | Duplicate |
|  | Somatosensory Amplification, Anxiety, and Depression in Patients With Hepatitis B: Impact on Functionality | Not included | Duplicate |
|  | Health behaviors of Korean adults with hepatitis B: Findings of the 2016 Korean National Health and Nutrition Examination Survey | Not included | Duplicate |
|  | Prevalence of and risk factors for hepatitis C virus antibody among people who inject drugs in Cambodia: a national biological and behavioral survey | Not included | Duplicate |
|  | An updated systematic review and meta-analysis of the prevalence of hepatitis B virus in Ethiopia | Not included | Duplicate |
|  | Management of Takotsubo cardiomyopathy in non-academic hospitals in France: The Observational French SyndromEs of TakoTsubo (OFSETT) study | Not included | Duplicate |
|  | Clinical characteristics, prognosis, and surgical outcomes of patients with non-HBV and non-HCV related hepatocellular carcinoma: three-decade observational study | Not included | Duplicate |
|  | Characteristics of female sexual dysfunctions and obstetric complications related to female genital mutilation in Omdurman maternity hospital, Sudan | Not included | Duplicate |
|  | Prognostic value of immunoscore to identify mortality outcomes in adults with HBV-related primary hepatocellular carcinoma | Not included | Duplicate |
|  | The association of adverse outcomes in the mother with disease progression in offspring in families with clusters of hepatitis B virus infection and unfavorable prognoses in Northwest China | Not included | Duplicate |
|  | Hospital acquired infections and infection prevention practices in teaching hospitals in the Amhara regional state, Ethiopia | Not included | Duplicate |
|  | Sero-prevalence of hepatitis B virus infection and its risk factors among pregnant women attending antenatal clinic at Aminu Kano Teaching Hospital, Kano, Nigeria | Not included | Duplicate |
|  | Relationship of Treg/Th17 balance with HBeAg change in HBeAg-positive chronic hepatitis B patients receiving telbivudine antiviral treatment: A longitudinal observational study | Not included | Duplicate |
|  | Occupational exposures to blood and body fluids (BBFS) among health care workers and medical students in University of Gondar Hospital, Northwest of Ethiopia | Not included | Duplicate |
|  | Telbivudine and adefovir dipivoxil combination therapy improves renal function in patients with chronic hepatitis B: A STROBE-compliant article | Not included | Duplicate |
|  | Outcomes of Glucocorticoid Treatment in HBV Associated Acute-on-Chronic Liver Failure Patients: A Retrospective Observational Study | Not included | Duplicate |
|  | Multimodal Ultrasound Model Based on the Left Gastric Vein in B-Viral Cirrhosis: Noninvasive Prediction of Esophageal Varices | Not included | Duplicate |
|  | Multiple Primary Malignancies in Patients With Hepatocellular Carcinoma: A Largest Series With 26-Year Follow-Up | Not included | Duplicate |
|  | Expression profiles of transcription factors for special CD4+ T-cell subsets in peripheral blood mononuclear cells from patients with hepatitis B virus infection | Not included | Duplicate |
|  | Hepatitis B virus-associated hepatocellular carcinoma | Not included | Duplicate |
|  | HBV pgRNA profiles in Chinese HIV/HBV coinfected patients under pre- and posttreatment: a multicentre observational cohort study | Not included | Duplicate |
|  | Population-Based Multicentric Survey of Hepatitis B Infection and Risk Factors in the North, South, and Southeast Regions of Brazil, 10-20 Years After the Beginning of Vaccination | Not included | Duplicate |
|  | Analysis of serum hepatitis B virus RNA levels among HBsAg and HBsAb copositive patients and its correlation with HBV DNA | Not included | Duplicate |
|  | Global Burden and Trends of Primary Liver Cancer Attributable to Comorbid Type 2 Diabetes Mellitus Among People Living with Hepatitis B: An Observational Trend Study from 1990 to 2019 | Not included | Duplicate |
|  | Systemic immune-inflammation index predicts postoperative acute kidney injury in hepatocellular carcinoma patients after hepatectomy | Not included | Duplicate |
|  | The imported infections among foreign travelers in China: an observational study | Not included | Duplicate |
|  | An updated systematic review and meta-analysis of the prevalence of hepatitis B virus in Ethiopia | Not included | Duplicate |
|  | Hospital acquired infections and infection prevention practices in teaching hospitals in the Amhara regional state, Ethiopia | Not included | Duplicate |
|  | 2022 International Consensus on Cardiopulmonary Resuscitation and Emergency Cardiovascular Care Science With Treatment Recommendations: Summary From the Basic Life Support; Advanced Life Support; Pediatric Life Support; Neonatal Life Support; Education, Implementation, and Teams; and First Aid Task Forces | Not included | Duplicate |
|  | Mercury Exposure and Poor Nutritional Status Reduce Response to Six Expanded Program on Immunization Vaccines in Children: An Observational Cohort Study of Communities Affected by Gold Mining in the Peruvian Amazon | Not included | Duplicate |
|  | Maternal exposure to carbon monoxide and fine particulate matter during pregnancy in an urban Tanzanian cohort | Not included | Duplicate |
|  | Sero-prevalence of hepatitis B virus infection and its risk factors among pregnant women attending antenatal clinic at Aminu Kano Teaching Hospital, Kano, Nigeria | Not included | Duplicate |
|  | Hepatitis B virus-associated hepatocellular carcinoma | Not included | Duplicate |
|  | Add-on pegylated interferon augments hepatitis B surface antigen clearance vs continuous nucleos(t)ide analog monotherapy in Chinese patients with chronic hepatitis B and hepatitis B surface antigen ≤ 1500 IU/mL: An observational study | Not included | Duplicate |
|  | Pneumococcal and influenza vaccination coverage among at-risk adults: A 5-year French national observational study | Not included | Duplicate |
|  | A modified MELD model for Chinese pre-ACLF and ACLF patients and it reveals poor prognosis in pre-ACLF patients | Not included | Duplicate |
|  | Analysis of serum hepatitis B virus RNA levels among HBsAg and HBsAb copositive patients and its correlation with HBV DNA | Not included | Duplicate |
|  | Statins improve outcomes of nonsurgical curative treatments in hepatocellular carcinoma patients | Not included | Duplicate |
|  | Genome-wide study of salivary microRNAs as potential noninvasive biomarkers for detection of nasopharyngeal carcinoma | Not included | Duplicate |
|  | Comparison of the efficacy and safety of entecavir and tenofovir in nucleos(t)ide analogue-naive chronic hepatitis B patients with high viraemia: a retrospective cohort study | Not included | Duplicate |
|  | Multicenter study of skin rashes and hepatotoxicity in antiretroviral-naïve HIV-positive patients receiving non-nucleoside reverse-transcriptase inhibitor plus nucleoside reverse-transcriptase inhibitors in Taiwan | Not included | Duplicate |
|  | Methylation status of the stimulator of interferon genes promoter in patients with chronic hepatitis B | Not included | Duplicate |
|  | Postnatal infection surveillance by telephone in Dar es Salaam, Tanzania: An observational cohort study | Not included | Duplicate |
|  | MRI findings in people with epilepsy and nodding syndrome in an area endemic for onchocerciasis: an observational study | Not included | Duplicate |
|  | Bacterial pathogenesis: a molecular approach | Not included | Duplicate |
|  | Clinical features of treatment-naive patients with hepatitis B virus infection: A community-based survey from high- and intermediate-hepatitis B endemicity regions in Southeast China | Not included | Duplicate |
|  | Rituximab plus chemotherapy as first-line treatment in Chinese patients with diffuse large B-cell lymphoma in routine practice: a prospective, multicentre, non-interventional study | Not included | Duplicate |
|  | Albumin-bilirubin and platelet-albumin-bilirubin grades for hepatitis B-associated hepatocellular carcinoma in Child-Pugh A patients treated with radical surgery: A retrospective observational study | Not included | Duplicate |
|  | Hepatocellular carcinoma amongst aboriginal and torres strait islander peoples of Australia | Not included | Duplicate |
|  | Hepatitis B vaccination status and associated factors among undergraduate students of Makerere University College of Health Sciences | Not included | Duplicate |
|  | Risk factors for underlying comorbidities and complications in patients with hepatitis B virus-related acute-on-chronic liver failure | Not included | Duplicate |
|  | Psychological profiles of excluded living liver donor candidates: An observational study | Not included | Duplicate |
|  | Prevalence of Hepatitis E Virus and Its Associated Outcomes among Pregnant Women in China | Not included | Duplicate |
|  | Clinical cure induced by pegylated interferon α-2b in the advantaged population of chronic hepatitis B virus infection: a retrospective cohort study | Not included | Duplicate |
|  | Prevalence and associated risk factors of Hepatitis B and Hepatitis C virus among volunteer blood donors in Arba Minch Blood Bank SNNPR, Ethiopia | Not included | Duplicate |
|  | Genetic variation in FCER1A predicts peginterferon alfa-2a-induced hepatitis B surface antigen clearance in East Asian patients with chronic hepatitis B | Not included | Duplicate |
|  | Cirrhosis and liver transplantation in patients co-infected with HIV and hepatitis B or C: an observational cohort study | Not included | Duplicate |
|  | Mutations in pre-core and basic core promoter regions of hepatitis B virus in chronic hepatitis B patients | Not included | Duplicate |
|  | Immune response pattern varies with the natural history of chronic hepatitis B | Not included | Duplicate |
|  | Role of interleukin-21 and interleukin-21 receptor polymorphisms in the treatment of HBeAg-positive chronic hepatitis B patients with peginterferon | Not included | Duplicate |
|  | The prevalence of hepatitis B virus infection in the United States in the era of vaccination | Not included | Duplicate |
|  | A Pilot Study of MicroRNAs Expression Profile in Serum and HBsAg Particles: Predictors of Therapeutic Vaccine Efficacy in Chronic Hepatitis B Patients | Not included | Duplicate |
|  | An epidemiological survey of HBV infection and low-level HBsAg in military camps in eastern China | Not included | Duplicate |
|  | Epidemiology and etiology of diffuse large B-cell lymphoma | Not included | Duplicate |
|  | Hepatitis B virus infection and related factors in hemodialysis patients in China–systematic review and meta-analysis | Not included | Duplicate |
|  | Prevalence of chronic obstructive pulmonary disease and associated risk factors in Uganda (FRESH AIR Uganda): a prospective cross-sectional observational study | Not included | Duplicate |
|  | Stopping nucleos(t)ide analogue treatment in Caucasian hepatitis B patients after HBeAg seroconversion is associated with high relapse rates and fatal outcomes | Not included | Duplicate |
|  | Caucasian Ethnicity, but Not Treatment Cessation is Associated with HBsAg Loss Following Nucleos(t)ide Analogue-Induced HBeAg Seroconversion | Not included | Duplicate |
|  | Prevalence an d factors associated with hepatitis B susceptibility among men who sex with men on HIV pre-exposure prophylaxis in Northeastern Brazil: a cross-sectional study | Not included | Duplicate |
|  | The Swiss STAR trial - an evaluation of target groups for sexually transmitted infection screening in the sub-sample of women | Not included | Duplicate |
|  | Poor adherence and low persistency rates for hepatocellular carcinoma surveillance in patients with chronic hepatitis B | Not included | Duplicate |
|  | Predictors of Hepatitis B Surface Antigen Titers two decades after vaccination in a cohort of students and post-graduates of the Medical School at the University of Palermo, Italy | Not included | Duplicate |
|  | High prevalence of hepatitis B virus and hepatitis D virus in the western Brazilian Amazon | Not included | Duplicate |
|  | Hepatitis B in Rondônia (Western amazon region, Brazil): descriptive analysis and spatial distribution | Not included | Duplicate |
|  | “I am still suffering:” The dilemma of multiple recoveries in the lives of methadone maintenance patients | Not included | Duplicate |
|  | Syphilis, human immunodeficiency virus, herpes genital and hepatitis B in a women's prison in Cochabamba, Bolivia: prevalence and risk factors | Not included | Duplicate |
|  | Switch from intravenous or intramuscular to subcutaneous hepatitis B immunoglobulin: effect on quality of life after liver transplantation | Not included | Duplicate |
|  | The value of APGA score, fibrosis index for diagnosing liver fibrosis in patients with chronic hepatitis B | Not included | Duplicate |
|  | Epidemiological patterns and risk factors associated with hepatitis B virus in Pakistani population | Not included | Duplicate |
|  | Protocol: Prospective observational study investigating the prevalence and clinical outcome of portopulmonary hypertension in Japanese patients with chronic liver disease | Not included | Duplicate |
|  | Chapter 26 - Hepatitis A Vaccines | Not included | Duplicate |
|  | Workplace hazards faced by nursing assistants in the United States: A focused literature review | Not included | Duplicate |
|  | Genotype Matters in Patients with Acute-on-chronic Liver Failure Due to Reactivation of Chronic Hepatitis B | Not included | Duplicate |
|  | Sex difference in the associations among risk factors with hepatitis B and C infections in a large Taiwanese population study | Not included | Duplicate |
|  | Protective effect of an improved immunization practice of mother-to-infant transmission of hepatitis B virus and risk factors associated with immunoprophylaxis failure | Not included | Duplicate |
|  | Prevalence and factors associated with hepatitis B immunization and infection among men who have sex with men in Beijing, China | Not included | Duplicate |
|  | Comparison of hepatitis B virus and hepatitis C virus prevalence and risk factors in a community-based study | Not included | Duplicate |
|  | Hepatitis C Virus Infection Associated With an Increased Risk of Deep Vein Thrombosis: A Population-Based Cohort Study | Not included | Duplicate |
|  | Low immediate postoperative platelet count is associated with hepatic insufficiency after hepatectomy | Not included | Duplicate |
|  | Clinical characteristics and risk factors of COVID-19 patients with chronic hepatitis B: a multi-center retrospective cohort study | Not included | Duplicate |
|  | A statistical analysis of the correlations among various types of clinical indexes for patients with chronic hepatitis B: A hospital-based study | Not included | Duplicate |
|  | Combined use of murine double minute-2 promoter methylation and serum AFP improves diagnostic efficiency in hepatitis B virus-related hepatocellular carcinoma | Not included | Duplicate |
|  | Abnormal IL-10 levels were related to alanine aminotransferase abnormalities during postpartum in HBeAg positive women with chronic hepatitis B | Not included | Duplicate |
|  | Gamma-glutamyl transpeptidase to platelet ratio index is a good noninvasive biomarker for predicting liver fibrosis in Chinese chronic hepatitis B patients | Not included | Duplicate |
|  | Risk factors for some tropical diseases in an African country | Not included | Duplicate |
|  | Epidemiology of hepatitis B virus infection in Bangladesh: prevalence among general population, risk groups and genotype distribution | Not included | Duplicate |
|  | Nosocomial infections: current situation in a resuscitation-unit | Not included | Duplicate |
|  | Hepatitis B virus infection | Not included | Duplicate |
|  | Does Nucleos(t)ide Analogues Treatment Affect Renal Function in Chronic Hepatitis B Patients Who Have Already Decreased eGFR? A Longitudinal Study | Not included | Duplicate |
|  | Seroepidemiology of the human herpesvirus 8 infection among people living with HIV in Taiwan, 2014-2018 | Not included | Duplicate |
|  | Sero-prevalence and risk factors for hepatitis B virus infection among the consumers of the alcoholic beverage, cheka in Konso zone, southwestern Ethiopia | Not included | Duplicate |
|  | Predictive factors for percutaneous and mucocutaneous exposure among healthcare workers in a developing country | Not included | Duplicate |
|  | Prevalence and predictors of hepatitis B virus coinfection in a United States cohort of hepatitis C virus‐infected patients | Not included | Duplicate |
|  | Superiority of tenofovir alafenamide fumarate over entecavir for serum HBsAg level reduction in patients with chronic HBV infection: A 144-week outcome study after switching of the nucleos(t)ide analog | Not included | Duplicate |
|  | The prevalence of hepatitis B and C viral infections among pregnant women | Not included | Duplicate |
|  | Prevalence and risk factors of hepatitis B virus transmission among children in Enugu, Nigeria | Not included | Duplicate |
|  | Hepatitis B virus infections and associated factors among pregnant women attending antenatal care clinic at Deder Hospital, Eastern Ethiopia | Not included | Duplicate |
|  | Cross-sectional study of chronic hepatitis B virus infection in Rwandan high-risk groups: unexpected findings on prevalence and its determinants | Not included | Duplicate |
|  | Screening a nation for hepatitis C virus elimination: a cross-sectional study on prevalence of hepatitis C and associated risk factors in the Rwandan general population | Not included | Duplicate |
|  | Role of quantitative hepatitis B surface antigen in predicting inactive carriers and HBsAg seroclearance in HBeAg-negative chronic hepatitis B patients | Not included | Duplicate |
|  | The role of Bcl-2 in hepatocarcinogenesis: Effects of overexpression on murine liver tumor development and hepatocyte cell cycle progression | Not included | Duplicate |
|  | CHAPTER 12 - Alcohol and Substance Abuse | Not included | Duplicate |
|  | Five‐year conditional survival for patients with hepatocellular carcinoma in Queensland, Australia | Not included | Duplicate |
|  | Admitted AIDS-associated Kaposi sarcoma patients: Indications for admission and predictors of mortality | Not included | Duplicate |
|  | Sustained viral response and relapse after discontinuation of oral antiviral drugs in HBeAg-positive patients with chronic hepatitis B infection | Not included | Duplicate |
|  | An observational study to evaluate the safety and efficacy of telbivudine in adults with chronic hepatitis B | Not included | Duplicate |
|  | Clinical outcome indicators in chronic hepatitis B and C: A primer for value-based medicine in hepatology | Not included | Duplicate |
|  | Patterns and co-occurrence of risk factors for hepatocellular carcinoma in four Asian American communities: a cross-sectional study | Not included | Duplicate |
|  | A Prospective Study Evaluating Changes in Histology, Clinical and Virologic Outcomes in HBV-HIV Co-infected Adults in North America | Not included | Duplicate |
|  | Long-term persistency of hepatitis B immunity: an observational cross-sectional study on medical students and resident doctors | Not included | Duplicate |
|  | Hepatitis B virus infection and factors associated with its acquisition among adults in a Lake Victoria HIV hyperendemic fishing community in Kyotera district, Uganda: a cross-sectional observation | Not included | Duplicate |
|  | Hepatitis B and C virus infection among 1.2 million persons with access to care: factors associated with testing and infection prevalence | Not included | Duplicate |
|  | Blood and virus detection on barber clippers | Not included | Duplicate |
|  | A new approach to prevent, diagnose, and treat hepatitis B in Africa | Not included | Duplicate |
|  | Infectious diseases prevalence, vaccination coverage, and diagnostic challenges in a population of internationally adopted children referred to a Tertiary Care Children's Hospital from 2009 to 2015 | Not included | Duplicate |
|  | Renal angina index in critically ill children as an applicable and reliable tool in the prediction of severe acute kidney injury: Two tertiary centers' prospective observational study from the Middle East | Not included | Duplicate |
|  | Associations Between Hepatitis B Virus Infection and Risk of All Cancer Types | Not included | Duplicate |
|  | Off-treatment virologic relapse and outcomes of re-treatment in chronic hepatitis B patients who achieved complete viral suppression with oral nucleos(t)ide analogs | Not included | Duplicate |
|  | Factors associated with sexually transmitted infections in sugarcane cutters: subsidies to caring for | Not included | Duplicate |
|  | Factors associated with immunoprophylaxis failure against vertical transmission of hepatitis B virus | Not included | Duplicate |
|  | Hospital personnel sero-protected against hepatitis B virus following an accelerated vaccination program | Not included | Duplicate |
|  | Predictors of liver disease progression in people living with HIV-HBV co-infection on antiretroviral therapy | Not included | Duplicate |
|  | High prevalence of hepatitis B-antibody loss and a case report of de novo hepatitis B virus infection in a child after living-donor liver transplantation | Not included | Duplicate |
|  | Sexually transmitted infections among patients attending a sexual assault centre: a cohort study from Oslo, Norway | Not included | Duplicate |
|  | Occupational risk perception in home health care workers | Not included | Duplicate |
|  | Post-vaccination anti-HBs testing among healthcare workers: More economical than post-exposure management for Hepatitis B | Not included | Duplicate |
|  | Blood donors and the supply of blood and blood products | Not included | Duplicate |
|  | Pro-Inflammatory Interleukin-18 is Associated with Hepatic Steatosis and Elevated Liver Enzymes in People with HIV Monoinfection | Not included | Duplicate |
|  | Sharps Injuries in Ambulatory Care: A Clinical Staff Perspective | Not included | Duplicate |
|  | Seroevidence for a high prevalence of subclinical infection with avian influenza A (H5N1) virus among workers in a live-poultry market in Indonesia | Not included | Duplicate |
|  | Effective therapeutic options for elderly patients with hepatocellular carcinoma: A nationwide cohort stud | Not included | Duplicate |
|  | Risk Factors for Renal Functional Decline in Chronic Hepatitis B Patients Receiving Oral Antiviral Agents | Not included | Duplicate |
|  | Prevalence and risk factors of hepatitis B virus, hepatitis C virus, and human immunodeficiency virus infections among drug addicts in Bangladesh | Not included | Duplicate |
|  | Seroprevalence of hepatitis B virus infection and associated factors among prison inmates in state of Mato Grosso do Sul, Brazil | Not included | Duplicate |
|  | Residual risk of mother-to-child transmission of hepatitis B virus infection despite timely birth-dose vaccination in Cameroon (ANRS 12303): a single-centre, longitudinal observational study | Not included | Duplicate |
|  | Dually Active HIV/HBV Antiretrovirals as Protection Against Incident Hepatitis B Infections: Potential for Prophylaxis | Not included | Duplicate |
|  | Practice and outcomes of neonatal resuscitation for newborns with birth asphyxia at Kakamega County General Hospital, Kenya: a direct observation study | Not included | Duplicate |
|  | Hepatitis B virus infection among medical waste handlers in Addis Ababa, Ethiopia | Not included | Duplicate |
|  | Information seeking behavior on hepatitis B virus, and its associated factors among pregnant women at teaching and specialized hospitals, Northwest Ethiopia: A cross-sectional study | Not included | Duplicate |
|  | Prevalence and risk factors of hepatic steatosis and its impact on liver injury in Chinese patients with chronic hepatitis B infection | Not included | Duplicate |
|  | Compliance and noncompliance of Universal Precautions among different groups of health care workers using the construct of the Health Belief Model: Implications for curriculum decision-making | Not included | Duplicate |
|  | Hepatitis B virus serosurvey and awareness of mother-to-child transmission among pregnant women in Shenyang, China: An observational study | Not included | Duplicate |
|  | The hepatitis B core antibody positive/hepatitis B surface antigen negative pattern is associated with the increased risk of intracranial atherosclerotic stenosis | Not included | Duplicate |
|  | Prevalence and factors associated with adverse drug reactions among heart failure patients hospitalized at Mbarara Regional Referral Hospital, Uganda | Not included | Duplicate |
|  | High seroprevalence and associated risk factors for hepatitis B virus infection among pregnant women living with HIV in Mtwara region, Tanzania | Not included | Duplicate |
|  | Acceptability and adherence to Isoniazid preventive therapy in HIV-infected patients clinically screened for latent tuberculosis in Dar es Salaam, Tanzania | Not included | Duplicate |
|  | Seroprevalence of hepatitis B virus infection and associated factors among healthcare workers in northern Tanzania | Not included | Duplicate |
|  | Seroprevalence of hepatitis B virus infection and associated factors among healthcare workers in northern Tanzania | Included | Duplicate |
|  | Multi-Omic Data Integration Allows Baseline Immune Signatures to Predict Hepatitis B Vaccine Response in a Small Cohort | Not included | Duplicate |
|  | Alcohol-Related Liver Disease Is Rarely Detected at Early Stages Compared With Liver Diseases of Other Etiologies Worldwide | Not included | Duplicate |
|  | Occupational safety and health in Spain | Not included | Duplicate |
|  | Infectious Diseases in Sub-Saharan Immigrants to Spain | Not included | Duplicate |
|  | Evaluating the appropriateness of chemotherapy in a low-resource cancer center in sub-Saharan Africa | Not included | Duplicate |
|  | Presentation, patterns of care, and outcomes of patients with prostate cancer in sub-Saharan Africa: A population-based registry study | Not included | Duplicate |
|  | Feasibility of the modified sequential organ function assessment score in a resource-constrained setting: a prospective observational study | Not included | Duplicate |
|  | Prevalence and risk factors associated with HIV/hepatitis B and HIV/hepatitis C co-infections among people who inject drugs in Mozambique | Not included | Duplicate |
|  | A prospective longitudinal study of psychosocial variables associated with the incidence of cancer among Seventh-day Adventists | Not included | Duplicate |
|  | Anti-TB drug concentrations and drug-associated toxicities among TB/HIV-coinfected patients | Not included | Duplicate |
|  | Occupational hazards | Not included | Duplicate |
|  | Estimations of worldwide prevalence of chronic hepatitis B virus infection: a systematic review of data published between 1965 and 2013 | Not included | Duplicate |
|  | Long-term follow-up of study participants from prophylactic HIV vaccine clinical trials in Africa | Not included | Duplicate |
|  | The Swiss STAR trial - an evaluation of target groups for sexually transmitted infection screening in the sub-sample of men | Not included | Duplicate |
|  | Using health-system-wide data to understand hepatitis B virus prophylaxis and reactivation outcomes in patients receiving rituximab | Not included | Duplicate |
|  | Poor clinical and virological outcome of nucleos(t)ide analogue monotherapy in HBV/HDV co-infected patients | Not included | Duplicate |
|  | A Study to Assess the Effectiveness of Structured Teaching Programme on Knowledge Regarding Universal Precautions and the Prevention of Blood Borne Infections Among the Final Year B. Sc. Nursing Students of Selected Nursing Colleges at Hassan, Karnataka | Not included | Duplicate |
|  | Impact of changing societal trends on the spread of infections in American and Canadian homes | Not included | Duplicate |
|  | The seroprevalence of the hepatitis B virus in Italian medical students after 3 decades since the introduction of universal vaccination | Not included | Duplicate |
|  | Correlates of infection and molecular characterization of blood-borne HIV, HCV, and HBV infections in HIV-1 infected inmates in Italy: An observational cross-sectional study | Not included | Duplicate |
|  | Prevalence and risk factors associated with hepatitis B and C in Nawabshah, Sindh, Pakistan | Not included | Duplicate |
|  | Percutaneous exposures among health care workers in a Greek tertiary hospital | Not included | Duplicate |
|  | Impact of Insulin Resistance on Therapeutic Response to Oral Treatment of Chronic Hepatitis C Virus Infection | Not included | Duplicate |
|  | Antiretroviral prophylaxis of health care workers at two urban medical centers | Not included | Duplicate |
|  | Patterns of antibiotic use, pathogens, and prediction of mortality in hospitalized neonates and young infants with sepsis: A global neonatal sepsis observational cohort study (NeoOBS) | Not included | Duplicate |
|  | Guidelines for the prevention of invasive mould diseases caused by filamentous fungi by the Spanish Society of Infectious Diseases and Clinical Microbiology (SEIMC) | Not included | Duplicate |
|  | Heterogeneity in neurocognitive change trajectories among people with HIV starting antiretroviral therapy in Rakai, Uganda | Not included | Duplicate |
|  | Early reduced liver graft survival in hepatitis C recipients identified by two combined genetic markers | Not included | Duplicate |
|  | Early reduced liver graft survival in hepatitis C recipients identified by two combined genetic markers | Not included | Duplicate |
|  | Seroprevalence of Hepatitis B Virus and Associated Factors Among Pregnant Women Attending Antenatal Care in Public Health Facilities in Jigjiga Town, Eastern Ethiopia | Not included | Duplicate |
|  | Career risk of hepatitis C virus infection among US emergency medical and public safety workers | Not included | Duplicate |
|  | Prevalence, risk factors, and outcomes for occult hepatitis B virus infection among HIV-infected patients | Not included | Duplicate |
|  | Reactive Blood Donor Notification; Their Responses And Perceptions: Experience From Southern Pakistan | Not included | Duplicate |
|  | Steatosis in chronic hepatitis B: prevalence and correlation with biochemical, histologic, viral, and metabolic parameters | Not included | Duplicate |
|  | Prevalence of hepatitis B virus, hepatitis C virus, and HIV infection among patients with newly diagnosed cancer from academic and community oncology practices | Not included | Duplicate |
|  | Nationwide retrospective study of hepatitis B virological response and liver stiffness improvement in 465 patients on nucleos(t)ide analogue | Not included | Duplicate |
|  | Hepatitis C virus infection in the Middle East and North Africa “MENA” region: injecting drug users (IDUs) is an under-investigated population | Not included | Duplicate |
|  | Novel point-of-care cytokine biomarker lateral flow test for the screening for sexually transmitted infections and bacterial vaginosis: study protocol of a multicentre multidisciplinary prospective observational clinical study to evaluate the performance and feasibility of the Genital InFlammation Test (GIFT) | Not included | Duplicate |
|  | Existing gaps and missed opportunities in delivering quality nutrition services in primary healthcare: a descriptive analysis of patient experience and provider competence in 11 low-income and middle-income countries | Not included | Duplicate |
|  | Capacity and quality of maternal and child health services delivery at the subnational primary healthcare level in relation to intermediate health outputs: a cross-sectional study of 12 low-income and middle-income countries | Not included | Duplicate |
|  | Waste Management With Special emphasis on occupational health and safety at selected healthcare establishments | Not included | Duplicate |
|  | Factors affecting the serological testing of cadaveric donor cornea | Not included | Duplicate |
|  | Identifying occupational hazards among healthcare workers in Australia and Bhutan | Not included | Duplicate |
|  | Chapter 30 - Occupational Health of Laboratory Animal Workers | Not included | Duplicate |
|  | Prevalence of hepatitis B and C viral infections in Pakistan: findings of a national survey appealing for effective prevention and control measures | Not included | Duplicate |
|  | Immunizations and oral health care providers | Not included | Duplicate |
|  | A positive-feedback loop between HBx and ALKBH5 promotes hepatocellular carcinogenesis | Not included | Duplicate |
|  | Serum Liver Fibrosis Markers in the Prognosis of Liver Cirrhosis: A Prospective Observational Study | Not included | Duplicate |
|  | Expansion of Stem Cell-Like CD4(+) Memory T Cells during Acute HIV-1 Infection Is Linked to Rapid Disease Progression | Not included | Duplicate |
|  | Cancer risk by social class and occupation: a survey of 109,000 cancer cases among Finns of working age | Not included | Duplicate |
|  | Improved Antibody Response to Three Additional Hepatitis B Vaccine Doses Following Primary Vaccination Failure in Patients with Inflammatory Bowel Disease | Not included | Duplicate |
|  | Sleep and antibody response to hepatitis B vaccination | Not included | Duplicate |
|  | Emerging and Re-emerging Pathogens and Diseases, and Health Consequences of a Changing Climate | Not included | Duplicate |
|  | Nurses and AIDS care: Occupational risk perception and the social construction of HIV | Not included | Duplicate |
|  | Liver steatosis in children with chronic hepatitis B and C: Prevalence, predictors, and impact on disease progression | Not included | Duplicate |
|  | Prevalence of hepatitis B virus (HBV) infection among Makerere University medical students | Not included | Duplicate |
|  | Suppl-1, M3: epidemiology of hepatitis B virus (HBV) and hepatitis C virus (HCV) related hepatocellular carcinoma | Not included | Duplicate |
|  | Hepatitis B virus infection and vaccine-induced immunity in Madrid (Spain) | Not included | Duplicate |
|  | Costs of needlestick injuries and subsequent hepatitis and HIV infection | Not included | Duplicate |
|  | To Study the Incidence, Predictive Factors and Clinical Outcome of Spontaneous Bacterial Peritonitis in Patients of Cirrhosis with Ascites | Not included | Duplicate |
|  | Delayed-type hypersensitivity and hepatitis B vaccine responses, in vivo markers of cellular and humoral immune function, and the risk of AIDS or death | Not included | Duplicate |
|  | Sharps injuries among hospital workers in Massachusetts, 2007 | Not included | Duplicate |
|  | Clinical features of HBsAg seroclearance in hepatitis B virus carriers in South Korea: A retrospective longitudinal study | Not included | Duplicate |
|  | Global patterns of hepatocellular carcinoma management from diagnosis to death: the BRIDGE Study | Not included | Duplicate |
|  | Assessing significant fibrosis using imaging-based elastography in chronic hepatitis B patients: Pilot study | Not included | Duplicate |
|  | Liver Transplantation from Brain-Dead Donors with Hepatitis B or C in South Korea: A 2014-2020 Korean Organ Transplantation Registry Data Analysis | Not included | Duplicate |
|  | Ten-Year Changes in the Hepatitis B Prevalence in the Birth Cohorts in Korea: Results From Nationally Representative Cross-Sectional Surveys | Not included | Duplicate |
|  | A Study to Assess the Knowledge and Attitude Regarding Occupational Exposure and Post Exposure Prophylaxis (PEP) for Hiv Among Student Nurses of Selected Nursing Institutes of Hubballi with a View to Develop an Information Guide Sheet | Not included | Duplicate |
|  | Observational and Genetic Associations of Body Mass Index and Hepatobiliary Diseases in a Relatively Lean Chinese Population | Not included | Duplicate |
|  | Prevalence, risk factors and virological profile of chronic hepatitis B virus infection in pregnant women in India | Not included | Duplicate |
|  | A novel system for predicting liver histopathology in patients with chronic hepatitis B | Not included | Duplicate |
|  | Hepatitis B immunization data of patients living with HIV/AIDS: a multi-centre study | Not included | Duplicate |
|  | Human immunodeficiency virus infection predictors and genetic diversity of hepatitis B virus and hepatitis C virus co-infections among drug users in three major Kenyan cities | Not included | Duplicate |
|  | Global epidemiology of hepatitis B virus infection: new estimates of age-specific HBsAg seroprevalence and endemicity | Not included | Duplicate |
|  | Exposure time to hepatitis B virus and associated risk factors among children in Edirne, Turkey | Not included | Duplicate |
|  | Hepatitis B virus infection among illegal drug users in Enugu State, Nigeria: prevalence, immune status, and related risk factors | Not included | Duplicate |
|  | Staff Nurse Education on Best Practices for Preventing Blood-Borne Pathogen Exposures | Not included | Duplicate |
|  | Evaluating vertical transmission of sexually transmitted infections to newborns | Not included | Duplicate |
|  | A systematic review and meta-analysis of the prevalence of hepatitis B virus infection among pregnant women in Nigeria | Not included | Duplicate |
|  | Knowledge, attitude, and risk factors of hepatitis B among waste scavengers in Lagos, Nigeria | Not included | Duplicate |
|  | A multi-centre cross-sectional study on hepatitis B vaccination coverage and associated factors among personnel working in health facilities in Kumasi, Ghana | Not included | Duplicate |
|  | The impact of HIV on hepatocellular cancer survival in Nigeria | Not included | Duplicate |
|  | Factors Influencing Hospital Cleaners’ Knowledge and Practices toward Hepatitis B prevention in Northern Province of Rwanda | Not included | Duplicate |
|  | A proposed predictive model for advanced fibrosis in patients with chronic hepatitis B and its validation | Not included | Duplicate |
|  | Hepatitis C prevalence and associated risk factors among individuals who are homeless and diagnosed with mental illness: At Home/Chez Soi Study, Vancouver, BC | Not included | Duplicate |
|  | Hepatitis A vaccines | Not included | Duplicate |
|  | Prevalence of hepatitis B virus infection in Nigeria, 2000-2013: A systematic review and meta-analysis | Not included | Duplicate |
|  | Seroprevalence of hepatitis B virus among antenatal clinic attendees in Gamawa Local Government Area, Bauchi State, Nigeria | Not included | Duplicate |
|  | Syphilis and HIV prevalence and associated factors to their co-infection, hepatitis B and hepatitis C viruses prevalence among female sex workers in Rwanda | Not included | Duplicate |
|  | Prevalence of Hepatitis B Virus (HBV) surface antigen and HBVassociated hepatocellular carcinoma in Kenyans of various ages | Not included | Duplicate |
|  | A nationwide cross-sectional review of in-hospital hepatitis B virus testing and disease burden estimation in Ghana, 2016 - 2021 | Not included | Duplicate |
|  | Studies on prevalence and risk factors for Hepatitis B Surface Antigen among secondary school students in north-central, Nigeria | Not included | Duplicate |
|  | Multidimensional Analysis of the Mother-to-child Transmission Risk Factors in Chronic Hepatitis B Virus Infection in Pregnant Women in Vietnam | Not included | Duplicate |
|  | Healthcare resource utilization and costs by disease severity in an insured national sample of US patients with chronic hepatitis B | Not included | Duplicate |
|  | Advancing Age and Comorbidity in a US Insured Population-Based Cohort of Patients With Chronic Hepatitis B | Not included | Duplicate |
|  | Hepatitis B‐related hepatocellular carcinoma: epidemiological characteristics and disease burden | Not included | Duplicate |
|  | An enormous hepatitis B virus‐related liver disease burden projected in Vietnam by 2025 | Not included | Duplicate |
|  | Hepatitis B in healthcare personnel: an update on the global landscape | Not included | Duplicate |
|  | Prevalence, infectivity and correlates of hepatitis B virus infection among pregnant women in a rural district of the Far North Region of Cameroon | Not included | Duplicate |
|  | Assessing risk behaviors and prevalence of sexually transmitted and blood-borne infections among female crack cocaine users in Salvador-Bahia, Brazil | Not included | Duplicate |
|  | Standard Precautions among HealthCare Workers in a Tertiary Health Facility in Enugu Metropolis, South-East Nigeria | Not included | Duplicate |
|  | HIV viraemia during hepatitis B vaccination shortens the duration of protective antibody levels | Not included | Duplicate |
|  | Viral load is a significant prognostic factor for hepatitis B virus‐associated hepatocellular carcinoma | Not included | Duplicate |
|  | Dual positivity of hepatitis B surface antigen and anti-hepatitis C virus antibody and associated factors among apparently healthy patients of Ekiti State, Nigeria | Not included | Duplicate |
|  | Hepatitis C virus infection and its associated factors among prisoners in a Nigerian prison | Not included | Duplicate |
|  | Early cranial ultrasound findings among infants with neonatal encephalopathy in Uganda: an observational study | Not included | Duplicate |
|  | Sero-prevalence of hepatitis B virus and associated factors among pregnant women in Gambella hospital, South Western Ethiopia: facility based cross-sectional study | Not included | Duplicate |
|  | Clinical characteristics and current management of hepatitis B and C in China | Not included | Duplicate |
|  | No contribution of lifestyle and environmental exposures to gender discrepancy of liver disease severity in chronic hepatitis b infection: Observations from the Haimen City cohort | Not included | Duplicate |
|  | Sero-Prevalence of Hepatitis B Virus Infection and Associated Factors Among Pregnant Women Attending Antenatal Care Services in Gedeo Zone, Southern Ethiopia | Not included | Duplicate |
|  | Effects of long-term antiretroviral therapy in reproductive-age women in sub-Saharan Africa (the PEPFAR PROMOTE study): a multi-country observational cohort study | Not included | Duplicate |
|  | Prevalence, genotype distribution and mutations of hepatitis B virus and the associated risk factors among pregnant women residing in the northern shores of Persian Gulf, Iran | Not included | Duplicate |
|  | Global Estimates on Biological Risks at Work | Not included | Duplicate |
|  | Telbivudine treatment of hepatitis B virus-infected pregnant women at different gestational stages for the prevention of mother-to-child transmission: Outcomes of telbivudine treatment during pregnancy | Not included | Duplicate |
|  | Occult Hepatitis B Virus Infection in Maintenance Hemodialysis Patients: Prevalence and Mutations in "a" Determinant | Not included | Duplicate |
|  | Prevalence of hepatitis B virus infection in Shenzhen, China, 2015–2018 | Not included | Duplicate |
|  | Identifying, preventing and controlling needle-stick injuries in Indonesia | Not included | Duplicate |
|  | Splash of body fluids among healthcare support staff in Ghana: a cross-sectional study | Not included | Duplicate |
|  | Physiological and psychosocial stressors among hemodialysis patients in the Buea Regional Hospital, Cameroon | Not included | Duplicate |
|  | Safety practice and associated factors among waste handlers in Governmental Hospitals in Addis Ababa, Ethiopia | Not included | Duplicate |
|  | Hepatitis B virus infection and associated risk factors among medical students in eastern Ethiopia | Not included | Duplicate |
|  | Risk factors associated with Hepatitis B virus infection among pregnant women attending public hospitals in Addis Ababa, Ethiopia | Not included | Duplicate |
|  | Clinicopathological analysis of patients with dual malignancies: A retrospective study | Not included | Duplicate |
|  | Prevalence and the associated factors of hepatitis B and hepatitis C viral infections among HIV-positive individuals in same-day antiretroviral therapy initiation program in Bangkok, Thailand | Not included | Duplicate |
|  | Prevalence of liver steatosis in patients with chronic hepatitis B: a study of associated factors and of relationship with fibrosis | Not included | Study done in other population |
|  | Hepatitis B, hepatitis C, and mortality among HIV-positive individuals | Not included | Study done in other population |
|  | Hepatitis B virus infection, associated factors, knowledge and vaccination status among household contacts of hepatitis B index cases in Mwanza, Tanzania | Not included | Study done in other population |
|  | Differential hepatic features presenting in Wilson disease-associated cirrhosis and hepatitis B-associated cirrhosis | Not included | Outcome not related |
|  | Novel approach to identifying the hepatitis B virus pre-S deletions associated with hepatocellular carcinoma | Not included | Outcome not related |
|  | Altered oral microbiota in chronic hepatitis B patients with different tongue coatings | Not included | Outcome not related |
|  | Prevalence and factors associated with hepatitis B virus infection among household members: a cross-sectional study in Beijing | Not included |  |
|  | Hepatitis E virus superinfection impairs long-term outcome in hospitalized patients with hepatitis B virus-related decompensated liver cirrhosis | Not included | Outcome not related |
|  | Decreased liver stiffness by transient elastography indicates lower incidence of hepatocellular carcinoma in patients with chronic hepatitis B | Not included | Unrelated study |
|  | Combination therapy based on pegylated interferon alfa improves the therapeutic response of patients with chronic hepatitis B who exhibit high levels of hepatitis B e-antigen at 24 weeks: A retrospective observational study | Not included | Unrelated study |
|  | Dynamics of Genotypic Mutations of the Hepatitis B Virus Associated With Long-Term Entecavir Treatment Determined With Ultradeep Pyrosequencing: A Retrospective Observational Study | Not included | Outcome not related |
|  | Primary Squamous Cell Carcinoma of Liver: Case Series and Review of Literatures | Not included | Outcome not related |
|  | Red cell distribution width-to-lymphocyte ratio: A novel predictor for HBV-related liver cirrhosis | Not included | Outcome not related |
|  | Potential resistant mutations within HBV reverse transcriptase sequences in nucleos(t)ide analogues-experienced patients with hepatitis B virus infection | Not included | Outcome not related |
|  | Risk factors associated with hepatic osteopathy in HBV related cirrhosis measured by liver stiffness: An Observational study | Not included | Outcome not related |
|  | High HBV-DNA serum levels are associated with type 2 diabetes in adults with positive HBsAg: An observational study | Not included | Unrelated study |
|  | Changing incidence of reported viral hepatitis in China from 2004 to 2016: an observational study | Not included | Study done in other setting |
|  | The impact of recipient age on the effects of umbilical cord mesenchymal stem cells on HBV-related acute-on-chronic liver failure and liver cirrhosis | Not included | Outcome not related |
|  | Relationship Between Platelets and the Clinical Efficacy of Umbilical Cord Mesenchymal Stem Cells for HBV-Related Acute-on-Chronic Liver Failure and Liver Cirrhosis: A Preliminary Clinical Study | Not included | Outcome not related |
|  | Association of Hepatitis C and B Virus Infection with CKD and Impact of Hepatitis C Treatment on CKD | Not included | Outcome not related |
|  | Th17 cells over 5.9% at admission indicate poor prognosis in patients with HBV-related acute-on-chronic liver failure | Not included | Unrelated study |
|  | Complement Factor 3 Could Be an Independent Risk Factor for Mortality in Patients with HBV Related Acute-on-Chronic Liver Failur | Not included | Outcome not related |
|  | HIV, hepatitis B virus, and hepatitis C virus co-infection in patients in the China National Free Antiretroviral Treatment Program, 2010-12: a retrospective observational cohort study | Not included | Study done in other setting |
|  | Corticosteroid treatment of patients with coronavirus disease 2019 (COVID-19) | Not included | Unrelated study |
|  | Effectiveness of tenofovir or telbivudine in preventing HBV vertical transmission for pregnancy | Not included | Outcome not related |
|  | Epidemiology of hepatitis B virus infection: results from a community-based study of 0.15 million residents in South China | Not included | Study done in other setting |
|  | Sero-prevalence and risk factors of hepatitis B virus and human immunodeficiency virus infection among pregnant women in Bahir Dar city, Northwest Ethiopia: a cross sectional study | Not included | Study done in other population |
|  | Prevalence and Associated Factors of Human Papillomavirus Infection among Iraqi Women | Not included | Unrelated study |
|  | Hepatitis C Virus Cascades of Care in the era of Direct-Acting Antiviral Therapy | Not included | Unrelated study |
|  | Maternal health in China - challenges of the next decade | Not included | Outcome not related |
|  | Epidemiological features of and changes in incidence of infectious diseases in China in the first decade after the SARS outbreak: an observational trend study | Not included | Study done in other setting |
|  | Effectiveness of telbivudine antiviral treatment in patients with hepatitis B virus-associated glomerulonephritis: A 104-week pilot study | Not included | Outcome not related |
|  | A Pilot Study of MicroRNAs Expression Profile in Serum and HBsAg Particles: Predictors of Therapeutic Vaccine Efficacy in Chronic Hepatitis B Patients | Not included | Outcome not related |
|  | Epidemiology of hepatitis B virus infection in Bangladesh: prevalence among general population, risk groups and genotype distribution | Not included | Study done in other population |
|  | Role of quantitative hepatitis B surface antigen in predicting inactive carriers and HBsAg seroclearance in HBeAg-negative chronic hepatitis B patients | Not included | Study done in other population |
|  | Immune response pattern varies with the natural history of chronic hepatitis B | Not included | Outcome not measured clearly |
|  | Mutations in pre-core and basic core promoter regions of hepatitis B virus in chronic hepatitis B patients | Not included | Outcome not measured clearly |
|  | Cirrhosis and liver transplantation in patients co-infected with HIV and hepatitis B or C: an observational cohort study | Not included | Outcome not measured clearly |
|  | The prevalence of hepatitis B virus infection in the United States in the era of vaccination | Not included | Study done in other setting |
|  | Global Burden and Trends of Primary Liver Cancer Attributable to Comorbid Type 2 Diabetes Mellitus Among People Living with Hepatitis B: An Observational Trend Study from 1990 to 2019 | Not included | Outcome not measured clearly |
|  | Population-Based Multicentric Survey of Hepatitis B Infection and Risk Factors in the North, South, and Southeast Regions of Brazil, 10-20 Years After the Beginning of Vaccination | Not included | Outcome not measured clearly |
|  | HBV pgRNA profiles in Chinese HIV/HBV coinfected patients under pre- and posttreatment: a multicentre observational cohort study | Not included | Outcome not measured clearly |
|  | Predictive value of serum ALT and T-cell receptor beta variable chain for HBeAg seroconversion in chronic hepatitis B patients during tenofovir treatment | Not included | Outcome not measured clearly Outcome not measured clearly |
|  | Evaluation and clinical significance of contrast-enhanced ultrasound on changes in liver blood flow perfusion after TIPS surgery | Not included | Outcome not measured clearly |
|  | Relationship of Treg/Th17 balance with HBeAg change in HBeAg-positive chronic hepatitis B patients receiving telbivudine antiviral treatment: A longitudinal observational study | Not included | Outcome not measured clearly |
|  | The association of adverse outcomes in the mother with disease progression in offspring in families with clusters of hepatitis B virus infection and unfavorable prognoses in Northwest China | Not included | Outcome not measured clearly |
|  | Prognostic value of immunoscore to identify mortality outcomes in adults with HBV-related primary hepatocellular carcinoma | Not included | Outcome not measured clearly |
|  | Clinical characteristics, prognosis, and surgical outcomes of patients with non-HBV and non-HCV related hepatocellular carcinoma: three-decade observational study | Not included | Outcome not measured clearly |
|  | Prevalence of and risk factors for hepatitis C virus antibody among people who inject drugs in Cambodia: a national biological and behavioral survey | Not included | Outcome not related |
|  | Health behaviors of Korean adults with hepatitis B: Findings of the 2016 Korean National Health and Nutrition Examination Survey | Not included | Study done in other setting |
|  | Somatosensory Amplification, Anxiety, and Depression in Patients With Hepatitis B: Impact on Functionality | Not included | Outcome not related |
|  | HCC risk reduction with oral nucleos(t)ide analogues in patients with chronic hepatitis B: Not perfect, not good enough | Not included | Outcome not related |
|  | Conservation and variability of hepatitis B core at different chronic hepatitis stages | Not included | Outcome not related |
|  | Seroprevalence and predictors of hepatitis B virus infection among pregnant women attending routine antenatal care in Arba Minch Hospital, South Ethiopia | Not included | Outcome not related |
|  | A study on prevention of bleeding complications using lusutrombopag for safe RFA in patients with hepatocellular carcinoma with low platelet counts: prospective observational study | Not included | Outcome not related |
|  | Cardiac health in patients with hepatitis B virus-related cirrhosis | Not included | Outcome not related |
|  | Health Profiles of Newly Arrived Refugee Children in the United States, 2006-2012 | Not included | Outcome not related |
|  | Impact of Occupational Hazards on Healthcare Professionals' Mental (Psychological) Health: Evidence from Government-Owned Hospitals in Khulna, Bangladesh | Not included | Outcome not related |
|  | Sharps injuries among health care workers in Cairo University Hospitals | Not included | Outcome not measured clearly |
|  | Predictors of acute coronary syndrome in pre hospital patients with chest pain | Not included | Study done in other population |
|  | Effects of Decreased Immunization Coverage for Hepatitis B Virus Caused by COVID-19 in World Health Organization Western Pacific and African Regions, 2020 | Not included | Outcome not related |
|  | Impact of the implementation of a vaccination strategy on hepatitis B virus infections in China over a 20-year period | Not included | Study done in other setting |
|  | Impact of hepatitis B vaccination on acute hepatitis B epidemiology in European Union/European Economic Area countries, 2006 to 2014 | Not included | Outcome not related |
|  | Global Perspectives on the Hepatitis B Vaccination: Challenges, Achievements, and the Road to Elimination by 2030 | Not included | Outcome not related |
|  | The worldwide impact of vaccination on the control and protection of viral hepatitis B | Not included | Outcome not related |
|  | Epidemiology of Hepatitis B Virus Infection and Impact of Vaccination on Disease | Not included | Outcome not measured clearly |
|  | The global impact of vaccination against hepatitis B: A historical overview | Not included | Outcome not related |
|  | Risk and Predictors of Mortality Associated With Chronic Hepatitis B Infection | Not included | Outcome not related |
|  | Risk factors for the mortality of hepatitis B virus-associated acute-on-chronic liver failure: a systematic review and meta-analysis | Not included | Outcome not related |
|  | Causes of death in people with chronic HBV infection: A population-based cohort study | Not included | Outcome not related |
|  | Trends in the disease burden of HBV and HCV infection in China from 1990-2019 | Not included | Study done in other setting |
|  | Worldwide prevalence of hepatitis B virus and hepatitis C virus among patients with cirrhosis at country, region, and global levels: a systematic review | Not included | It is not a primary study |
