# Supplementary material for: Seroprevalence and risk factors of hepatitis B virus infection among healthcare workers in Africa: A systematic review and meta-analysis
Source: PLoS One. 2025 Mar 25;20(3):e0319986. doi: 10.1371/journal.pone.0319986 (PMC11936272; doi:10.1371/journal.pone.0319986)
Supplement: S4 Table — (DOCX) [file pone.0319986.s004.docx]

**List of articles extracted for the study**

| Sr. No | Title of the articles | Name of data extractor | Date of extraction | Inclusion status |
| --- | --- | --- | --- | --- |
|  | Burden of Hepatitis-B Infections and Risk Factors among Healthcare Workers in Resource Limited Setting, Addis Ababa, Ethiopia | Leykun Berhanu | 12 August 2024 | Included |
|  | Knowledge, attitude and prevalence of hepatitis B virus among healthcare workers: a cross- sectional, hospital based study in Bamenda Health District, NWR, Cameroon | Leykun Berhanu | 12 August 2024 | Included |
|  | Seroprevalence of Hepatitis B Surface Antigen and Occupational Risk Factors Among Health Care Workers in Ekiti State, Nigeria | Leykun Berhanu | 12 August 2024 | Included |
|  | The exposure rate to hepatitis B and C viruses among medical waste handlers in three government hospitals, southern Ethiopia | Leykun Berhanu | 13 August 2024 | Included |
|  | Serological Evidence and Associated Factors of Hepatitis B Virus and Hepatitis C Virus Among Waste Handlers: A Cross-Sectional Study from Northeastern Ethiopia | Leykun Berhanu | 14 August 2024 | Included |
|  | Prevalence of hepatitis B virus markers in surgeons in Lagos, Nigeria | Leykun Berhanu | 14 August 2024 | Included |
|  | Hepatitis B infection among health workers in Uganda: evidence of the need for health worker protection | Leykun Berhanu | 15 August 2024 | Included |
|  | Seroprevalence of Hepatitis B virus infection and associated factors among health care workers in Southern Ghana | Leykun Berhanu | 15 August 2024 | Included |
|  | Seroprevalence of hepatitis B surface antigenaemia among healthcare worker in a private Nigerian tertiary health institution | Leykun Berhanu | 16 August 2024 | Included |
|  | Sero-Prevalence and Associated Factors of Hepatitis B Virus Infection among Health Professionals in Adama Town, Oromia, Central Ethiopia | Leykun Berhanu | 16 August 2024 | Included |
|  | Hepatitis B and C seroprevalence among health care workers in a tertiary hospital in Rwanda | Abebe Kassa Geto | 14 August 2024 | Included |
|  | Prevalence of hepatitis B virus infection and uptake of hepatitis B vaccine among healthcare workers, Makueni County, Kenya 2017 | Abebe Kassa Geto | 14 August 2024 | Included |
|  | Assessing the prevalence of hepatitis B virus infection among health care workers in a referral hospital in Kisantu, Congo DR: a pilot study | Abebe Kassa Geto | 14 August 2024 | Included |
|  | Prevalence of hepatitis B virus and immunity status among healthcare workers in Beira City, Mozambique | Abebe Kassa Geto | 14 August 2024 | Included |
|  | Seroprevalence and Knowledge of Hepatitis B Virus Infection Among LaboratoryWorkers at Kilimanjaro Christian Medical Centre in Moshi, Tanzania | Abebe Kassa Geto | 15 August 2024 | Included |
|  | Cross sectional study of chronic hepatitis B prevalence among healthcare workers in an urban setting, Sierra Leone | Abebe Kassa Geto | 15 August 2024 | Included |
|  | Hepatitis B Virus and Human Immunodeficiency Virus Infections among Health Care Workers in Some Health Care Centers in Benue State, Niger | Abebe Kassa Geto | 15 August 2024 | Included |
|  | Hepatitis B virus infection status and associated factors among health care workers in selected hospitals in Kisumu County, Kenya: A cross-sectional study | Abebe Kassa Geto | 15 August 2024 | Included |
|  | Prevalence of HBV, HCV and Associated Risk Factors Among Cleaners at Selected Public Health Centers in Addis Ababa, Ethiopia | Abebe Kassa Geto | 17 August 2024 | Included |
|  | Hepatitis B virus infection and its associated factors among medical waste collectors at public health facilities in eastern Ethiopia: a facility-based cross-sectional study | Abebe Kassa Geto | 17 August 2024 | Included |
|  | Seroprevalence of Hepatitis B and C among health care workers in Omdurman, Sudan | Gete Berihun | 13 August 2024 | Included |
|  | Prevalence and associated knowledge of hepatitis B infection among healthcare workers in Freetown, Sierra Leone | Gete Berihun | 13 August 2024 | Included |
|  | Seroprevalence of Hepatitis B and C among Healthcare Workers in Dutse Metropolis Jigawa State, Nigeria | Gete Berihun | 13 August 2024 | Included |
|  | Seroprevalence of hepatitis B virus infection and associated factors among healthcare workers in northern Tanzania | Gete Berihun | 14 August 2024 | Included |
|  | Sero-prevalence of hepatitis B virus infection and associated factors among health care workers and medical waste handlers in primary hospitals of North-west Ethiopia | Gete Berihun | 14 August 2024 | Included |
|  | Sero-prevalence and risk factors for hepatitis B virus infection among health care workers in a tertiary hospital in Uganda | Gete Berihun | 14 August 2024 | Included |
