# Supplementary material for: Seroprevalence and risk factors of hepatitis B virus infection among healthcare workers in Africa: A systematic review and meta-analysis
Source: PLoS One. 2025 Mar 25;20(3):e0319986. doi: 10.1371/journal.pone.0319986 (PMC11936272; doi:10.1371/journal.pone.0319986)
Supplement: Attachment — Submitted filename: Response to reviewers.docx [file pone.0319986.s005.docx]

**Response for Reviewer #1**

This study aimed to investigate the seroprevalence and risk factors for hepatitis B infection in healthcare workers (HCW) in Africa. The authors included 26 studies in a systematic review and meta-analysis, reporting a very high seroprevalence of infection (17.5%; 95% CI 8.36-26.04). As risk factors for infection, the study describes age >40, lack of training and previous liver disease. The authors conclude that these factors should be prioritized in interventions designed to prevent and control the occurrence of the infection. Considering the high burden of the infection in the region, the high risk of infection in healthcare personnel, and the under-representation of African countries in studies about hepatitis B, I consider the theme extremely relevant for publication. I congratulate the authors for choosing this topic and for the effort to provide a relevant result for the African region. The authors have properly designed the systematic review and the meta-analysis. However, the description of methodology and results should be clearer, more detailed and organized. I strongly suggest that the authors provide more details about the studies included in the review and meta-analysis, particularly regarding methodological characteristics, as a table or as supplementary material. I also suggest that the discussion regarding risk factors for hepatitis B is improved, addressing potential biases, adding comparisons with data from HCW and from the general population, and avoiding obvious associations.

I have some comments, questions and suggestions for the authors.

General Response: Dear reviewer, we would like to thank for providing valuable feedback. We also want to thank for your appreciation and motivation which gives us strength and further energy to engage more on such issues. In this revised manuscript, we made the methodology and result section clearer and detail. The discussion and other sections were also revised considering the given suggestion.

Comment: abstract: Add the date when each source was last searched.

Response: Thank you for our comment. The last searching date has been included in the abstract section of the revised manuscript.

Comment: The risk of bias scale used in the study must be specified in the Abstract.

Response: Thank you for your comment. “Studies with a quality evaluation indicator score of 50% or above were included in this study”. This statement has been included in the revised manuscript.

Comment: The total number of participants must also be specified.

Response: Thank you. Among 26 studies selected for meta-analysis, 6983 participants were included. This sentence has been added on the revised manuscript.

Comment: Considering that the study evaluated seroprevalence at one moment in time, I suggest that the authors do not use the verb “develop” when referring to “having” hepatitis B. “Develop” is used several times throughout the text.

Response: Thank you for your comment. The word “develop” has been changed to “having” throughout the manuscript.

INTRODUCTION

Comment 1: Lines 63-66 should be revised, particularly regarding the proper use of the words “virus” and “infection”, which are used incorrectly (for example: “the most dangerous kind of viral hepatitis, known as hepatitis B virus”; “vaccine… to prevent this virus”).

Response 1: Thank you. The sentence is re-phrased to make it clear and concise for the reader.

Comment: Line 84: Use “donated blood”, instead of “given blood”.

Response: Thank you. We have accepted the comment. The phrase is correctly written us “donated blood” as shown on the revised manuscript.

Comment 3: Line 85: Use “places” (plural form).

Response 3: Thank you. The noun is correctly written in its plural form.

Comment 4: Line 93: Please correct HIV as human immunodeficiency virus.

Response 4: We have accepted the comment. Hence, the short form of HIV is written fully on the revised manuscript.

Comment 5: Line 121: Use “children”, instead of “kids”.

Response 5: The noun “kids” is changed to “children” on the revised manuscript.

Comment 6: Lines 129-130: What are the differences between factors influencing HBV prevalence in previous studies?

Response 6: Thank you for your comment. As far as our knowledge and experience concerned, there is no a systematic review and meta-analysis study focusing on identifying the factors affecting the prevalence of HBV among HCW in Africa. In these lines we want to convey this idea. Lack of such study is one of the justification (gaps) for doing the current study.

Comment 7: Lines 146-147: As presented, this study does not assess or “highlight disparities in vaccination and treatment access”.

Response 7: Thank you for your comment. The sentence is deleted from the revised manuscript.

Comment 8: Lines 154-155: I suggest that the authors rewrite these phrases. The results of the present study can be relevant in several ways. However, saying that “the study can foster collaboration among researchers” and promote “data sharing and joint initiatives” is outside the scope of the objectives and methodology used.

Response 8: Thank you for pointing out this important point. We have accepted the comment and hence the sentence is deleted from the revised manuscript.

Comment 9: Lines 157-158: The objectives of the study must be better stated. According to the Abstract, the study also aimed to evaluate the risk factors of hepatitis B among HCW.

Response 9: Thank you. Correction has been made based on the given comments.

MATERIALS AND METHODS

Comment 1: Further details about the search strategies should be included in the text or as supplementary files, particularly the specific search terms, dates of publication, language, and any filters used in the search.

Response 1: Dear reviewer, thank you for your comment. Detail on search process and restriction applied during searching was included on the revised manuscript.

Comment 2: Lines 175-176: The list of HCW is already detailed in lines 195-200.

Response 2: Thank you. The list of healthcare professional was deleted since it is explain under the operational definition section.

Comment 3: Line 177: Use “were”, instead of “was included”.

Response 3: The verb “was” changed to “were” on the revised manuscript.

Comment 4: Line 181: I believe the authors mean “conducted”, instead of “published”.

Response 4: Thank you very much. It was to mean conducted. Hence, correction has been made.

Comment 5: Were there any other exclusion criteria? Figure 1 mentions animal studies, language, and methodological quality.

Response 5: Yes, these are also the exclusion criteria. We have included all the mentioned criteria under the exclusion criteria section of the revised manuscript.

Comment 6: Line 186: Add that 95% confidence intervals (CI) were calculated.

Response 6: We added that 95% CI was calculated on the revised manuscript.

Comment 7: Line 192: Repeated words.

Response 7: Repeated word has been deleted.

Comment 8: All data presented in the Results must be mentioned here, including all predictors that were evaluated in the study (and the specific definition, if necessary).

Response 8: Thank you for your comment. Since the work is a systematic review and meta-analysis study, not a primary study, we define important terms and variables only. For example, outcome variables and HCWs are defined. The remaining may not need definition, since they are understandable by the readers and the study aim.

Comment 9: How many reviewers evaluate the data from each paper?

Response 9: Two reviewers have evaluated each paper and additional reviewer was also invited to handle disagreement.

Comment 10: Line 218: “Perform” should be in past tense.

Response 10: The word “perform” is changed to “performed” on the revised manuscript.

Comment 11: Line 220: Low risk of what?

Response 11: Thank you. It was to mean “low risk of quality”. Hence, correction has been made.

Comment 12: Line 222: There seems to be missing words in this phrase.

Response 12: Thank you. We agreed that the sentence is incomplete. Hence, correction has been made by completing the sentence as “Studies with a quality evaluation indicator score of 50% or above were included in the study”.

Comment 13: Did all reviewers evaluate each different paper?

Response 13: Yes, all reviewers evaluate each paper

Comment 14: Line 229: Please correct “I2”.

Response 14: Than you. We apologize for such typing error. It is correctly written to I^2^ on the revised manuscript.

Comment 15: Line 232: This line is missing a full stop.

Response 15: Thank you. Full stop has been added as shown on the revised manuscript.

Comment 16: Please add detailed descriptions of sensitivity analysis, subgroup analyses, and meta-regression, as well as information regarding handling of missing data.

Response 16: Detailed descriptions of sensitivity analysis, subgroup analyses, and meta-regression were included in the revised manuscript. Missing data was managed using sensitivity analysis, trim and fill analysis. Moreover, restricted random effect (REML) model and sensitivity analysis was used.

RESULTS

Comment 1: Lines 236-237: “Result” and “study” should be in plural form.

Response 1: Thank you. The nouns are correctly written on the revised manuscript.

Comment 2: Line 243: Please rewrite the part about the values reported in Nigeria; the phrase is not clear.

Response 2: Thank you. There was also typing error. The highest prevalence of HBV was reported in Niger while the lowest was in Nigeria. Hence, the sentence is re-written as “The highest seroprevalence was reported in Niger with the rate of 86.7% (31) while the lowest was reported in Nigeria with the rate of 1.1% (29)” as shown on the revised manuscript.

Comment 3: Table 1: The column “Study design” is not necessary; the information that all studies are cross-sectional can be added to the text. The 95% CI for each study must be shown. Column “Prevalence of HBV”: is it shown in percentages?

Response 3: Thank you for your comment. We have deleted the column for study design. The included studies did not report 95% CI for HBV prevalence. Therefore, the approximated 95% CI was calculated considering the sample size and the reported prevalence value. The prevalence value is reported in percentage. Hence, (%) is added under the “Prevalence of HBV” column as shown on the revised manuscript.

Comment 4: Lines 251-252: The choice for a random effect model should be presented and justified in the Methods section.

Response 4: Thank you for your comment. Under the method section, it was stated that the presence and level of heterogeneity among the included studies was calculated using I^2^ with its corresponding p-value. Due to the high level of heterogeneity (I^2^=99.7, p<0.001), we used a random effect model to calculate the pooled prevalence of HBV. These ideas are already included under data analysis and presentation sections of the manuscript. If there any concern we are happy to discuss further on the matter.

Comment 5: Line 274: Please mention and describe the trim and fill study in the Methods section.

Response 5: Thank you. We have accepted the comment. The trim and fill analysis is described under the data analysis and presentation section of the revised manuscript.

Comment 6: Line 299: Why did the authors choose the sample size of 269 for the subgroup analyses?

Response 6: Thank you for your concern. The criteria to use a sample size of 269 for sub group analysis are not also taken haphazardly. We have calculated the average sample size and used as bench mark to be used for sub group analysis.

Comment 7: Lines 331-336: The list of data extracted from the studies should be in the Methods section. Please be more specific regarding some items: diagnosed liver disease, mucocutaneous, hospitalization, dental procedure, contact history.

Response 7: Thank you for your comment. The list of data extracted from the included studies has been moved to the data extraction section of the revised manuscript. Regarding the terms like diagnosed liver disease, mucocutaneous, hospitalization, dental procedure, and contact history, those terms are directly taken from the primary studies included in this review. Their corresponding OR and 95% CI were taken to calculate the pooled factors affecting HBV infection.

Comment 8: Line 342: The text is not clear.

Response 8: Thank you. The sentence is re-written as “The results showed that the risk of contracting HBV infection was 5.01 times higher for healthcare workers with liver disease diagnoses than for those without” as shown on the revised manuscript.

Comment 9: Line 348: Please correct “The”.

Response 9: Thank you. Correction has been made on the revised manuscript.

Comment 10: Table 4: Please add the reference numbers of the studies in each line.

Response 10: We have accepted the comment. The reference number of each of the corresponding articles has been included in the revised manuscript.

DISCUSSION
Comment 1: Lines 361-364: This phrase is out of context here.

Response 1: Thank you. The sentence is deleted.

Comment 2: Lines 366-368: These phrases should be rewritten. Is this study part of “evaluating the progress towards achievement of the HBV eradication target”?

Response 2: Thank you for your comment. The objective is not to evaluate the HBV eradication progress. Hence, the sentence is re-written.

Comment 3: Lines 369-374: It should be clearer that this comparison refers to studies conducted in the general population.

Response 3: Thank you. As suggested by the reviewer, it was good to compare finding in similar target population, however, due to the absence of global prevalence study on prevalence of HBV among HCWs, we are obligated to compare it with the general population.

Comment 4: Lines 372-374: The use of parentheses must be revised. What were the 95% CIs reported in these studies? Can the authors surely state that these studies found lower prevalence of hepatitis B?

Response 4: Thank you. We have seen that the figure i.e. the reported prevalence of HBV and the figure written next to it inside other bracket create confusion. However, the figure with percentage indicates prevalence of HBV while the number kept inside the bracket showed the reference number. It was good to mention the 95% CI, however, the studies did not report it. Hence, unable to include the 95% CI. However, we are confident that the mentioned studies reported HBV prevalence lower than the present systematic review and meta-analysis study.

Comment 5: Line 374: Initiate a new paragraph.

Response 5: Thank you. We have started on the new paragraph.

Comment 6: Lines 398-402: Please provide the 95% CIs reported in the studies mentioned. I suggest that the authors carefully reflect about the comparisons made in this paragraph.

Response 6: Thank you for your comment. The 95% CI is not reported in the mentioned studies. Most studies reported the 95% CI for predictors not for the prevalence, hence unable to include it in the manuscript. As described earlier the present finding is compared with the finding from the previous report in various states and countries. Such comparison is common in literature due to the fact that the general population also included healthcare workers. The possible reason for the deviation then stated as “The possible reason for the variation may be attributed to the difference in target population” which is added in the revised manuscript.

Comment 7: Lines 423-435: As stated by the authors, the seroprevalence might increase with age simply because of longer exposure to hepatitis B, regardless of the area of work. What is the seroprevalence according to age in the general population?

Response 7: Thank you for your concern. In this study, the seroprevalence of HBV increased with age. It is not only because of longer exposure to hepatitis B but also because of the nature of working environment. Other reasons have also mentioned in the manuscript. Since our aim is to compare the pooled finding of HBV prevalence between different studies, the seroprevalence of HBV in the general population is not mentioned or is not important for our study.

Comment 8: Lines 437-445: Do the “underlying liver conditions” exclude acute/chronic hepatitis B? Please make sure that hepatitis B, one of the most common “liver conditions”, are not included in these analyses.

Response 8: We know that underlying liver conditions generally refers to liver diseases that are not specifically Hepatitis B, such as cirrhosis, fatty liver disease, or liver cancer. So, while "underlying liver conditions" typically excludes acute or chronic Hepatitis B, individuals with chronic Hepatitis B are already considered to have a liver condition. In this review, those with current infection, and current or past resolved infection were included. Therefore, phrase i.e. liver condition/diseases, highlights other liver issues that might coexist or compound the risks associated with Hepatitis B.

Comment 9: Lines 449-450: I suggest that the authors rewrite this phrase and explain the “unit of cumulative knowledge gain”.

Response 9: Thank you for your comment. The sentence is written to transmit the idea that having increased knowledge about HBV helps to enhance people’s willingness to screen. Whatever the unit (%, frequency or any other) is, increasing knowledge gain helps individual to screen for HBV, thereby to prevent the risk of acquiring HBV infection. However, we understand that the sentence is not clear for the reader. Hence, it is re-written on the revised manuscript.

Comment 10: Line 470: There is no previous mention to missing data - please specify how missing data were handled (Methods), and specify their occurrence (Results). Regarding the limitations of the present study, please consider methodological characteristics of the included studies, heterogeneity, and publication bias.

Response 10: Thank you for your comment. We understand that missing data is a little concern in systematic review and meta-analysis studies as compared to the primary studies. However, in this review, sensitivity analysis is used to provide insights into the robustness and reliability of the results. The use of sensitivity analysis is already included in method and result sections too. The limitation of the study is improved as per the comments given.

Comment 11: Line 486: Not only medical professionals – all HCW.

Response 11: we have revised the sentence as per the given suggestion.

Comment 12: Lines 495-498: Please be more specific. The association between the risk factors considered significant in the present study (age, liver disease, lack of training) and seropositivity for HBV is already well established.

Response 12: May be true. But, as far as we know, there is no systematic review and meta-analysis study done by including longitudinal studies useful to indicate trends in long term basis.

Comment 13: How do the authors explain the wide variation of seroprevalence in different African studies, particularly regarding the extreme values, which were both obtained in Nigeria? I suggest that the authors provide a summary of methodological characteristics of each study - as a table or as supplementary material.

Response 13: Thank you for your comment. It was typing error, sorry for that. The highest rate was reported in Niger while the lowest was reported in Nigeria. Hence, correction has been made. Regarding the methodological characteristics for each study, it is already included under the characteristics of the study section. Author year of publication, geographic location, sample size, prevalence, and detection method are all the relevant methodological characteristics already included.

Comment 14: What is the seroprevalence of hepatitis B in HCW from other regions of the world?

Response 14: Thank you for your concern. The seroprevalence of hepatitis B in HCWs for example global rate at 3.61% and in Europe 0.6% or even may be different across the country.

Comment 15: Which are the risk factors for seropositivity reported in previous studies?
Response 15: Thank you for your concern. Risk factors for HBV seropositivity include demographic factors (age, sex), geographic location, behavioral practices (drug use, sexual practices), occupational exposure, medical history, socioeconomic factors, family history, pregnancy, and existing chronic liver conditions. Understanding these risk factors can help target prevention and vaccination efforts effectively. However, those risk factors are not investigated well in the previous studies. This is why the present study is needed.

Comment 15: FIGURES 2, 9, 6, 7, 8: I suggest using smaller diamonds and expanding the horizontal axis, so that the 95% CI is more clearly visible.

Response 15: Thank you for your comment. All the figures were revised as per the given comments.

Comment 16: FIGURE 1: I suggest changing the text direction in the purple rectangles

Response 16: The figure is corrected as per the given suggestion.

**Response for reviewer #2**

Dear Authors Thanks for conducting this a valuable systemic review and meta-analysis “Seroprevalence and Risk Factors of Hepatitis B Virus Infection among Healthcare Workers in Africa: A systematic review and Meta-analysis”.

Introduction section

Comment 1: Paragraph 1 line 5. It is the first vaccination against hepatocellular carcinoma, a serious human cancer (2). My comment. Is the vaccine against hepatocellular carcinoma?

Response 1: Dear reviewer, thank you for your comment. Normally, the vaccine is primarily against HBV. Chronic infection with HBV is a significant risk factor for developing hepatocellular carcinoma. Hence, the vaccine prevents the occurrence of HBV which in turn leads to the occurrence of hepatocellular carcinoma. It means the vaccine helps to prevent the occurrence of both HBV and its long term consequence hepatocellular carcinoma.

Methodology section

Comment 2: Operational definitions: Presence of HBV infection: Is the presence of serum HBsAg (current infection) and/or anti HBC (current or past resolved infection) considered evidence for exposure to HBV (recent infection or chronic carrier). My comment What about other markers of HBV as Anti HBs to differentiate the past resolved infection from vaccinated HCWs in those positive for anti HBC.

Response 2: Thank you for your comment. It was good to differentiate the past resolved infection from vaccinated HCWs in those positive for anti HBC. However, all of the study measures only the current, past resolved infection or both occurrences. Since this study aims to measure the pooled prevalence of HBV from that primary study, it is not possible to differentiate the past resolved infection from vaccinated HCWs in those positive for anti HBC. Doing additional primary studies that differentiate past resolved infection from the vaccinated one will be one recommendation for future researcher.

Comment 3: In methods for detection of HBV there are 2 modalities used ELISA and CMIA. According to your comment, there is a significant difference between 2 modalities for detection of HBV which raise the issue of missed cases in diagnosis of HBV.

Response 3: Thank you for your comment. It is not possible to state that the difference in HBV rate reported using ELISA and CMIA are because of missed diagnosis. However, it implies that ELISA assays is designed to detect a broader range of serological markers, including low levels of antibodies or antigens, which could result in more positive test results. In addition, ELISA tests can sometimes have higher rates of cross-reactivity with antibodies from other infections or conditions, leading to false-positive results. This could inflate the reported infection rate. In all cases it is the characteristics of the included study.

Comment 4: It was mentioned that in result, the finding revealed that those who were jaundiced and diagnosed with liver disease were 5.01 times more likely to develop HBV infection as compared to those who were not exposed. Is the liver disease related to HBV or not? If not related what was the cause of previous liver diseases.

Response 4: Thank you for your concern. Liver disease is often related to HBV infection, which can lead to various liver conditions. However, if HBV is not a factor, liver disease can result from other viral infections, alcohol consumption, metabolic disorders, autoimmune diseases, genetic conditions, and drug-induced injury.

Comment 5: Were HCWs vaccinated or not?

Response 5: As a general rule, they should vaccinate it. But, in practice, they may not be vaccinated depending on the situation.
