# Supplementary material for: Seroprevalence and risk factors of hepatitis B virus infection among healthcare workers in Africa: A systematic review and meta-analysis
Source: PLoS One. 2025 Mar 25;20(3):e0319986. doi: 10.1371/journal.pone.0319986 (PMC11936272; doi:10.1371/journal.pone.0319986)
Supplement: Attachment — Submitted filename: Dear Authors.docx [file pone.0319986.s006.docx]

Dear Authors

Thanks for conducting this a valuable systemic review and metanalysis “Seroprevalence and Risk Factors of Hepatitis B Virus Infection among Healthcare Workers in Africa: A systematic review and Meta-analysis”.

Comment 1: Paragraph 1 line 5. It is the first vaccination against hepatocellular carcinoma, a serious human cancer

My comment. Is the vaccine against hepatocellular carcinoma? Response

1: Dear reviewer, thank you for your comment. Normally, the vaccine is primarily against HBV. Chronic infection with HBV is a significant risk factor for developing hepatocellular carcinoma. Hence, the vaccine prevents the occurrence of HBV which in turn leads to the occurrence of hepatocellular carcinoma. It means the vaccine helps to prevent the occurrence of both HBV and its long term consequence hepatocellular carcinoma.

Comment: please write that in the text clearly as it is mentioned in the introduction the vaccine is against hepatocellular carcinoma not against virus.
