# Supplementary material for: Seroprevalence and risk factors of hepatitis B virus infection among healthcare workers in Africa: A systematic review and meta-analysis
Source: PLoS One. 2025 Mar 25;20(3):e0319986. doi: 10.1371/journal.pone.0319986 (PMC11936272; doi:10.1371/journal.pone.0319986)
Supplement: Attachment — Submitted filename: Response_to_reviewers_auresp_2.docx [file pone.0319986.s007.docx]

**Response to Journal and reviewer’s comment**

**Journal Requirements:**

**Concern 1:** Please review your reference list to ensure that it is complete and correct. If you have cited papers that have been retracted, please include the rationale for doing so in the manuscript text, or remove these references and replace them with relevant current references. Any changes to the reference list should be mentioned in the rebuttal letter that accompanies your revised manuscript. If you need to cite a retracted article, indicate the article’s retracted status in the References list and also include a citation and full reference for the retraction notice.

**Response 1: The entire reference lists have been checked and correction has been made on volume and issue of the articles, page numbers, links to access the source, and date of access and other issues accordingly. In this study, as far our knowledge is concerned; a retracted article is not included.**

**Response for Reviewer’s comment**

**Reviewer #1: The authors have adequately addressed all my comments. I have only a few minor suggestions.**

**Response: Dear reviewer thank you very much for your insightful comments.**

Comment 1: INTRODUCTION: The phrase is still not clear. I suggest saying that “the symptoms of hepatitis B virus (HBV) infection include liver inflammation (…)”.
**Response: thank you so much. We have revised the phrase as per the given comments. The change is located on page 2 line 65.**

Comment 2: I suggest that the criteria used to choose the sample size of 269 is added to the text.
**Response 2: Thank you for your comment. “*Two categories were used to classify the sample size: less than 269 and higher than or equal to 269. The average sample size of the included studies serves as the basis for the categorization threshold”.* We believe that this idea better if included under the method section specifically on data analysis and presentation section of the revised manuscript. Therefore, the above sentences have been included under method section on page 13 line 274-276.**

Comment 3: Lines 492-494: Repeated phrase.

**Response 3: thank you. The repeated phrase is deleted from the revised manuscript.**

Comment 4: Figure 1: The edited figure was not presented in the revised manuscript.

**Response 4: We have accepted the comment. The corrected figures included in the revised manuscript.**

**Reviewer#2: Dear Authors Thanks for conducting this a valuable systemic review and meta-analysis “Seroprevalence and Risk Factors of Hepatitis B Virus Infection among Healthcare Workers in Africa: A systematic review and Meta-analysis”.**

Response Dear reviewer, thank you so much for your comment.

**Previous Comment 1:** Paragraph 1 line 5. It is the first vaccination against hepatocellular carcinoma, a serious human cancer. My comment. Is the vaccine against hepatocellular carcinoma?

**Previous Response 1:** Dear reviewer, thank you for your comment. Normally, the vaccine is primarily against HBV. Chronic infection with HBV is a significant risk factor for developing hepatocellular carcinoma. Hence, the vaccine prevents the occurrence of HBV which in turn leads to the occurrence of hepatocellular carcinoma. It means the vaccine helps to prevent the occurrence of both HBV and its long term consequence hepatocellular carcinoma.

**New Comment: please write that in the text clearly as it is mentioned in the introduction the vaccine is against hepatocellular carcinoma not against virus.**

**Response for new comment:** Thank you for your comment. First and foremost, rather than preventing hepatocellular cancer, the vaccine described in this paragraph helps prevent HBV infection. Since hepatocellular carcinoma develops after a prolonged period of chronic HBV infection, an individual will initially be infected with the virus. Therefore, the main purpose of the vaccine to which we wish to refer in this context is to prevent HBV infection. In addition, the major concern of the study is on HBV not on its long term consequence named hepatocellular carcinoma. Therefore, we decided to remove the sentence to prevent this kind of confusion.
